# Supplementary material for: SFPQ-TFE3 reciprocally regulates mTORC1 and induces lineage plasticity in a mouse model of renal tumorigenesis
Source: Nat Commun. 2025 Oct 3;16:8822. doi: 10.1038/s41467-025-63885-2 (PMC12494988; doi:10.1038/s41467-025-63885-2)
Supplement: Supplementary file 1 — Supplementary Information [file 41467_2025_63885_MOESM1_ESM.pdf]

Supplementary Information

SFPQ-TFE3 reciprocally regulates mTORC1 and induces lineage plasticity in a mouse model of renal tumorigenesis

Supplementary Figure 1

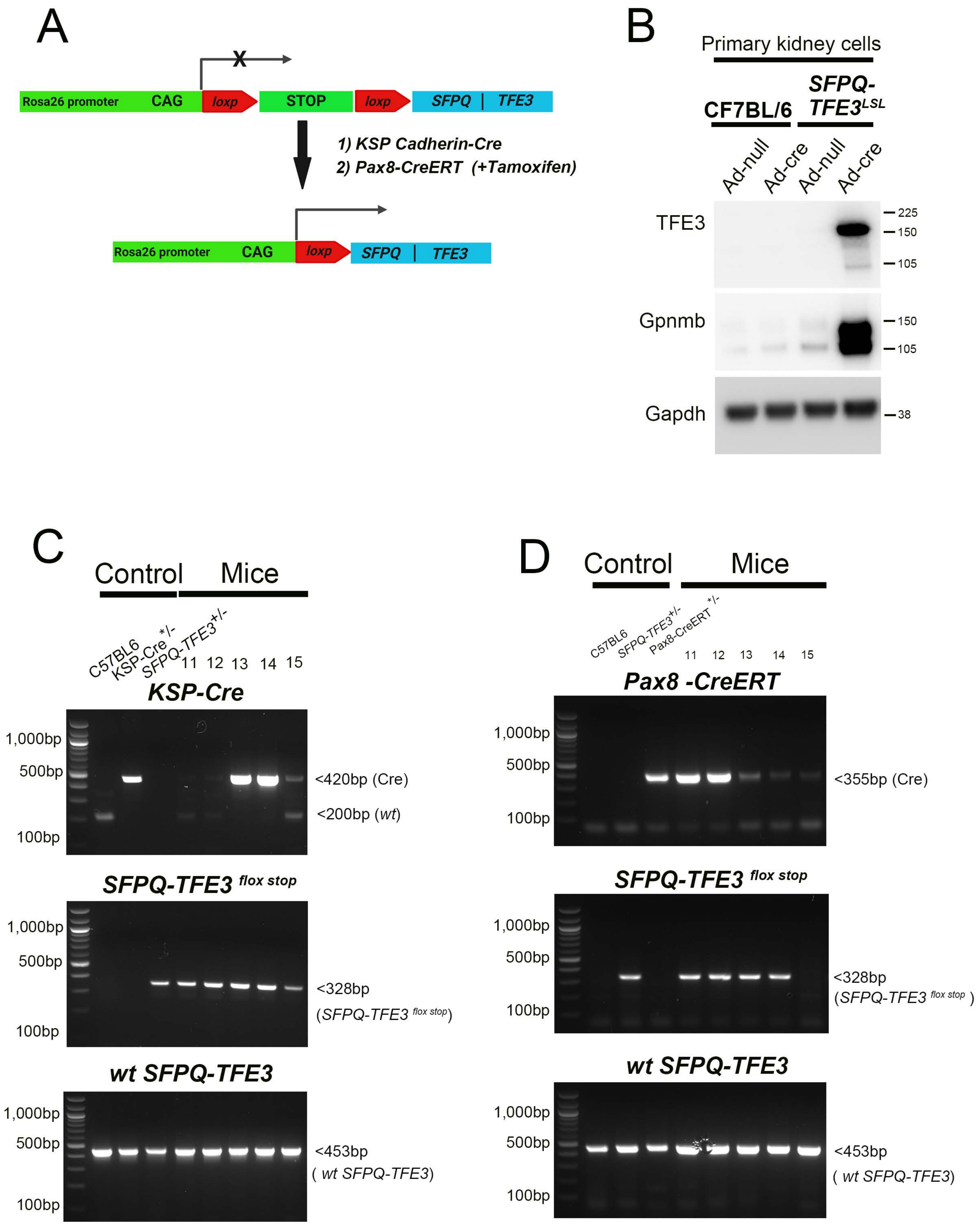

Supplementary Figure Legends

Supplementary Figure 1: ***Generation of an inducible murine allele of SFPQ-TFE3.* (A)** Schematic of the *SFPQ-TFE3* fusion transgene expression cassette. The human *SFPQ* CDS (exon 1-9) /human *TFE3* CDS (exon 5-10) (type 2 fusion), was cloned into intron 1 of the mouse *Rosa26* locus in reverse orientation, separated from the native CAG promoter by a stop sequence flanked by *LoxP* sites *(LSL).* Transgenic mouse expressing the human *SFPQ-TFE3* fusion protein, were crossed with either 1) *Ksp-Cadherin16 Cre* mice or 2) *Pax8-CreERT* mice (with tamoxifen treatment), enabling conditional excision of the stop sequence upon Cre-mediated recombination and expression of the fusion transgene. **(B)** Immunoblotting of lysates from primary renal tubular epithelial cells from wild-type C57BL6 mice or *SFPQ-TFE3*^{LSL} transgenic mice treated with control or Cre-recombinase expressing adenovirus *in vitro* for the indicated antibodies. Genotyping PCR from: **(C)** *SFPQ-TFE3*^{LSL}*; Ksp-Cre* mice or, **(D)** tamoxifen-treated, *SFPQ-TFE3*^{LSL}*; Pax8-CreERT* (right panels), with expected molecular weights of PCR products indicated on right. All experiments represent n ≥ 3 independent biological replicates. Source data are provided as a Source data file.

Supplementary Figure 2

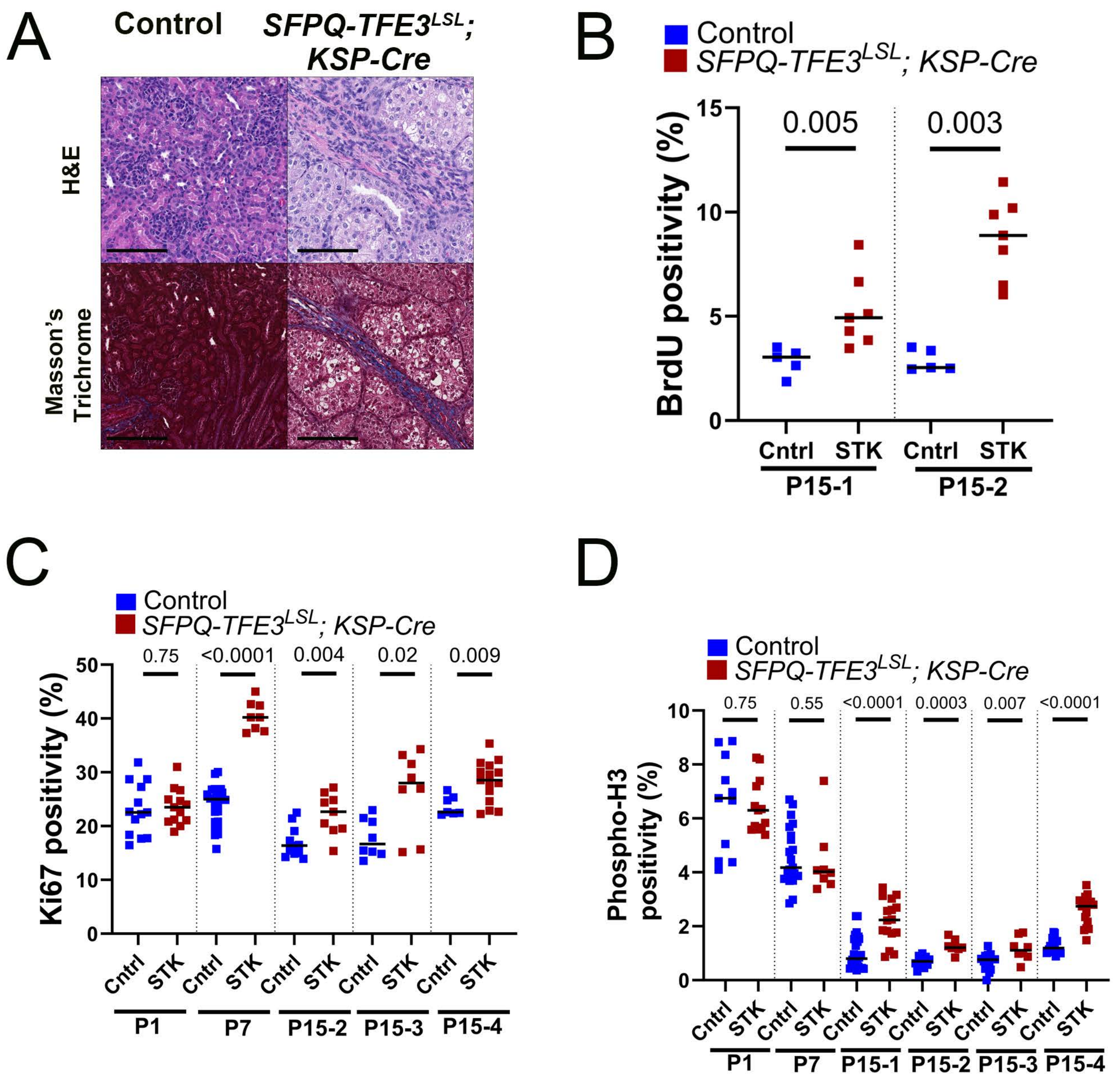

Supplementary Figure 2: *Ksp-Cadherin Cre-mediated induction of SFPQ-TFE3 disrupts kidney development with renal failure and early neonatal death.* **(A)** H&Es (top panels) and Masson's trichrome staining (bottom panels) showing features of inter-tubular fibrosis in *SFPQ-TFE3^{LSL}; Ksp-Cre* transgenic mice kidneys, compared to controls, at post-natal day 15. Scale bar=100 µm. **(B)** BrdU incorporation and positivity in kidneys of age-matched, control and *SFPQ-TFE3^{LSL}; Ksp-Cre* transgenic mice at post-natal day 15, as measured by an IHC assay. The following numbers were analyzed in kidney sections from 2 pairs of mice: P15-1 [control=2, STK=2] and P15-2 [control=1, STK=1]. Data are presented as median. P-values by two-tailed, Mann-Whitney test. **(C)** Ki67 positivity in kidneys of age-matched, control and *SFPQ-TFE3^{LSL}; Ksp-Cre* transgenic mice at post-natal days 1, 7 and 14, as measured by an IHC assay. P15 mice were analyzed in kidney sections from 3 separate mouse groups. The following numbers were analyzed: P1 [control=5, STK=6], P7 [control=2, STK=2], P15-2 [control=1, STK=1], P15-3 [control=2, STK=2], and P15-4 [control=3, STK=5]. Data are presented as median. P-values by two-tailed, Mann-Whitney test. **(D)** Phosphorylated-Histone H3 (pH3) positivity in kidneys of age-matched, control and *SFPQ-TFE3^{LSL}; Ksp-Cre* transgenic mice at post-natal days 1, 7 and 14, as measured by an IHC assay. P15 mice were analyzed in kidney sections from 4 separate mouse groups. The following numbers were analyzed: P1 [control=5, STK=6], P7 [control=2, STK=2], P15-1 [control=2, STK=2], P15-2 [control=1, STK=1], P15-3 [control=2, STK=2], and P15-4 [control=3, STK=5]. Data are presented as median. P-values by two-tailed, Mann-Whitney test. All experiments represent $n \geq 3$ independent biological replicates. Source data are provided as a Source data file.

Supplementary Figure 3

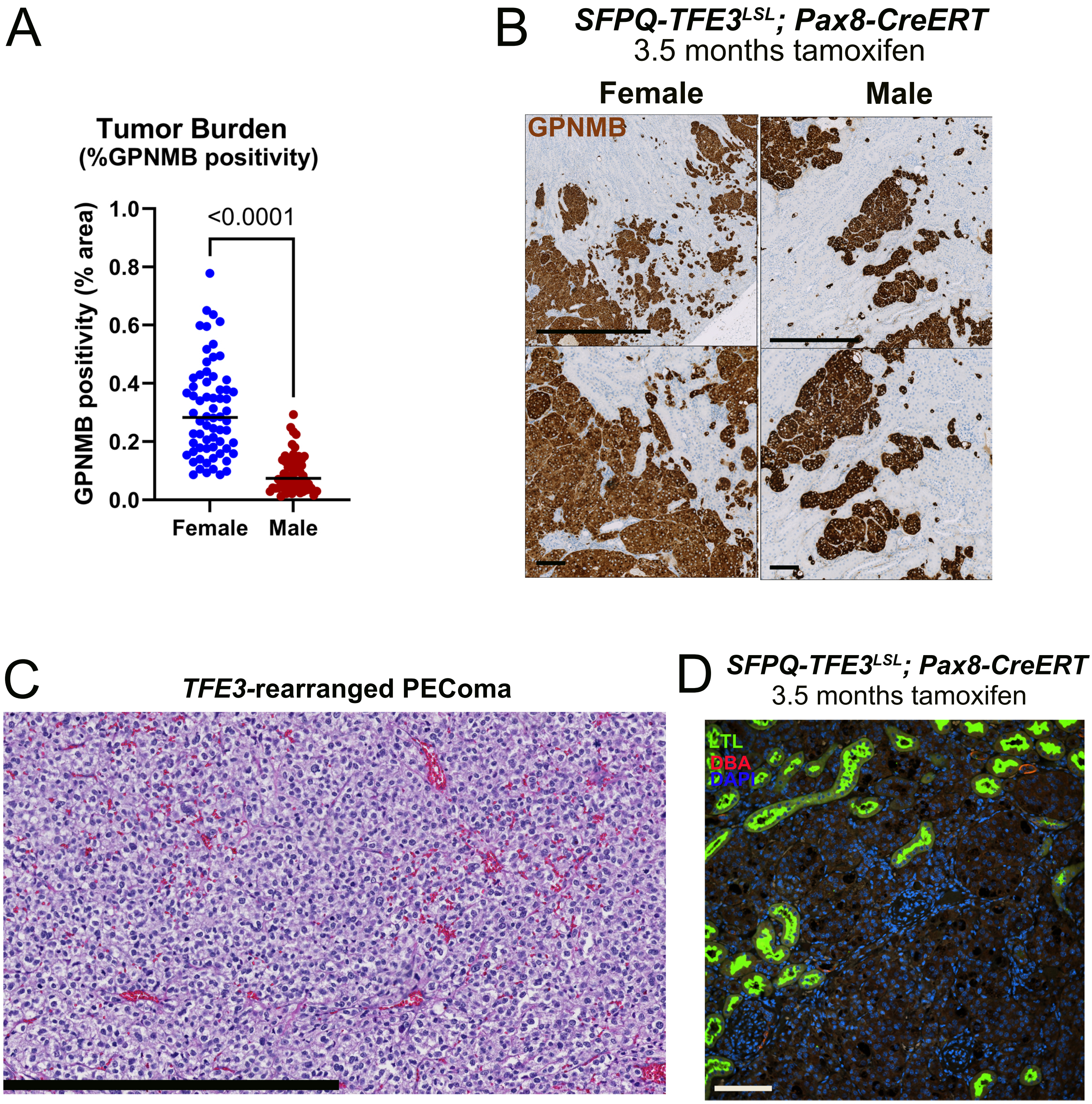

Supplementary Figure 3: ***Conditional post-natal, doxycycline-mediated induction of SFPQ-TFE3 in Pax8-CreERT mice induces renal tumor development.*** **(A)** Estimation of kidney tumor burden in female and male *SFPQ-TFE3*^{LSL}*; Pax8-CreERT* transgenic mice, sacrificed at 3.5 months following injection of tamoxifen. FFPE sections of kidneys were stained for GPNMB and the measured tumor area (assessed as % GPNMB positivity) was digitally quantified using HALO. The following numbers of ROIs were analyzed: [female=66, male=60]. Data are presented as median. P-values by two-tailed, Mann-Whitney test. Mice used in these analyses were from vehicle-treated, male and female cohorts from Vehicle/Torin1 experiments in Supplementary Fig. 11. (**B)** Representative IHC for GPNMB in female and male tamoxifen-injected, *SFPQ-TFE3*^{LSL}*; Pax8-CreERT* transgenic mice, from experiments in **(A).** Scale bar= 1mm (top left), 500 µm (top right) and 100 µm (bottom panels). **(C)** H&E of a human *TFE3*-rearranged PEComa with nests of monomorphic epithelioid cells with clear to eosinophilic cytoplasm and large round nuclei. **(D)** Indirect immunofluorescence for LTL (green) and DBA (red) in *SFPQ-TFE3*^{LSL}*; Pax8-CreERT* transgenic mice, sacrificed at 3.5 months following injection of tamoxifen. Scale bar=100 µm. All experiments represent n ≥ 3 independent biological replicates. Source data are provided as a Source data file.

Supplementary Figure 4

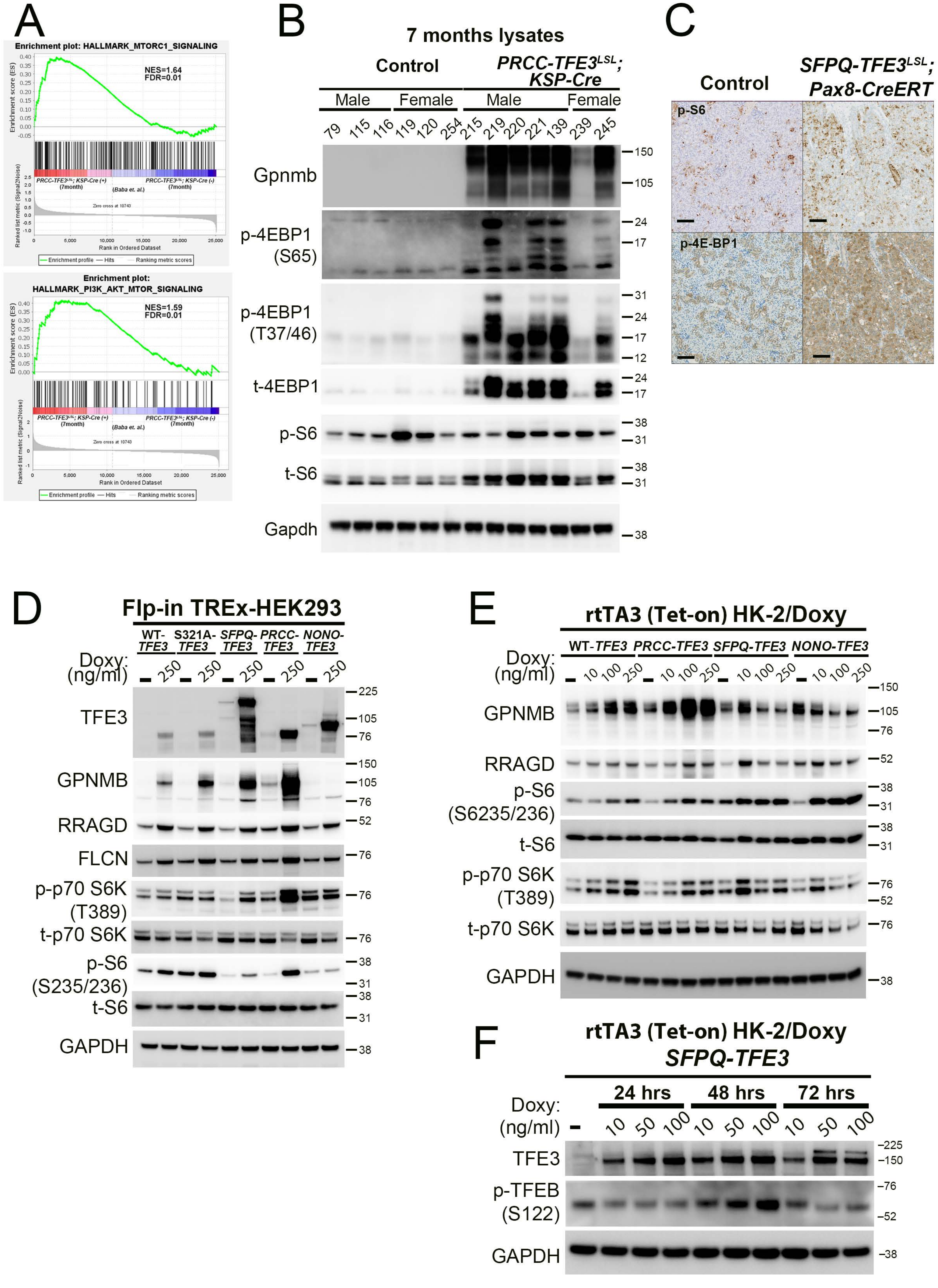

Supplementary Figure 4: ***mTORC1 signaling is activated in murine and human models of SFPQ-TFE3 fusion-RCC.*** **(A)** Gene Set Enrichment Analysis (GSEA) using Hallmark gene sets for MTORC1 signaling (upper panel) and PI3K/AKT/MTOR signaling (lower panel), in *PRCC-TFE3; KSP-Cre (+)* transgenic mice at 7 months, compared to controls[4]. **(B)** Immunoblotting of kidney lysates from control and *PRCC-TFE3; KSP-Cre (+)* transgenic mice at 7 months, for phosphorylation of mTORC1 substrates (p-4E-BP1[S65], p-4E-BP1[T37/46] and p-S6[S235/236]). **(C)** Representative immunohistochemistry (IHC) for p-S6[S235/236] (top row) and p-4E-BP1[T37/46] (bottom row) from age-matched, control and *SFPQ-TFE3*^{LSL}*; Pax8-CreERT* transgenic mice, sacrificed at 3.5 months following injection of tamoxifen. Scale bar= 100 μm. **(D)** Immunoblotting of lysates from HEK293 cells with doxycycline-inducible expression of *WT-TFE3*, *S321A-TFE3*, *SFPQ-TFE3, PRCC-TFE3* and *NONO-TFE3,* using the Flp-In-T-Rex™ system, for the indicated antibodies. Cells were untreated or treated with the indicated doses of doxycycline for 48 hrs, prior to lysis and immunoblotting. **(E)** Immunoblotting of lysates from HK2 proximal tubular epithelial cells, with doxycycline-inducible expression of *WT-TFE3* and *TFE3* fusion proteins using the rtTA3 (Tet-on) system, for the indicated antibodies. Cells were untreated or treated with the indicated doses of doxycycline for 72 hrs, prior to lysis and immunoblotting. **(F)** Immunoblotting of lysates from HK2/*SFPQ-TFE3* cells, treated with the indicated doses of doxycycline for 24, 48 or 72 hrs for expression of p-TFEB. All experiments represent $n \geq 3$ independent biological replicates. Source data are provided as a Source data file.

Supplementary Figure 5

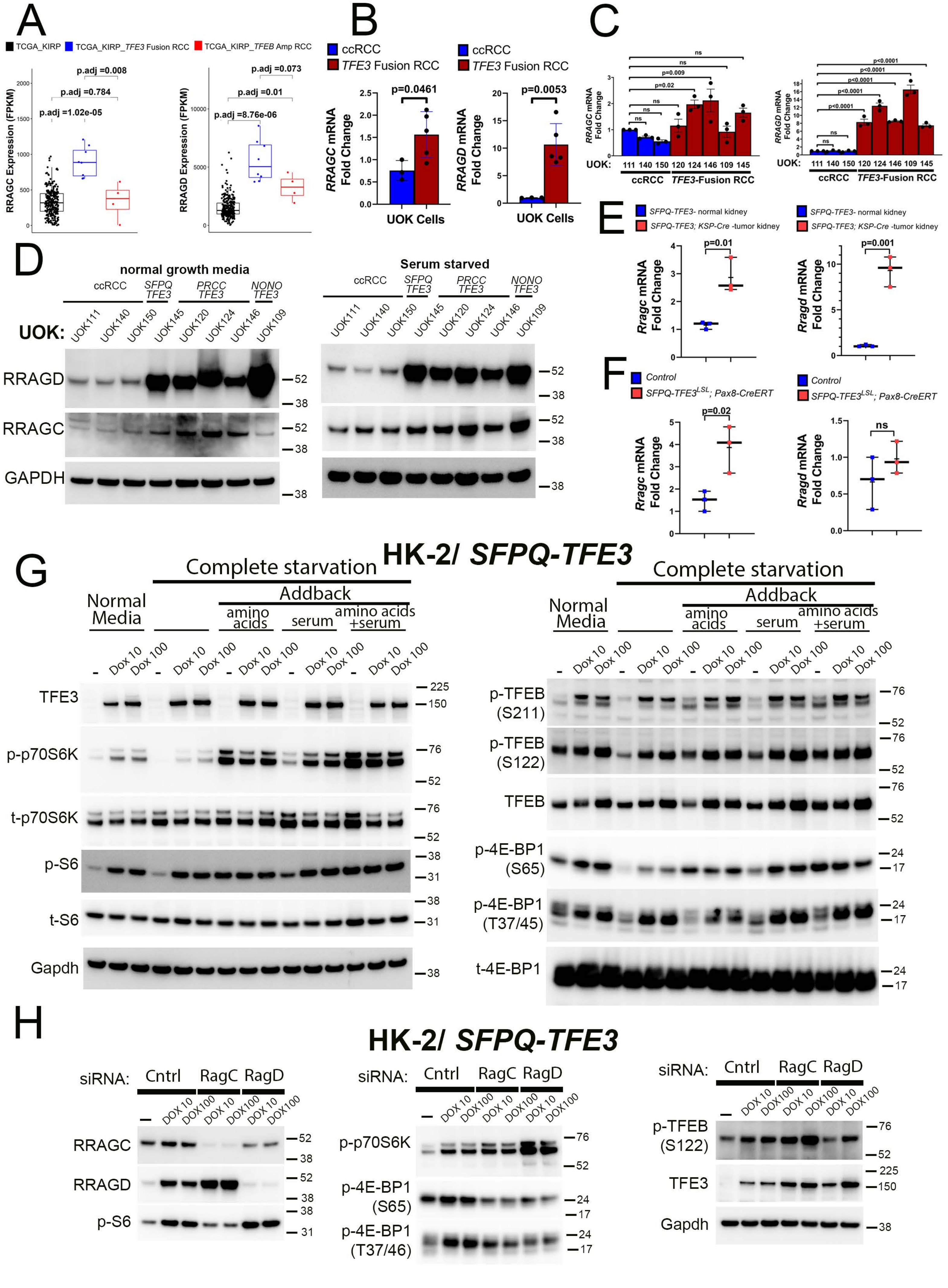

Supplementary Figure 5: ***mTORC1 signaling is activated in human models of SFPQ-TFE3 fusion-RCC.*** **(A)** Comparison of *RRAGC* and *RRAGD* gene expression in *TFE3* fusion-RCC (n=8) and *TFEB*-amplified RCC (n=4) to the remainder of papillary RCC cases without underlying *TFE3* fusions or *TFEB* amplifications (n=273) in the papillary RCC (KIRP) cohort from TCGA. *P*-values indicated are by Wilcoxon rank sum test adjusted with multiple comparisons using the false discovery rate (FDR) method. Two-sided tests were used in the analysis. Data are represented as box-and-whisker plots, where center line indicates median, box edges represent 25th and 75th percentiles, and whiskers extend to the most extreme data points within 1.5× the interquartile range (IQR). Outliers beyond this range are shown as individual points. **(B)** qRT-PCR for *RRAGC* and *RRAGD* transcripts in *TFE3*- fusion RCC cell lines and ccRCC controls (n=3 [ccRCC] and n=5 [*TFE3*- Fusion RCC]; error bars represent SEM; p-values by two-tailed, Student's T-test). **(C)** qRT-PCR for *RRAGC* and *RRAGD* transcripts across individual *TFE3*- fusion RCC cell lines and ccRCC controls (n=3; error bars represent SEM; p-values by one-way ANOVA with Dunnett's test for multiple comparisons). **(D)** Immunoblotting of lysates from *TFE3* fusion-RCC and ccRCC cell lines for RRAGC and RRAGD expression. qRT-PCR for *RRAGC* and *RRAGD* transcripts in kidney lysates from: **(E)** control and *SFPQ-TFE3*^{LSL}*; Ksp-Cre* transgenic mice at post-natal day 15 and **(F)** tamoxifen-injected, *SFPQ-TFE3*^{LSL}*; Pax8-CreERT* transgenic mice at 3.5 months following injection of tamoxifen (n=3, error bars represent SEM; p-values by two-tailed, Student's T-test). **(G)** Immunoblotting of lysates from HK2/*SFPQ-TFE3* cells in normal media, or following nutrient deprivation for 90 min, or following nutrient deprivation followed by stimulation with amino acids, serum or both for 30 min, for the indicated antibodies. **(H)** Immunoblotting of lysates from HK2/*SFPQ-TFE3* cells following treatment with control, *RRAGC* or *RRAGD* siRNA and the

indicated doses of doxycycline for the indicated antibodies. All experiments represent $n \geq 3$ independent biological replicates. Source data are provided as a Source data file.

Supplementary Figure 6

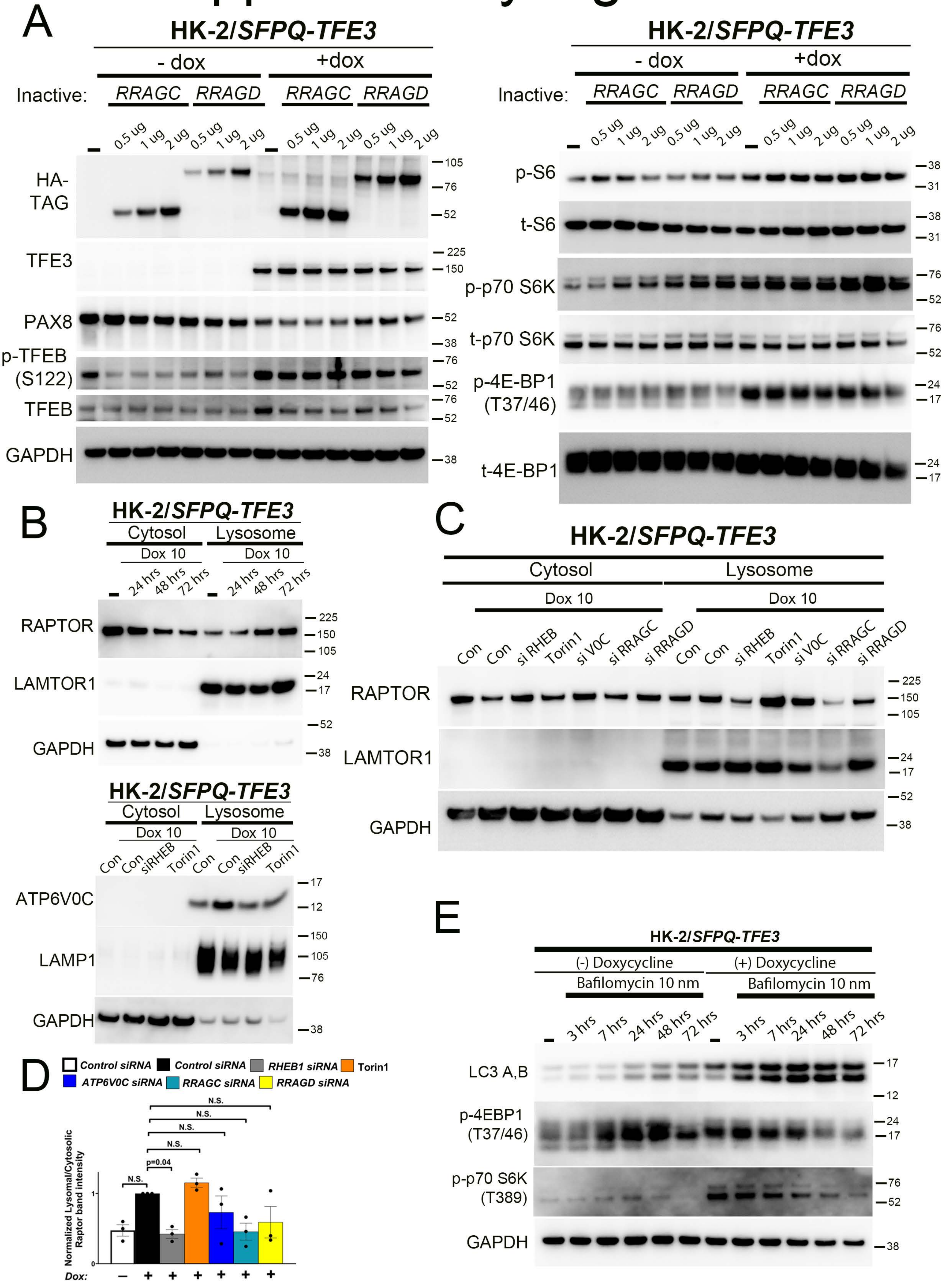

Supplementary Figure 6: ***mTORC1 signaling is activated in human models of SFPQ-TFE3 fusion-RCC.*** **(A)** Immunoblotting of lysates from HK2/*SFPQ-TFE3* cells, either untreated or treated with doxycycline for 72 hrs, following transfection of cells with the indicated concentrations of HA-tagged, inactive RRAGC^{GTP} (Q120L) or RRAGD^{GTP} (Q121L), for the indicated antibodies. **(B)** HK2/*SFPQ-TFE3* cells, were either untreated or treated with doxycycline for 24, 48 or 72 hrs (top panels), or were treated with doxycycline for 72 hrs following mTORC1 inhibition with *RHEB1* siRNA or Torin1 (bottom panels) and were subjected to lysosomal fractionation and immunoblotting of cytosolic and lysosomal fractions, for the indicated antibodies. **(C)** HK2/*SFPQ-TFE3* cells, either untreated or treated with doxycycline for 72 hrs, and following treatment with control, *RHEB1, ATP6V0C, RRAGC* or *RRAGD* siRNA, or Torin 1, were subjected to lysosomal fractionation and immunoblotting of cytosolic and lysosomal fractions, for the indicated antibodies. **(D)** Densitometry quantification of mean normalized protein expression from experiments in (C) (n=3, error bars represent SEM; p-values by one-way ANOVA with Dunnett's test for multiple comparisons). (**E)** Immunoblotting of untreated or doxycycline-treated HK2/*SFPQ-TFE3* cells*,* following treatment with a time-course of BafilomycinA1 (10 nM) for the indicated antibodies (also see **Supplementary Figure 9H**). All experiments represent n ≥ 3 independent biological replicates. Source data are provided as a Source data file.

Supplementary Figure 7

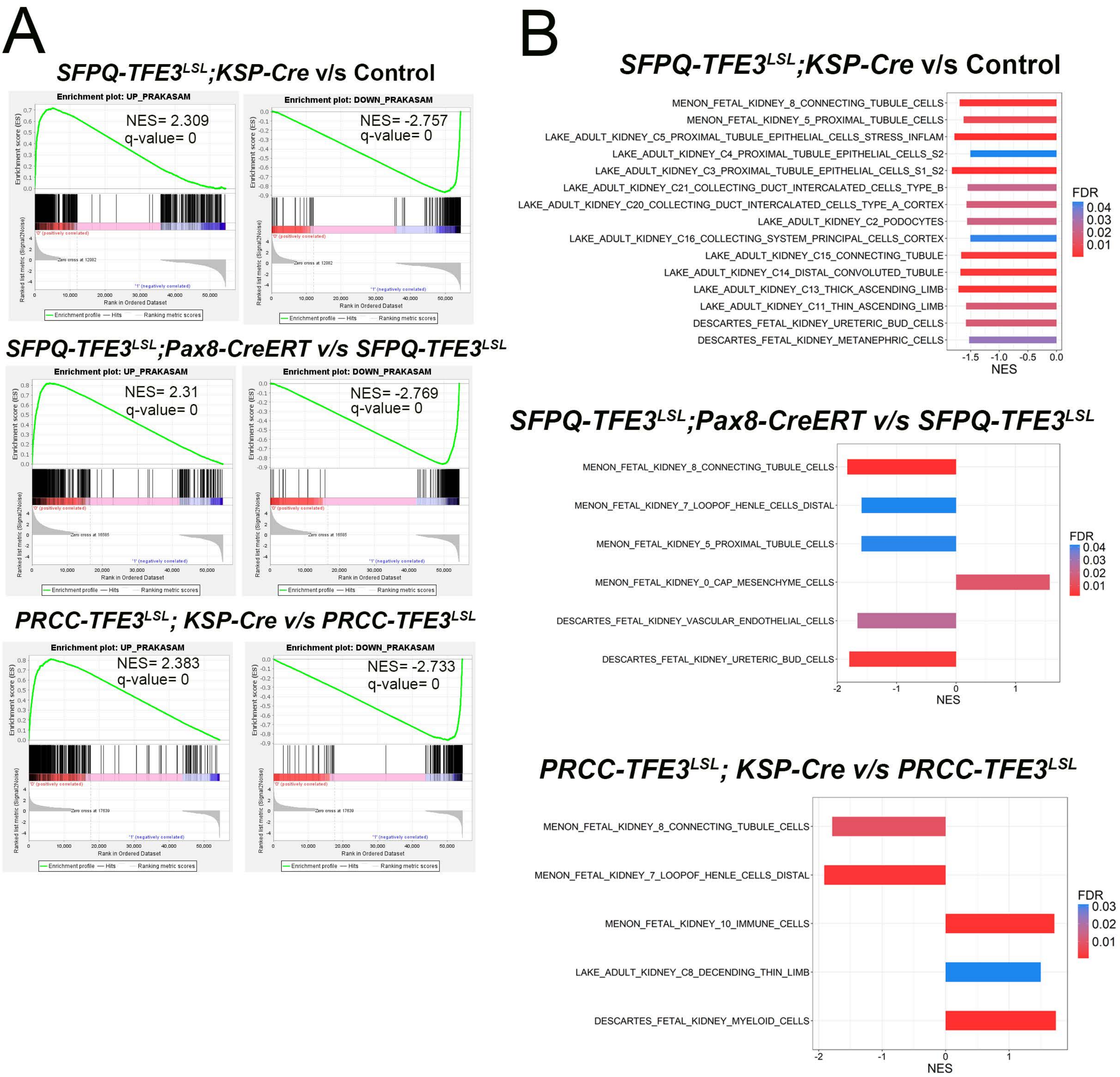

Supplementary Figure 7: ***Induction of SFPQ-TFE3 expression in human and murine renal tubular epithelial cells results in lineage plasticity with silencing of nephric lineage factors PAX8 and PAX2.*** **(A)** Gene Set Enrichment Analysis (GSEA) comparing 15-day *STK* (top panels)*,* 3.5-month tamoxifen-treated, *STP* (middle panels) and 7-month *PTK* (bottom panels), transgenic kidneys and their controls, for the core set of differentially expressed genes overlapping between human tRCC and the *ASPSCR1-TFE3* mouse model[26]. **(B)** Gene Set Enrichment Analysis (GSEA) comparing 15-day *STK* (top panels)*,* 3.5-month tamoxifen-treated, *STP* (middle panels) and 7-month *PTK* (bottom panels), transgenic kidneys and their controls, showing negative enrichment of genes associated with renal epithelial cell subsets from the cell type signature gene sets (C8). All experiments represent n ≥ 3 independent biological replicates. Source data are provided as a Source data file.

Supplementary Figure 8

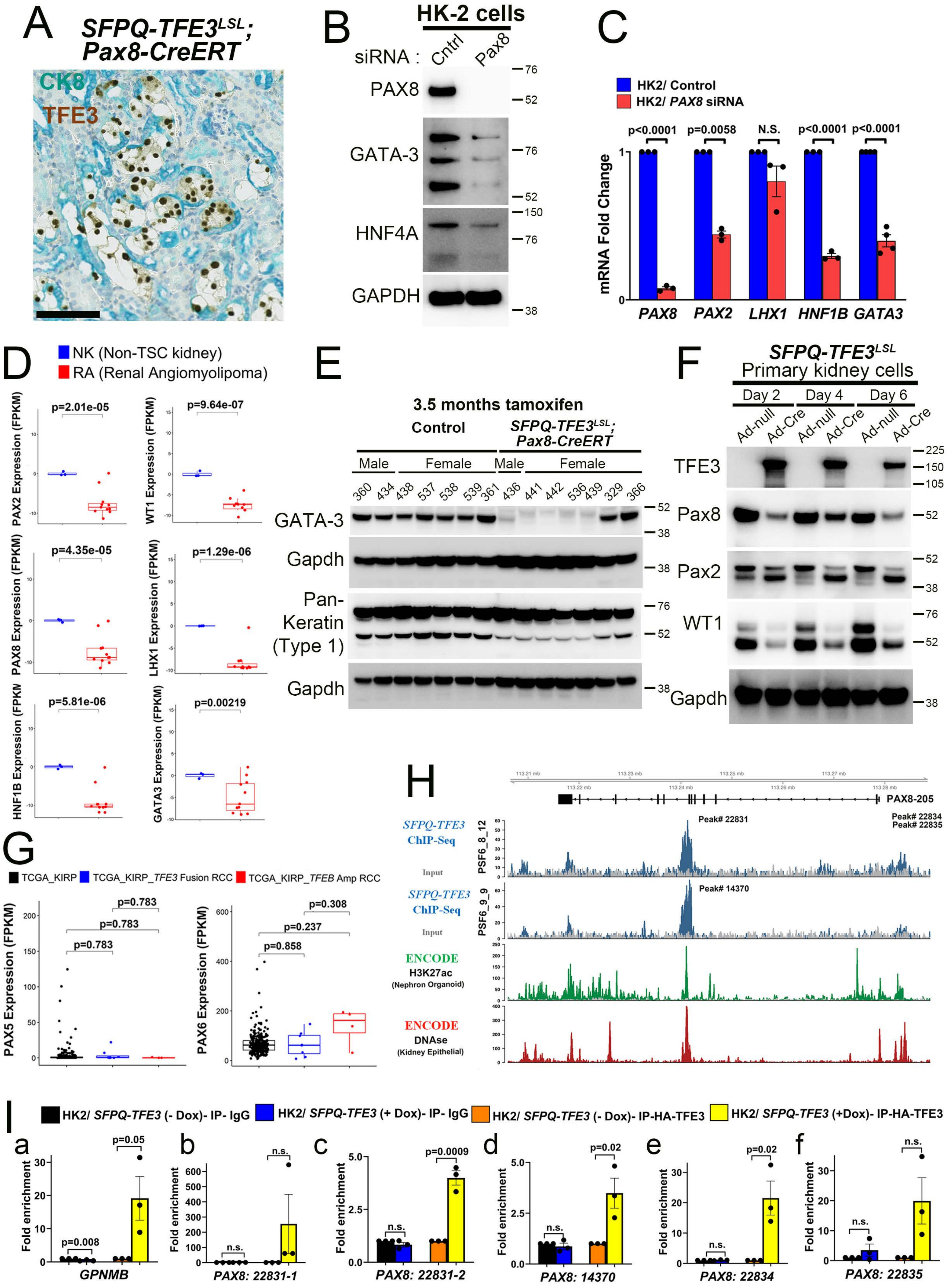

Supplementary Figure 8: ***Induction of SFPQ-TFE3 expression in human and murine renal tubular epithelial cells results in lineage plasticity with silencing of nephric lineage factors PAX8 and PAX2.*** **(A)** Dual IHC for TFE3 (brown) and CK8 (teal) in tamoxifen-injected, *STP* mice and controls, at 2 weeks following tamoxifen. Scale bar = 100 µm. **(B)** Immunoblotting and **(C)** qRT-PCR in HK2 cells following treatment with control/*PAX8* siRNA for 48 hrs. For (C), n=3 (PAX8, PAX2, LHX1 and HNF1B) and n=4 (GATA3). Graphs represent mean values; error bars represent SEM; p values by two-tailed, Student's T-test. **(D)** Comparison of *PAX2, PAX8, HNF1B, WT1, LHX1* and *GATA3* expression (FPKM) in RNA seq data from a panel of non-TSC normal kidneys (n=3) and renal angiomyolipomas with *TSC1/2* biallelic loss (n=11) [36]. *P*-values indicated are by Wilcoxon rank sum test. **(E)** Immunoblotting of kidney lysates from tamoxifen-injected, control and *STP* mice at 3.5 months following tamoxifen. **(F)** Immunoblotting of lysates of primary renal cells from *SFPQ-TFE3*^{LSL} transgenic mice treated with control or Cre-recombinase expressing adenovirus *in vitro*. **(G)** Comparison of *PAX5* and *PAX6* gene expression in *TFE3* fusion-RCC (n=8) and *TFEB*-amplified RCC (n=4) to the remainder of papillary RCC cases (n=273) in the TCGA papillary RCC (KIRP) cohort. *P*-values indicated are by Wilcoxon rank sum test adjusted with multiple comparisons using the false discovery rate (FDR) method. For **D, G**: Two-sided tests were used in the analyses. Data are represented as box-and-whisker plots, where center line indicates median, box edges represent 25th and 75th percentiles, and whiskers extend to the most extreme data points within 1.5× the interquartile range (IQR). Outliers beyond this range are shown as individual points. **(H)** Profile of *SFPQ-TFE3* ChIP-Seq binding peaks relative to the *PAX8* gene/body region, determined using an anti-HA antibody in two independent clones of doxycycline-inducible, HA-tagged, HK2/*SFPQ-TFE3* cells [PSF6_8_12 and PSF6_9_9 (blue)], with tracks for H3K27ac for Nephron organoid 21 days after differentiation from ENCODE

(green), DNAse-seq of Kidney Epithelial cells from ENCODE (red) and background/input for the ChIP-seq data/experiments (grey), indicated as shown. Y-axis represents Counts-per-million (CPM). The histone data were obtained from publicly available data from ENCODE4 project[107]. **(I)** ChIP-qPCR analyses of the *SFPQ-TFE3* binding sites within: **a)** the *GPNMB* promoter (positive control; using primers designed to amplify the M-box), **b-d)** *PAX8* intragenic region [corresponding to peaks 22831 (in PSF6_8_12) and 14370 (in PSF6_9_9) in **Supplementary Figure 8H**;] and, **e-f)** upstream of the *PAX8* TSS [corresponding to peaks 22834 and 22835 (in PSF6_8_12)] in **Supplementary Figure 8H**. Also see **Supplementary data 11-12.** ChIP-PCR was applied to amplify chromatin immunoprecipitated from the regions indicated using an anti-HA antibody or control IgG, with 2 independent sets of primers for peak 22831, in HK2/*SFPQ-TFE3* cells (untreated or treated with 10 ng/ml doxycycline for 48 hrs). (n=3, error bars represent SEM; p-values by two-tailed, Student's T-test). All experiments represent $n \geq 3$ independent biological replicates. Source data are provided as a Source data file.

Supplementary Figure 9

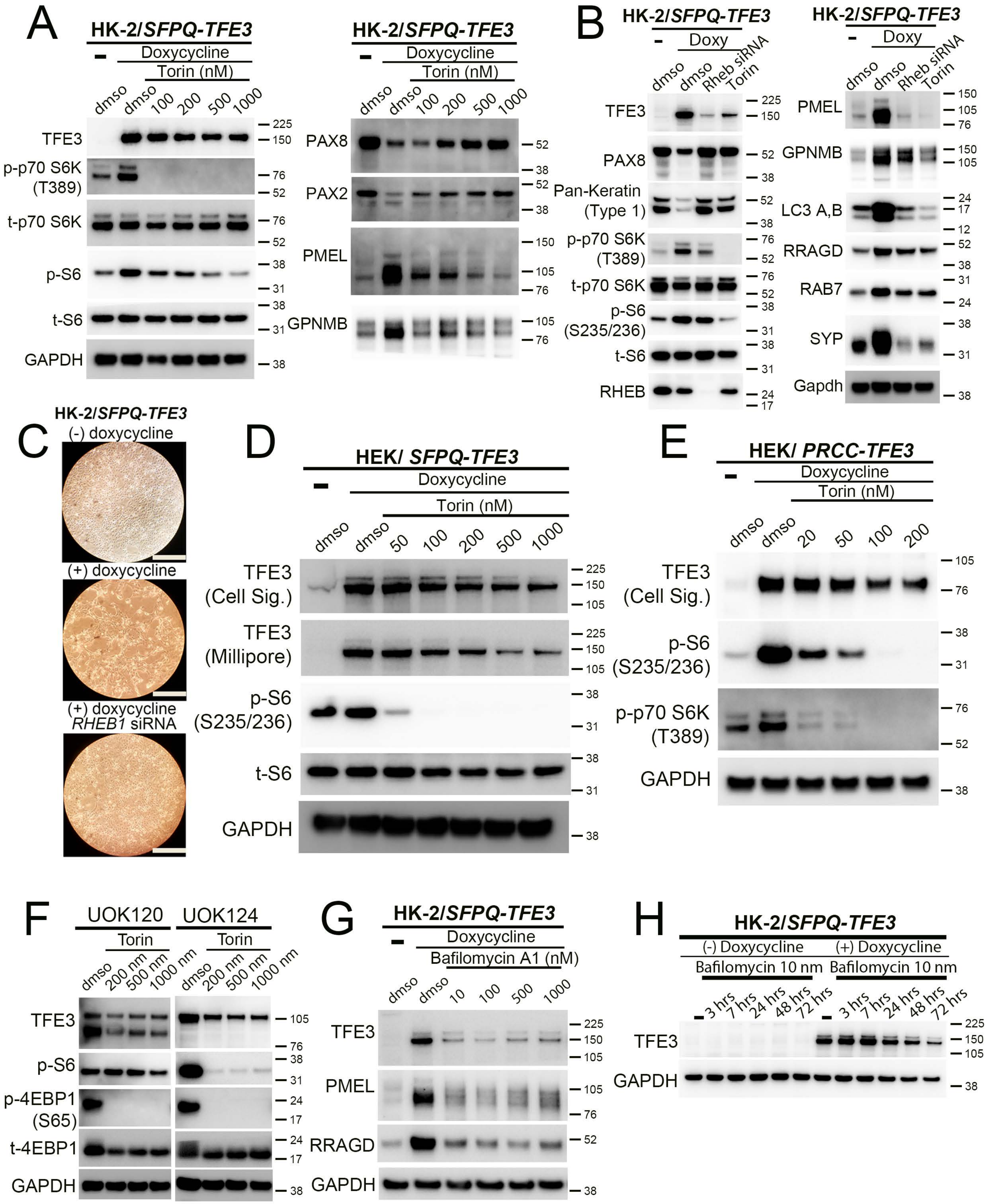

Supplementary Figure 9: *mTORC1 inhibition rescues PAX2 and PAX8 expression and activation in human vitro models of SFPQ-TFE3 expression.* **(A)** Immunoblotting of HK2 cells with doxycycline-inducible expression of *SFPQ-TFE3,* following treatment with the indicated doses of the mTOR kinase inhibitor Torin1, for the indicated antibodies. (**B)** Immunoblotting of HK2 cells with doxycycline-inducible expression of *SFPQ-TFE3,* following treatment with *RHEB1* siRNA or the mTOR kinase inhibitor Torin1, for the indicated antibodies. (**C)** Phase contrast images of HK2 cells with and without doxycycline-inducible expression of *SFPQ-TFE3,* and following treatment with *RHEB1* siRNA. Scale bar=100 µm. **(D)** Immunoblotting of lysates from HEK293 cells with doxycycline-inducible expression of *SFPQ-TFE3,* following treatment with doses of the mTOR inhibitor Torin1, for the indicated antibodies. **(E)** Immunoblotting of lysates from HEK293 cells with doxycycline-inducible expression of *PRCC-TFE3,* following treatment with doses of the mTOR inhibitor Torin1, for the indicated antibodies. **(F)** Immunoblotting of lysates from *PRCC-TFE3* cell lines (UOK120 and124), following treatment with doses of the mTOR inhibitor Torin1, for the indicated antibodies. (**G)** Immunoblotting of untreated or doxycycline-treated HK2/*SFPQ-TFE3* cells*,* following treatment with a concentration gradient of BafilomycinA1 for the indicated antibodies. (**H)** Immunoblotting of untreated or doxycycline-treated HK2/*SFPQ-TFE3* cells*,* following treatment with a time-course of BafilomycinA1 (10 nM) for the indicated antibodies. All experiments represent $n \geq 3$ independent biological replicates. Source data are provided as a Source data file.

Supplementary Figure 10

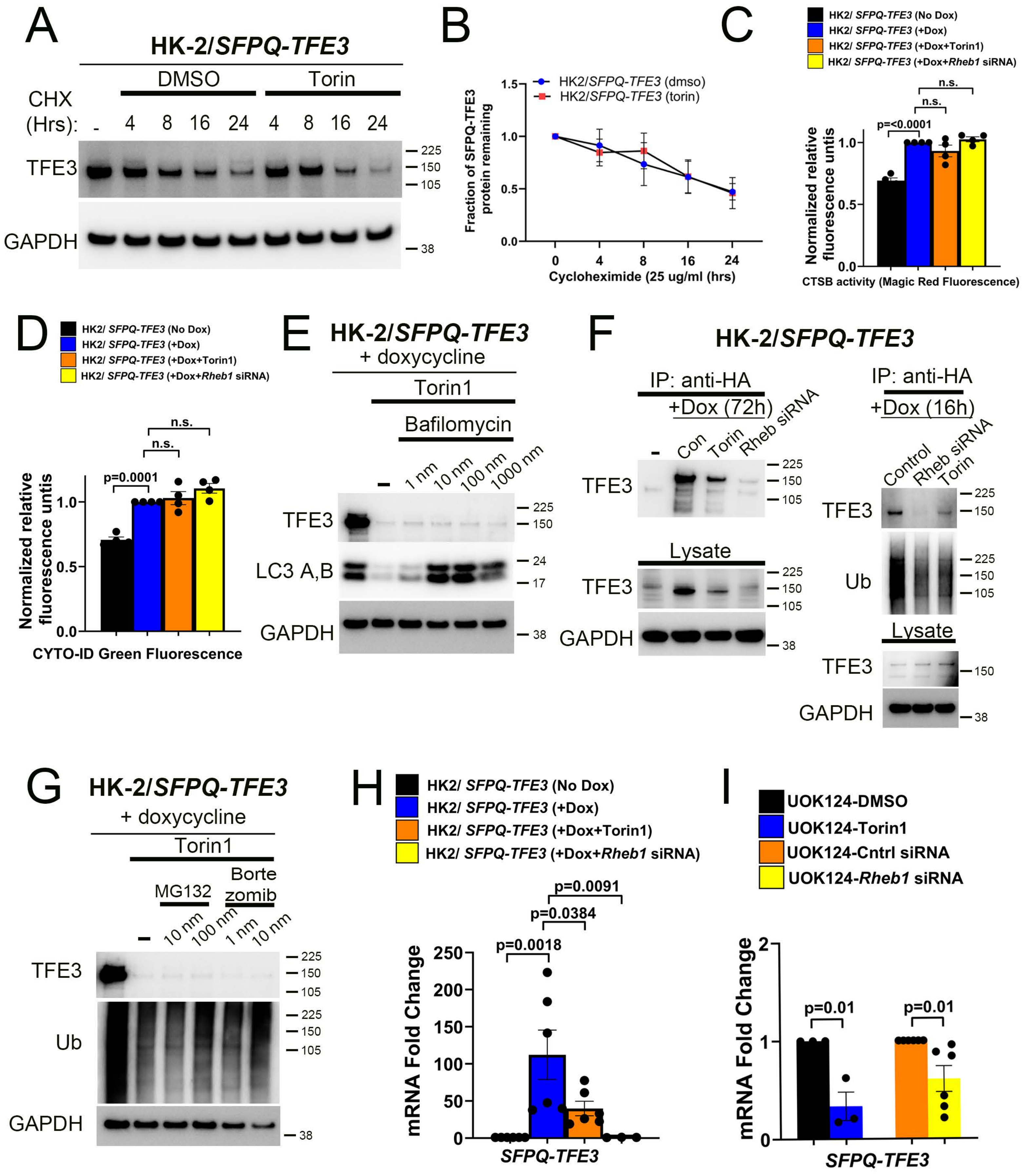

Supplementary Figure 10: ***Mechanisms by which mTORC1 inhibition downregulates SFPQ-TFE3 expression.*** **(A)** Immunoblotting of doxycycline-treated HK2/*SFPQ-TFE3* cells, treated with either dmso or Torin1 (1 uM), and following a time course of cycloheximide (25 ug/ml), for TFE3. **(B)** Densitometry quantification of normalized TFE3 protein expression at the indicated time points of cycloheximide, from experiments in (A) (n=3; graphs represent mean values; error bars represent SEM). Fluorometric analyses of Hoechst-normalized, **(C)** lysosomal cathepsin B enzyme activity using the Magic Red Cathepsin B kit and, **(D)** autophagic flux using the Cyto-ID autophagy detection kit, in untreated or doxycycline-treated HK2/*SFPQ-TFE3* cells*,* following treatment with Torin1 or *RHEB* siRNA (n=4; graphs represent mean values; error bars represent SEM; p-values by one-way ANOVA with Dunnett's test for multiple comparisons). **(E)** Immunoblotting of doxycycline-treated HK2/*SFPQ-TFE3* cells, treated with either Torin1 alone, or with increasing concentrations of BafilomycinA1 for 72 hrs, for the indicated antibodies. **(F) Left panels:** Immunoblotting of untreated or doxycycline-treated HK2/*SFPQ-TFE3* cells, treated with either Torin1or *RHEB1* siRNA, for 72 hrs. Lysates (shown in bottom panels), were immunoprecipitated with anti-HA antibody (to pull down HA-tagged, *SFPQ-TFE3*) and immunoblotted for TFE3 (top panel). **Right panels:** Immunoblotting of doxycycline-treated HK2/*SFPQ-TFE3* cells, treated with either Torin1or *RHEB1* siRNA, for 16 hrs. Lysates (shown in bottom panels), were immunoprecipitated with anti-HA antibody (to pull down HA-tagged, *SFPQ-TFE3*) and immunoblotted for Ubiquitin and TFE3 (top panels). **(G)** Immunoblotting of doxycycline-treated HK2/*SFPQ-TFE3* cells, treated with either Torin1 alone, or with increasing concentrations of MG132 or Bortezomib for 72 hrs, for the indicated antibodies. **(H)** qRT-PCR for *SFPQ-TFE3* fusion-transcripts in untreated/ doxycycline-treated HK2/ *SFPQ-TFE3* cells*,* following treatment with Torin1 or *RHEB1* siRNA. n=6 (no dox, dox, dox+Torin1) and n=3

(dox+*RHEB1* siRNA). Graphs represent mean values; error bars represent SEM; p-values by one-way ANOVA with Dunnett's test for multiple comparisons. **(I)** qRT-PCR for *PRCC-TFE3* fusion-transcripts in UOK124 cells treated with either vehicle, Torin1, control siRNA or *RHEB1* siRNA for 72 hrs. n=3 (DMSO/Torin1) and n=6 (Control/ *RHEB1* siRNA). Graphs represent mean values; error bars represent SEM; p-values by two-tailed, Student's T-test. All experiments represent $n \geq 3$ independent biological replicates. Source data are provided as a Source data file.

Supplementary Figure 11

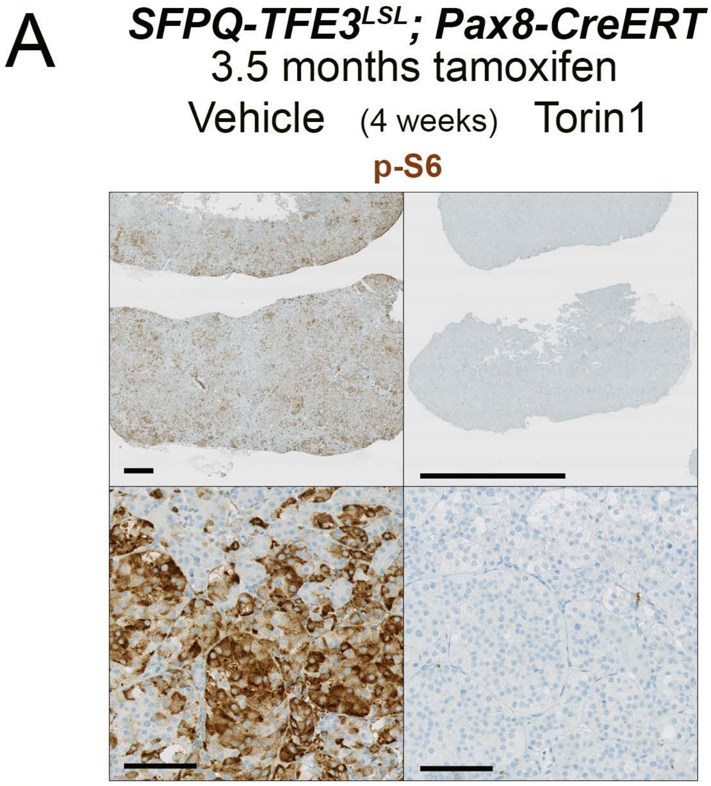

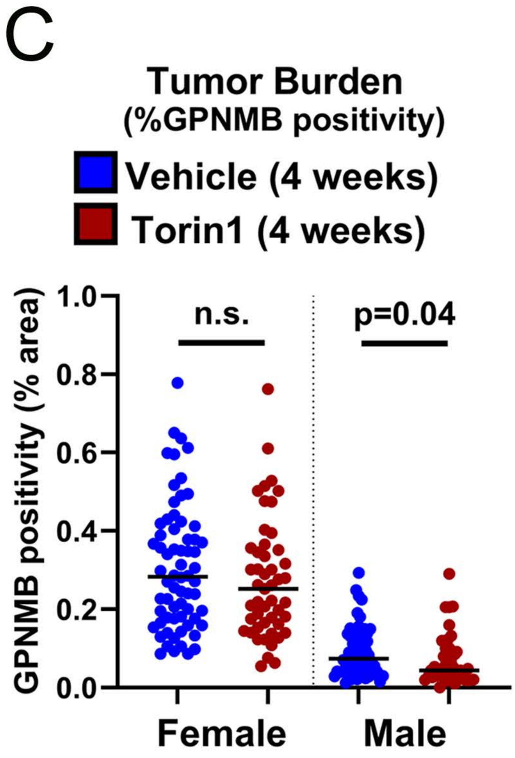

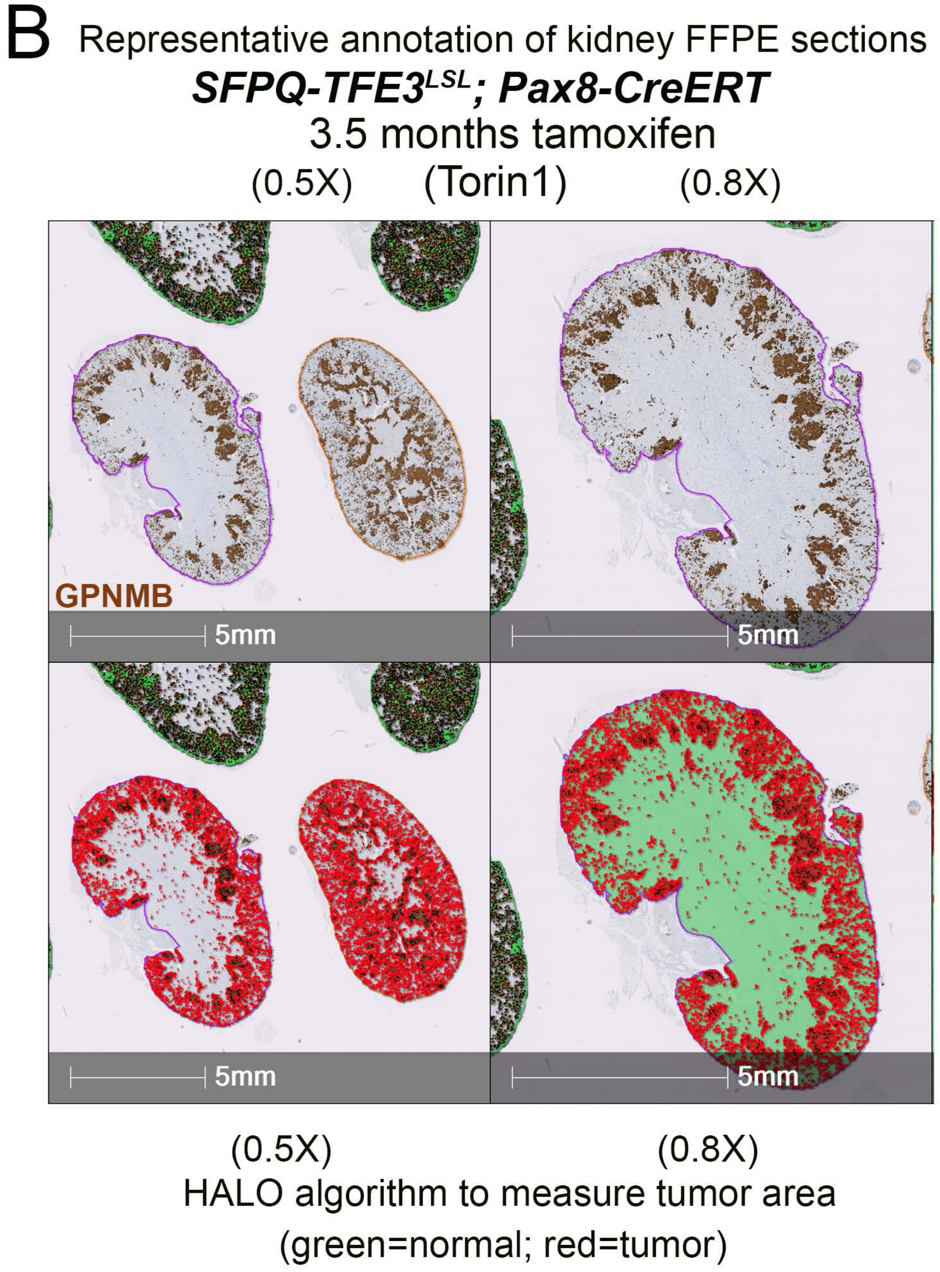

Supplementary Figure 11: ***Effects of mTOR inhibition via Torin1 treatment on kidney tumor burden in STP mice.*** **(A)** Representative IHC for p-S6 in vehicle (left panels) or Torin1-treated (right panels), tamoxifen-injected, *SFPQ-TFE3*^{LSL}*; Pax8-CreERT* transgenic mice**.** Lower panels = higher magnification images. Scale bar= 1mm (top left), 5mm (top right) and 100 µm (bottom panels). **(B)** Representative annotation of kidney FFPE section and HALO algorithm used to measure tumor area. FFPE sections of the kidneys were stained for GPNMB. Each kidney section was annotated as a region of interest (top panels), and tumor area was measured via an automated tissue classifier using HALO® trained to detect the GPNMB stain (bottom panels). The percentage of area positive for GPNMB was calculated and compared between vehicle and Torin1-treated groups for each cohort. **(C)** Estimation of kidney tumor burden in female and male *SFPQ-TFE3*^{LSL}*; Pax8-CreERT* transgenic mice, sacrificed at 3.5 months following injection of tamoxifen, and following 4 weeks of vehicle or Torin1 treatment. FFPE sections of kidneys were stained for GPNMB and the measured tumor area (assessed as % GPNMB positivity) was digitally quantified using HALO. The following numbers of ROIs were analyzed: [female/vehicle=66, female/Torin1=49, male/vehicle=60, male/Torin1=41]. Data are presented as median. P-values by two-tailed, Mann-Whitney test. Also see Supplementary Fig. 3A. All experiments represent $n \geq 3$ independent biological replicates. Source data are provided as a Source data file.
