# Supplementary material for: SFPQ-TFE3 reciprocally regulates mTORC1 and induces lineage plasticity in a mouse model of renal tumorigenesis
Source: Nat Commun. 2025 Oct 3;16:8822. doi: 10.1038/s41467-025-63885-2 (PMC12494988; doi:10.1038/s41467-025-63885-2)
Supplement: Supplementary file 2 — Description of Additional Supplementary Files.pdf [file 41467_2025_63885_MOESM2_ESM.pdf]

Description of Additional Supplementary Files

SFPQ-TFE3 reciprocally regulates mTORC1 and induces lineage plasticity in a mouse model of renal tumorigenesis

Supplementary Data 1: List of differentially expressed genes in 15-day *SFPQ-TFE3-LSL; KSP-Cre* kidneys compared to controls.

Supplementary Data 2: List of differentially expressed genes in 3.5-month tamoxifen-treated, *SFPQ-TFE3-LSL; Pax8-ERT-Cre* kidneys compared to controls.

Supplementary Data 3: List of differentially expressed genes in 7-month *PRCC-TFE3-LSL; KSP-Cre* kidneys compared to controls.

Supplementary Data 4: Gene Set Enrichment Analysis (GSEA) using standard KEGG gene sets in 15-day *SFPQ-TFE3-LSL; KSP-Cre* kidneys compared to controls.

Supplementary Data 5: Gene Set Enrichment Analysis (GSEA) using standard KEGG gene sets in 3.5-month tamoxifen-treated, *SFPQ-TFE3-LSL; Pax8-ERT-Cre* kidneys compared to controls.

Supplementary Data 6: Gene Set Enrichment Analysis (GSEA) using standard KEGG gene sets in 7-month *PRCC-TFE3-LSL; KSP-Cre* kidneys compared to controls.

Supplementary Data 7: HOMER Motif analyses showing enrichment of known consensus motifs from *SFPQ-TFE3* chromatin immunoprecipitation sequencing (ChIP-seq) experiments performed on doxycycline-inducible, HA-tagged, HK2/*SFPQ-TFE3* cells [clone 6_8_12 vs Input]. Also see Supplementary Figure 8 H, I.

Supplementary Data 8: HOMER Motif analyses showing enrichment of *de novo* consensus motifs from *SFPQ-TFE3* chromatin immunoprecipitation sequencing (ChIP-seq) experiments performed on doxycycline-inducible, HA-tagged, HK2/*SFPQ-TFE3* cells [clone 6_8_12 vs Input]. Also see Supplementary Figure 8 H, I.

Supplementary Data 9: HOMER Motif analyses showing enrichment of known consensus motifs from *SFPQ-TFE3* chromatin immunoprecipitation sequencing (ChIP-seq) experiments performed on doxycycline-inducible, HA-tagged, HK2/*SFPQ-TFE3* cells [clone 6_9_9 vs Input]. Also see Supplementary Figure 8 H, I.

Supplementary Data 10: HOMER Motif analyses showing enrichment of *de novo* consensus motifs from *SFPQ-TFE3* chromatin immunoprecipitation sequencing (ChIP-seq) experiments performed on doxycycline-inducible, HA-tagged, HK2/*SFPQ-TFE3* cells [clone 6_9_9 vs Input]. Also see Supplementary Figure 8 H, I.

Supplementary Data 11: ChIP-seq peaks from *SFPQ-TFE3* chromatin immunoprecipitation sequencing (ChIP-seq) experiments performed on doxycycline-inducible, HA-tagged, HK2/*SFPQ-TFE3* cells [clone 6_8_12 vs Input]. Also see Supplementary Figure 8 H, I.

Supplementary Data 12: ChIP-seq peaks from *SFPQ-TFE3* chromatin immunoprecipitation sequencing (ChIP-seq) experiments performed on doxycycline-inducible, HA-tagged, HK2/*SFPQ-TFE3* cells [clone 6_9_9 vs Input]. Also see Supplementary Figure 8 H, I.

Supplementary Data 13: Differences between PEComas and RCCs with respect to TFE3-fusions, morphology, immunohistochemical markers, differential diagnoses, therapeutic options and prognosis.
