# Supplementary material for: SFPQ-TFE3 reciprocally regulates mTORC1 and induces lineage plasticity in a mouse model of renal tumorigenesis
Source: Nat Commun. 2025 Oct 3;16:8822. doi: 10.1038/s41467-025-63885-2 (PMC12494988; doi:10.1038/s41467-025-63885-2)
Supplement: Supplementary file 16 — Reporting Summary [file 41467_2025_63885_MOESM16_ESM.pdf]

nature portfolio

Corresponding author(s): Kaushal Asrani (K.A.) (kasrani1@jhmi.edu)
Tamara Lotan (T.L.L.) (tlotan1@jhmi.edu)

Last updated by author(s): Aug 15, 2025

Reporting Summary

Nature Portfolio wishes to improve the reproducibility of the work that we publish. This form provides structure for consistency and transparency in reporting. For further information on Nature Portfolio policies, see our Editorial Policies and the Editorial Policy Checklist.

Statistics

For all statistical analyses, confirm that the following items are present in the figure legend, table legend, main text, or Methods section.

n/a	Confirmed	
☐	☒	The exact sample size (*n*) for each experimental group/condition, given as a discrete number and unit of measurement
☐	☒	A statement on whether measurements were taken from distinct samples or whether the same sample was measured repeatedly
☐	☒	The statistical test(s) used AND whether they are one- or two-sided<br>*Only common tests should be described solely by name; describe more complex techniques in the Methods section.*
☒	☐	A description of all covariates tested
☐	☒	A description of any assumptions or corrections, such as tests of normality and adjustment for multiple comparisons
☐	☒	A full description of the statistical parameters including central tendency (e.g. means) or other basic estimates (e.g. regression coefficient) AND variation (e.g. standard deviation) or associated estimates of uncertainty (e.g. confidence intervals)
☐	☒	For null hypothesis testing, the test statistic (e.g. F, t, r) with confidence intervals, effect sizes, degrees of freedom and P value noted<br>*Give P values as exact values whenever suitable.*
☒	☐	For Bayesian analysis, information on the choice of priors and Markov chain Monte Carlo settings
☒	☐	For hierarchical and complex designs, identification of the appropriate level for tests and full reporting of outcomes
☒	☐	Estimates of effect sizes (e.g. Cohen's *d*, Pearson's *r*), indicating how they were calculated

Our web collection on statistics for biologists contains articles on many of the points above.

Software and code

Policy information about availability of computer code

Data collection

1) All immuno-blotting data were imaged on a chemiluminescent imaging system :
a) ChemiDoc Touch Imaging System using the ImageLab Touch Software (version 2.3.0.07) (Biorad) or,
b) Gel Capture Micro Chemi Unit and GelCapture Software (version 2.2.2.0) (FroggaBio Inc.), and quantified using Image J (version 1.52p).
2) All mRNA levels were quantified using an StepOnePlus Real-time PCR system and software (version 2.3) (Applied Biosystems).
3) All immunofluorescence images were visualized using an Olympus BX41 epifluorescence microscope using DP Controller software (version 3.2.1.276) (Olympus, Center Valley, PA) or using a Nikon W-1 spinning disk confocal microscope at the UMB-SOM Confocal Microscopy Core in Baltimore, Maryland.
4) All measurements for fluorescence microplate assays (Cathepsin B activity assay and CYTO-ID autophagy assays) were obtained using GloMax Multi Detection System with Instinct Software (version 3.1.2) (Promega).
5) All colorimetry-based protein estimations were performed using the xMark Microplate Spectrophotometer and Microplate Manager Software (version 6.3) (Biorad).
6) Immunohistochemistry (IHC) on human, murine and xenograft tissues was performed on the Ventana Discovery ULTRA (version v12.31) (Ventana/Roche, Oro Valley, AZ, USA).
7) Slides were scanned by Hamamatsu NanoZoomer S360 with NZAcquire
8) Digital quantification was performed with HALO v3.1.1076.433 (Indica Labs, Albuquerque, NM, USA).

Data analysis

For RNA (qRT-PCR and ChIP-PCR) quantification, protein quantification and fluorometric quantification of Magic Red and CYTO-ID green fluorescence, statistical significance was determined using the unpaired, two-tailed Student's t-test when comparing two experimental

groups, or with one-way ANOVA with Dunnett's or Bonferroni's correction when comparing 3 or more experimental groups. Kaplan-Meier survival analyses were performed by Log-rank (Mantel-Cox) test. Statistical analyses of kidney to body weight ratios, BUN levels, Serum creatinine levels, BrdU incorporation and positivity, Ki67 positivity and Phosphorylated-Histone H3 (pH3) positivity were performed by Mann-Whitney test. Digital quantification of median nuclear Pax8 and Pax2 H-scores in murine renal tumors and median GPNMB positivity in tumor burden estimation studies, was analyzed by two-tailed Mann-Whitney test. Gene expression data (FPKM plots) were analyzed by Wilcoxon rank sum test adjusted with multiple comparisons using the false discovery rate (FDR) method). Mean values were performed in GraphPad Prism (version 8.2.1). p-values of <0.05 were considered statistically significant. All experiments were repeated at least three times (independent biological replicates) with similar results. Additionally, all experiments were performed using multiple litters of mice and/or cellular replicates and using multiple orthogonal techniques to ensure rigor. For example: a) PAX2/PAX8 nuclear localization was confirmed by immunofluorescence (cells), IHC (mouse renal tumors) and immunoblotting of nuclear-cytoplasmic fractions.

RNA sequencing and data analysis: RNA sequencing of triplicate cell line and xenograft replicates was performed at Novogene and carried out as previously described2. Raw RNAseq counts were Fragments Per Kilobase of transcript per Million mapped reads (FPKM)-normalized for data visualization in R (v4.3.2). Raw counts were also imputed in DESeq2 in R to determine differentially expressed genes. Log2 fold-changes, p-values, and adjusted p-values (false discovery rate method, FDR) were obtained for all genes and comparisons.
Gene Set Enrichment Analysis (GSEA): Raw counts were used as input for Gene Set Enrichment Analysis (GSEA, http://www.broad.mit.edu/gsea/). We employed the curated Hallmarks pathways (https://www.gsea-msigdb.org/gsea/msigdb/human/genesets.jsp?collection=H) to identify differential regulation of pathways in our comparison groups. In addition, curated sets from published studies were used to identify significantly enriched pathways in our study groups from data deposited in: a) dbGap under accession code phs001357.v1.p1.3, b) GE0 GSE2520474, or c) GEO GSE1300725. Negative NES indicated negatively enriched pathways in our comparison group vs. control. Q-value cutoffs were set to 0.1. Pan-Cancer-normalized RNAseq data from TCGA was downloaded from TCGA Pan-Cancer publication portal (https://gdc.cancer.gov/about-data/publications/panimmune). KIRP and KIRC RNAseq data were previously normalized with the methods Fragments Per Kilobase of transcript per Million mapped reads (FPKM) and FPKM Upper Quartile (FPKM-UQ) by the TCGA research team. Gene expression data was compared using Wilcoxon rank sum tests adjusted with multiple comparisons, where applicable. GSEA analyses were conducted with raw RNAseq counts using custom pathways and standard enrichment molecular signatures database. All TCGA graphs presented in the manuscript were generated by us using TCGA data. All analyses were performed in R v4.3.1.

ChIP-Seq Analysis: The single-end 100bp read length Fastq files were checked for quality using fastqc. Reads were aligned to the human genome (hg38) reference genome with bowtie2 filtering out duplicate alignments. The ChIP-seq peaks were called using MACS2 with the input ChIP samples as controls. The annotations of the peaks were performed by homer annotatePeaks.pl. The reads per million (RPM) for each summit +/- 2.5 kb region were calculated using bedtools coverage. Intersections between peaks regions or summit +/- 2.5 kb regions were calculated by using bedtools intersect (v 2.31.0). Peak detection was performed using the MACS2 algorithm6 in the Strand NGS software (Strand Life Sciences). HA-SFPQ-TFE3 binding was identified by significant enrichment of each signal over input DNA peaks with a p-cutoff value of 10^{-5}. ChIP-seq data visualization was carried out using deepTools v3.4.17. Coverage tracks were generated with the bamCoverage tool, normalized to Reads Per Genomic Content (RPGC). The resulting tracks were visualized using Gviz8. For consistency, ENCODE4 data were processed and visualized using the same workflow, starting from publicly available alignment files. In ChIP qPCR, DNA purification was performed in the same manner as in ChIP seq.

For manuscripts utilizing custom algorithms or software that are central to the research but not yet described in published literature, software must be made available to editors and reviewers. We strongly encourage code deposition in a community repository (e.g. GitHub). See the Nature Portfolio guidelines for submitting code & software for further information.

Data

Policy information about availability of data

All manuscripts must include a data availability statement. This statement should provide the following information, where applicable:

- Accession codes, unique identifiers, or web links for publicly available datasets
- A description of any restrictions on data availability
- For clinical datasets or third party data, please ensure that the statement adheres to our policy

Data availability: All data generated and analyzed during the current study are included in this published article and its supplementary information files, or are deposited in GEO. All unique materials generated (such as the SFPQ-TFE3 LSL transgenic mice) during this study are available from the corresponding author upon reasonable request. A reporting summary for this article is available as a Supplementary information file. The RNA-seq data from this study are deposited into NCBI's Gene Expression Omnibus (GEO) database with the accession code GSE284169. The ChIP-Seq data from this study are deposited into NCBI's Gene Expression Omnibus (GEO) database with the accession code GSE297289. Source data are provided with this paper.

Research involving human participants, their data, or biological material

Policy information about studies with human participants or human data. See also policy information about sex, gender (identity/presentation), and sexual orientation and race, ethnicity and racism.

Reporting on sex and gender	NA
Reporting on race, ethnicity, or other socially relevant groupings	NA
Population characteristics	H&E of a human TFE3-rearranged PEComa This is a diagnostic H&E stained slide from a de-identified patient provided as an example of the histology of human PEComas. It is excess diagnostic material obtained under a waiver of consent and is fully de-identified. Thus, gender and age are not provided. IRB number: IRB00223370.
Recruitment	NA

Ethics oversight	Animal protocols were approved by the JHU Animal Care and Use Committee. IRB number- IRB00223370.

Note that full information on the approval of the study protocol must also be provided in the manuscript.

Field-specific reporting

Please select the one below that is the best fit for your research. If you are not sure, read the appropriate sections before making your selection.

- [x] Life sciences
- [ ] Behavioural & social sciences
- [ ] Ecological, evolutionary & environmental sciences

For a reference copy of the document with all sections, see nature.com/documents/nr-reporting-summary-flat.pdf

Life sciences study design

All studies must disclose on these points even when the disclosure is negative.

Sample size	Sample sizes for in vivo experiments (Survival analyses, BUN, Creatinine, BrdU labeling etc) tumor burden estimation and in vivo drug studies (Treatment of STP transgenic mice with mTOR inhibitor Torin1) experiments were not predetermined as experiments were exploratory in nature, and were determined by the availability of mice with the correct genotype. Multiple cohorts of mice were tested, with multiple numbers of mice (male and female) per cohort.<br><br>For all other experiments, appropriate sample sizes were determined based on the variability of the experimental assay, with a minimun n=3 for all experiments.
Data exclusions	No data were excluded from the analyses.
Replication	All experiments were replicated in three or more independent biological replicates with similar results. Additionally, all experiments were performed using multiple litters or cohorts of mice and/or cellular replicates and using multiple orthogonal techniques to ensure rigor. For example: a) SFPQ-TFE3 nuclear localization was confirmed by immunofluorescence and immunoblotting of nuclear-cytoplasmic fractions, b) increased SFPQ-TFE3 transcriptional activity was confirmed by qRT-PCR, immunoblotting and IHC of lysosomal proteins (RAG GTPases, V-ATPases and melanotic markers) c) PAX2/PAX8 nuclear localization was confirmed by immunofluorescence (cells), IHC (mouse renal tumors) and immunoblotting of nuclear-cytoplasmic fractions, d) mTOR activation was assessed in multiple inducible strains and patient derived TFE3-fusion cells, as well as three strains of fusion-TFE3 transgenic mice, e) ChIP-Seq peaks of significance were validated by ChIP-PCR, f) In vivo drug studies with Torin1 were performed in multiple cohorts of both, male and female STP transgenic mice.
Randomization	For in vivo drug studies, STP-transgenic mice were randomly assigned to vehicle or torin- treatmentment groups. For all other experiments, randomization was not relevant. We did not undertake detailed analyses of covariates.
Blinding	Due to limited number of personnel able to perform and/or analyze assays, investigators were not blinded during data collection and/or analyses. IHC and immunoblotting experiments were analyzed using automated, software-based quantification methods (HALO/ Image J), and quantification parameters were applied equally to all samples and replicates to reduce bias.

Reporting for specific materials, systems and methods

We require information from authors about some types of materials, experimental systems and methods used in many studies. Here, indicate whether each material, system or method listed is relevant to your study. If you are not sure if a list item applies to your research, read the appropriate section before selecting a response.

Materials & experimental systems

n/a	Involved in the study	
[ ]	[x]	Antibodies
[ ]	[x]	Eukaryotic cell lines
[x]	[ ]	Palaeontology and archaeology
[ ]	[x]	Animals and other organisms
[x]	[ ]	Clinical data
[x]	[ ]	Dual use research of concern
[x]	[ ]	Plants

Methods

n/a	Involved in the study	
[ ]	[x]	ChIP-seq
[x]	[ ]	Flow cytometry
[x]	[ ]	MRI-based neuroimaging

Antibodies

Antibodies used	Primary antibodies: 4E-BP1 (#9644 CST; 1:2000), CTSK (#ab19027 Abcam; 1:1000), FLCN (#3697 CST; 1:1000), GAPDH (#5174 CST; 1:1000), GATA3(#5852 CST; 1:500), GPNMB (#38313 CST; 1:1000), GPNMB (Mouse specific) (#90205 CST; 1:1000), Histone-3 (#4499 CST; 1:4000), HNF4A (#3113 CST; 1:500), Keratin 8 (#ab53280 Abcam; 1:1000), LC3 A, B (#12741 CST; 1:2000), MelanA (#ab210546 Abcam; 1:1000), PAX2 (#9666 CST; 1:500), PAX8 (#59019 CST; 1:500), PMEL (#ab137078 Abcam; 1:1000), Phospho-p70 S6 Kinase (Thr389) (#9205 CST; 1:1000), p70 S6 Kinase (#9202 CST; 1:1000), Phospho-S6 Ribosomal Protein (Ser235/236) (#4858 CST; 1:2000), Phospho-4E BP1 (Ser65) (#9451 CST; 1:2000), Phospho-4E BP1 (Thr37/46) (#2855 CST; 1:1000), p-TFEB(S122) (#86843 CST; 1:2000),

p-TFEB(S211) (#37681 CST; 1:2000), Pan-Keratin (Type1) (#83957 CST; 1:1000), RAB7 (#9367 CST; 1:1000), RHEB (#13879 CST; 1:1000), RRAGC (#9480 CST; 1:1000), RRAGD (#4470 CST; 1:1000), Synaptophysin (#5461 CST; 1:1000), S6 Ribosomal Protein (#2317 CST; 1:2000), TFE3 (#14779 CST; 1:4000), TFE3 (#ABE1400 Sigma; 1:4000), TFEB (#4240 CST; 1:2000), WT1 (#83535 CST; 1:1000), Ub (#43124 CST; 1:1000), LAMTOR1 (#8975 CST; 1:1000), RAPTOR (#2280 CST; 1:1000), ATP6V1A (#39517 CST; 1:1000), ATP6V1B1 (#sc-55544 Santa Cruz; 1:500), ATP6V0C (#PA116676 Thermo Fisher; 1:500), ATP6V1C1 (#sc-271077 Santa Cruz; 1:500), ATP6V0D1 (#sc-393322 Santa Cruz; 1:500), ATP6V0D2 (#PA598618 Thermo Fisher; 1:500), ATP6V1G1 (#sc-25333 Santa Cruz; 1:500), ATP6V1H (#sc-166227 Santa Cruz; 1:500), HA-Tag (#3724 CST; 1:1000).

IHC: TFE3 (Invitrogen PA5-54909; 1:5000), GPNMB (#90205 CST; 1:100), Melan A (#ab210546 Abcam; 1:500), PMEL (#ab137078 Abcam; 1:100), Phospho-S6 Ribosomal Protein (Ser235/236) (#4858 CST; 1:200), Phospho-4E BP1 (Thr37/46) (#2855 CST; 1:800), TFEB (#A303-673A, Bethyl; 1:1000), PAX8 (#ab191870 Abcam; 1:100), PAX2 (#ab79389 Abcam; 1:500), Pan-Keratin (#83957 CST; 1:25),CK8 (#ab53280 Abcam; 1:100), Vimentin (#5741 CST; 1:200), α-SMA (#19245 CST; 1:200), Synaptophysin (#36406 CST; 1:50), BrdU (#5292 CST; 1:200), Ki67 (#12202 CST; 1:100), p-Histone H3 (#06-570 Sigma; 1:500).

Validation

1) All antibodies used were validated by the manufacturer for the application tested (Immunoblotting, IHC or IF), in humans and mice
2) Additionally, all antibodies tested for immunoblotting detected bands at the expected and published molecular weights, as shown in the source data file.
3) Additionaly, many mTOR substrate antibodies (p-S6, p-4E-BP1, p-P70S6K, p-TFEB, were additionally validated by downregulation of immunoblotting bands following mTOR inhibition with Torin, Rheb siRNA or amino acid starvation.
4) TFE3 and TFEB antibodies used were additionally validated using human HEK293T cells and xenografts with CRISPR-Cas9 mediated genomic deletion of TFE3 and TFEB, in immunoblotting and IHC assays from our previous study (https://www.nature.com/articles/s41467-022-34617-7)
5) Antibodies to PAX8, Rheb and ATP6V0C were further validated following siRNA knockdown and immunoblotting assays.

Eukaryotic cell lines

Policy information about cell lines and Sex and Gender in Research

Cell line source(s)

UOK cell lines and HK2 cells with stable, doxycycline-inducible expression of TFE3 proteins were a kind gift of Dr. W. Marston Linehan (NCI). HEK293 cells with stable doxycycline-inducible expression of TFE3 proteins were generated using Flp Recombinase-mediated integration, using the Flp-In T-Rex Core Kit (K6500-01, Invitrogen).

Authentication

All cell lines were authenticated for expression of TFE3 fusions and TFE3 transcriptional targets (GPNMB/PMEL/CTSK/RRAGD/FLCN) using a combination of immunoblotting, IHC, IF and/or qRT-PCR.

Mycoplasma contamination

Cell lines tested negative for Mycoplasma contamination.

Commonly misidentified lines
(See ICLAC register)

NA

Animals and other research organisms

Policy information about studies involving animals; ARRIVE guidelines recommended for reporting animal research, and Sex and Gender in Research

Laboratory animals

Strains: The following strains were used:
1) SFPQ-TFE3LSL mice expressing the SFPQ-TFE3 fusion downstream of a LoxP-Stop-LoxP (LSL) cassette, were generated by Taconic Biosciences.
2) Mice hemizygous for the Ksp-Cre recombinase knockin gene (Strain Number: 012237) (The Jackson Laboratory).
3) Tamoxifen-inducible, Pax8 Cre-ERT2 mice were a kind gift of Dr. Athena Matakidou (Cancer Research, UK).
4) PRCC-TFE3LSL mice expressing the PRCC-TFE3 fusion downstream of a LoxP-Stop-LoxP (LSL) cassette, were a kind gift of Dr. W. Marston Linehan (NCI).

Wild animals

No wild animals were used

Reporting on sex

Sex as a biological variable: All studies were performed using both male and female strains of transgenic mice. We observed a higher prevalence of kidney tumor burden in female tamoxifen-treated, SFPQ-TFE3LSL; Pax8 Cre-ERT mice, consistent with studies in humans, and in previous studies. Sex disaggregated data have been provided for graphs in Figure 2C (kidney to body weight ratios) 2D (BUN levels) and S3A and S11C (tumor burden).

Field-collected samples

No field-collected samples were used

Ethics oversight

Animal protocols were approved by the JHU Animal Care and Use Committee, under the following protocols:

1)Targeting Lysosomal Biogenesis in Renal Tumors with TSC1/2 Loss (MO23M209)
2) Targeting GPNMB in renal tumors in tuberous sclerosis complex and translocation renal cell carcinoma (MO22M388)

Note that full information on the approval of the study protocol must also be provided in the manuscript.

Plants

Seed stocks

NA

Novel plant genotypes

NA

Authentication

NA

ChIP-seq

Data deposition

☒ Confirm that both raw and final processed data have been deposited in a public database such as GEO.

☒ Confirm that you have deposited or provided access to graph files (e.g. BED files) for the called peaks.

Data access links
May remain private before publication.

GEO Tokens for reviewer access:
ChIP-Seq:
GSE297289: utetqukuhrkxhyt
https://www.ncbi.nlm.nih.gov/geo/query/acc.cgi?acc=GSE297289

RNA-Seq
GSE284169: cjcjuccmlpyrbet
https://www.ncbi.nlm.nih.gov/geo/query/acc.cgi?acc=GSE284169

Files in database submission

Accession Title Release date Status Supplemen-tary files

GSE297289 HA-PSF-TFE3 bound regions in HA-PSF-TFE3 inducible HK-2 cells

GSM8987850 HA-PSF-TFE3_8_ChIP-seq Jul 31, 2026 approved BED

GSM8987851 HA-PSF-TFE3_9_ChIP-seq Jul 31, 2026 approved BED

GSM8987852 input DNA, control Jul 31, 2026 approved None

Genome browser session
(e.g. UCSC)

https://genome.ucsc.edu/cgi-bin/hgTracks?db=hg19&lastVirtModeType=default&lastVirtModeExtraState=&virtModeType=default&virtMode=0&nonVirtPosition=&position=chr7%3A155592223%2D155605565&hgsid=2608767508_Etz9uLgwAkD5b89BRLmTDOJ5Bvi3

Methodology

Replicates

Two biological replicates

Sequencing depth

Sample PSF_8: Total reads = 23,006,020; uniquely mapped reads = 21,165,001 (91.99%); read length = 75 bp; single-end. Sample PSF_9: Total reads = 20,832,772; uniquely mapped reads = 19,249,752 (92.40%); read length = 75 bp; single-end.

Antibodies

Anti-HA High Affinity, 3F10, Roche

Peak calling parameters

Peak calling was performed using MACS2 with default parameters: --format = AUTO, --gsize = hs, --qvalue = 0.05, --nomodel = False, --keep-dup = 1, --call-summits = False. ChIP and input control BAM files were included.

Data quality	Default MACS2 quality control was used. Peaks were filtered using FDR < 5% and >5-fold enrichment. Additional quality assessment steps (e.g., duplicate removal, mapped read filtering) were implemented using samtools and picard.
Software	The pipeline was implemented via custom shell script (ChIP-seq-SE-bwa_hg19.sh) using samtools, picard, and MACS2. The code is publicly available at:https://github.com/satoulab/PSF6_ChIP under GPL-3.0 license.
