# Supplementary material for: SFPQ-TFE3 reciprocally regulates mTORC1 and induces lineage plasticity in a mouse model of renal tumorigenesis
Source: Nat Commun. 2025 Oct 3;16:8822. doi: 10.1038/s41467-025-63885-2 (PMC12494988; doi:10.1038/s41467-025-63885-2)

Uncropped and unprocessed gels

(Main Figures)

FIGURE 1L

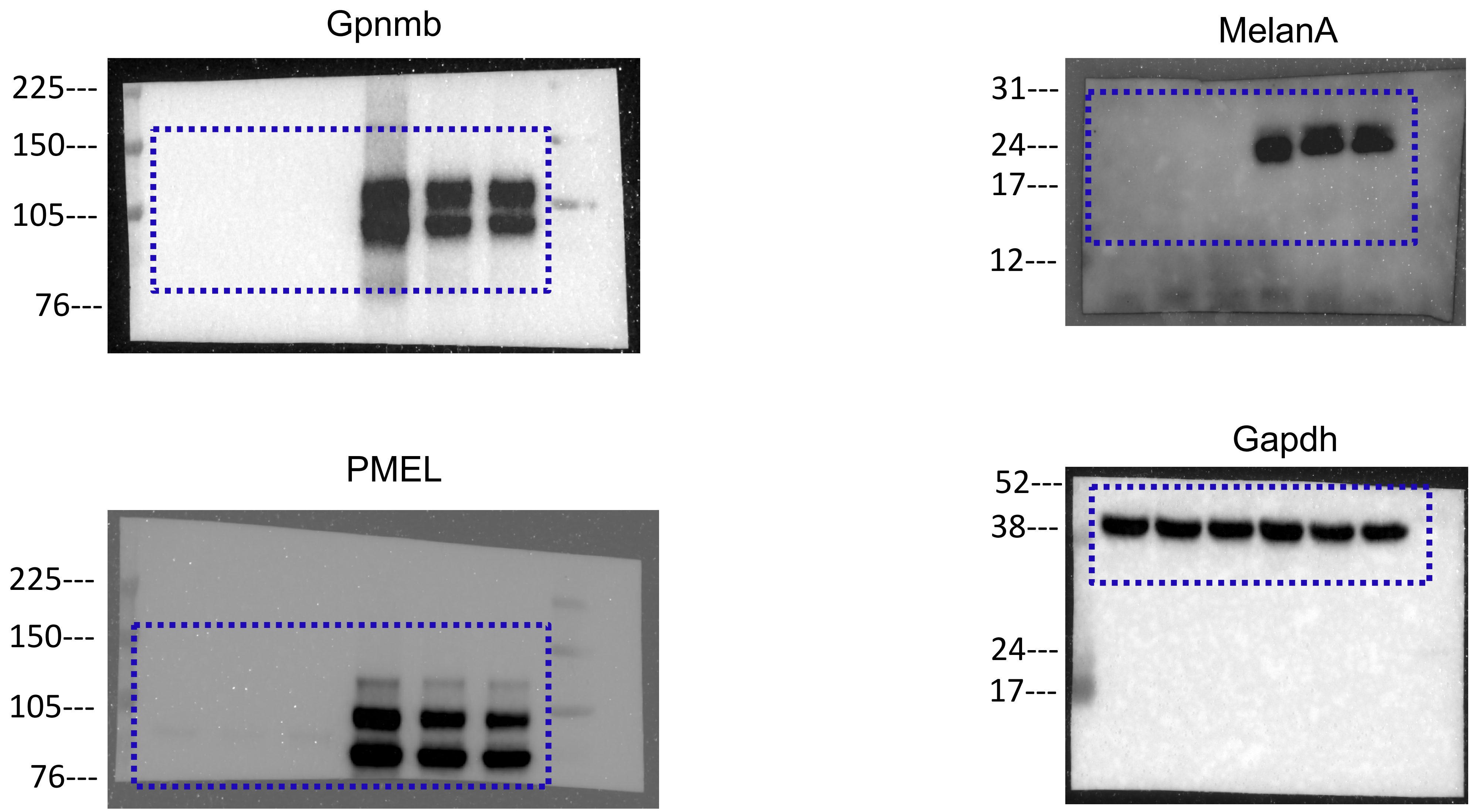
Gpnmb
225---
150---
105---
76---
MelanA
31---
24---
17---
12---
PMEL
225---
150---
105---
76---
Gapdh
52---
38---
24---
17---

FIGURE 2G

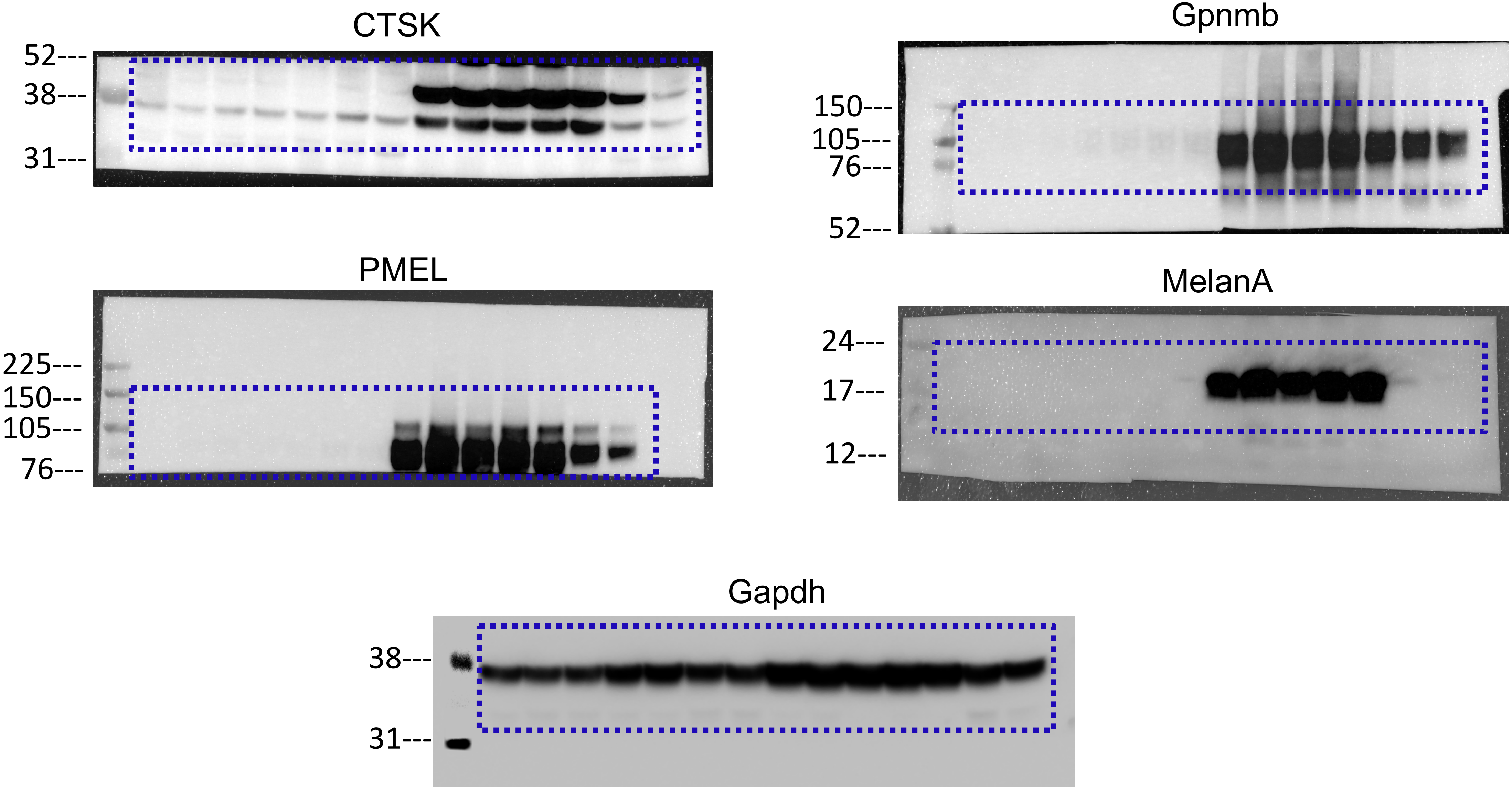
CTSK
52---
38---
31---
Gpnmb
150---
105---
76---
52---
PMEL
225---
150---
105---
76---
MelanA
24---
17---
12---
Gapdh
38---
31---

FIGURE 3A

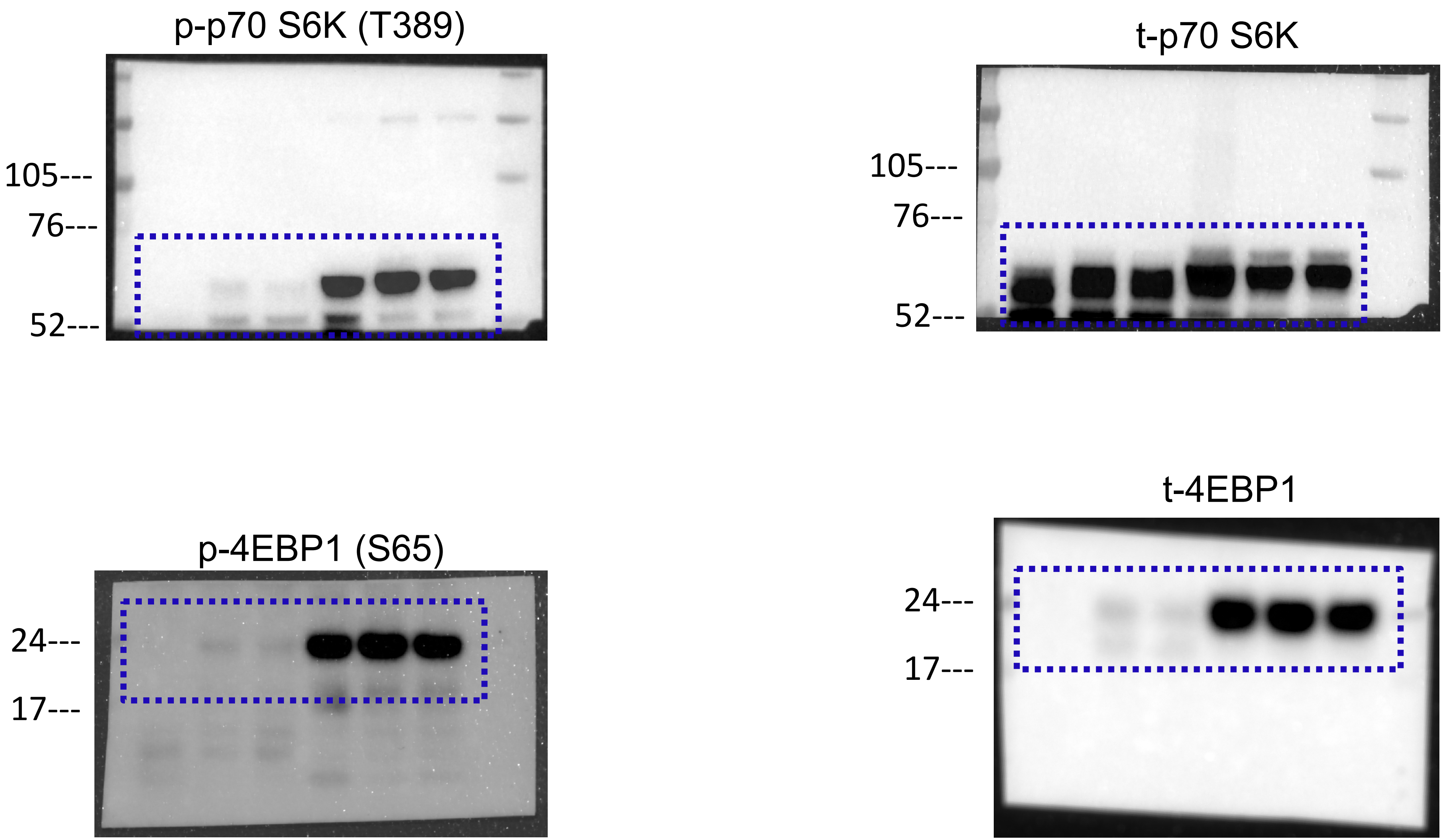
p-p70 S6K (T389)
105---
76---
52---
t-p70 S6K
105---
76---
52---
p-4EBP1 (S65)
24---
17---
t-4EBP1
24---
17---

FIGURE 3A

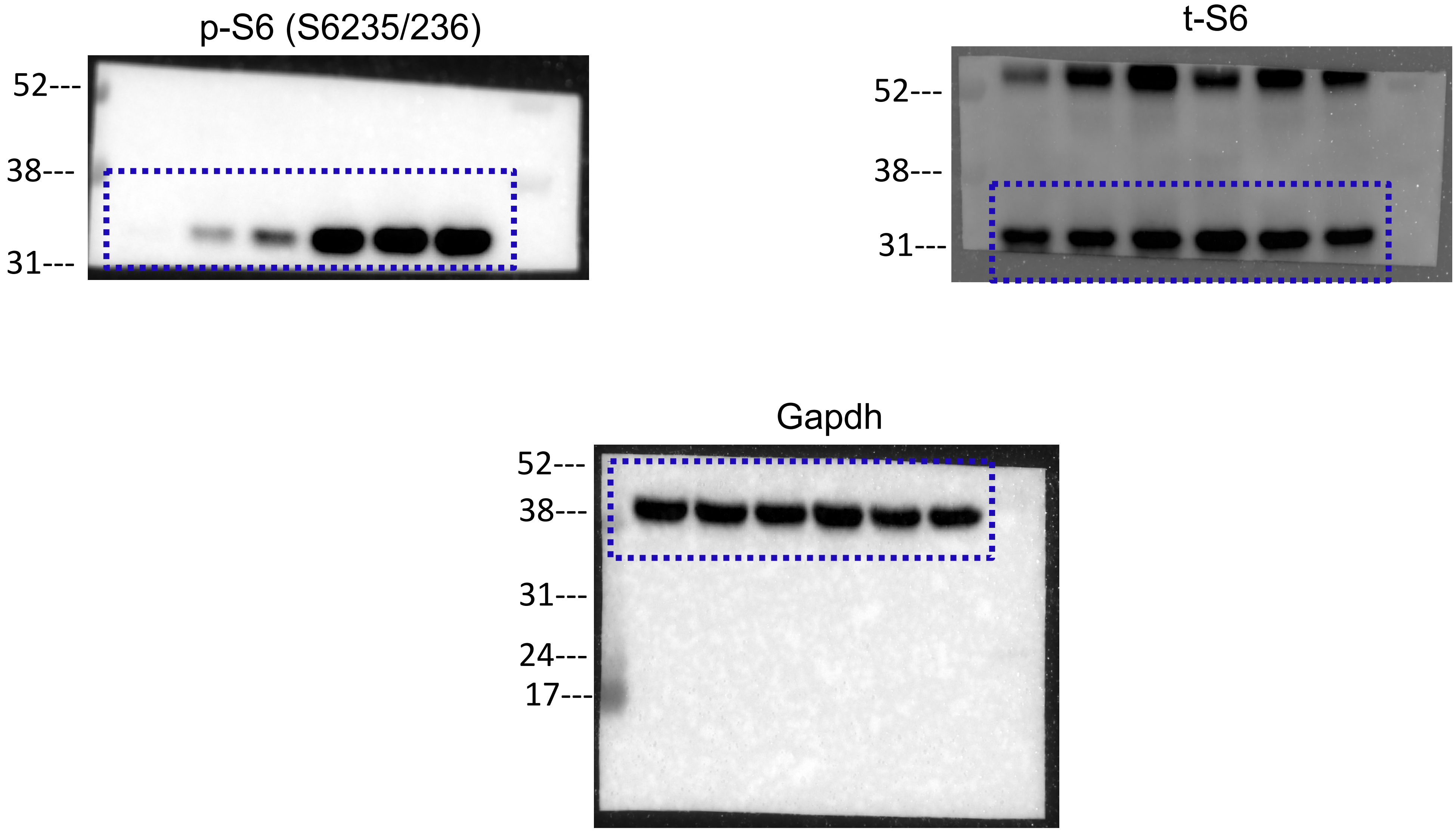

FIGURE 3C

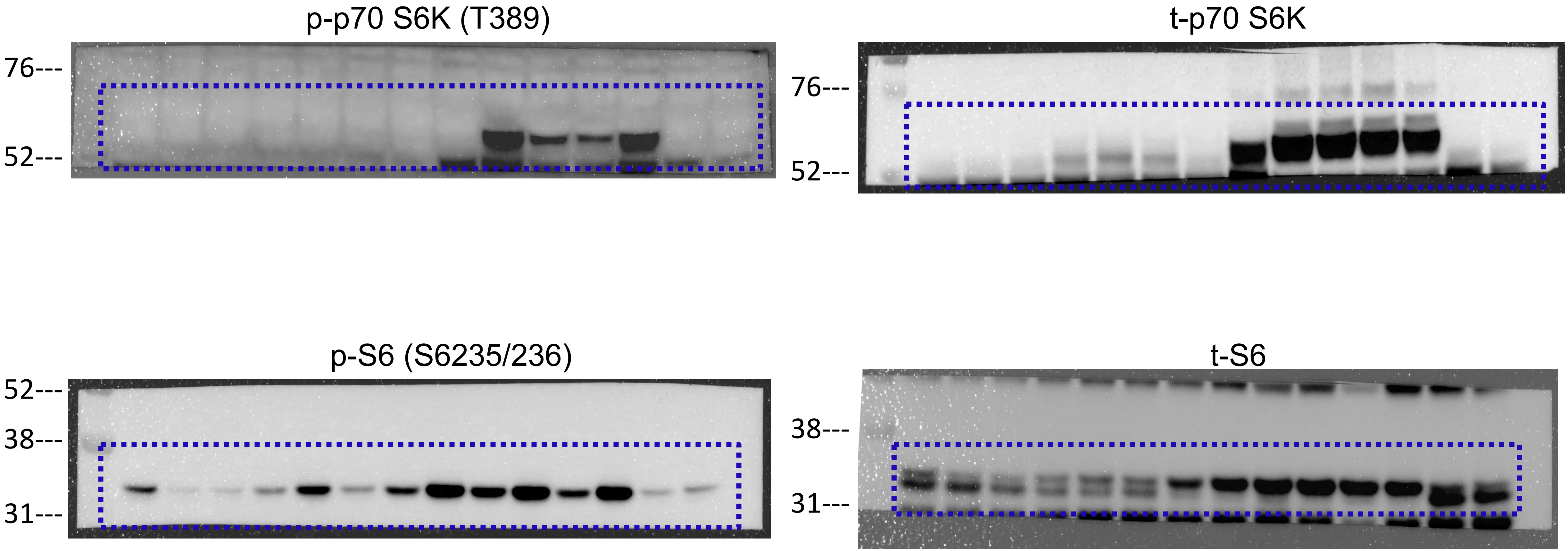

p-p70 S6K (T389)
76---
52---
t-p70 S6K
76---
52---
p-S6 (S6235/236)
52---
38---
31---
t-S6
38---
31---

FIGURE 3C

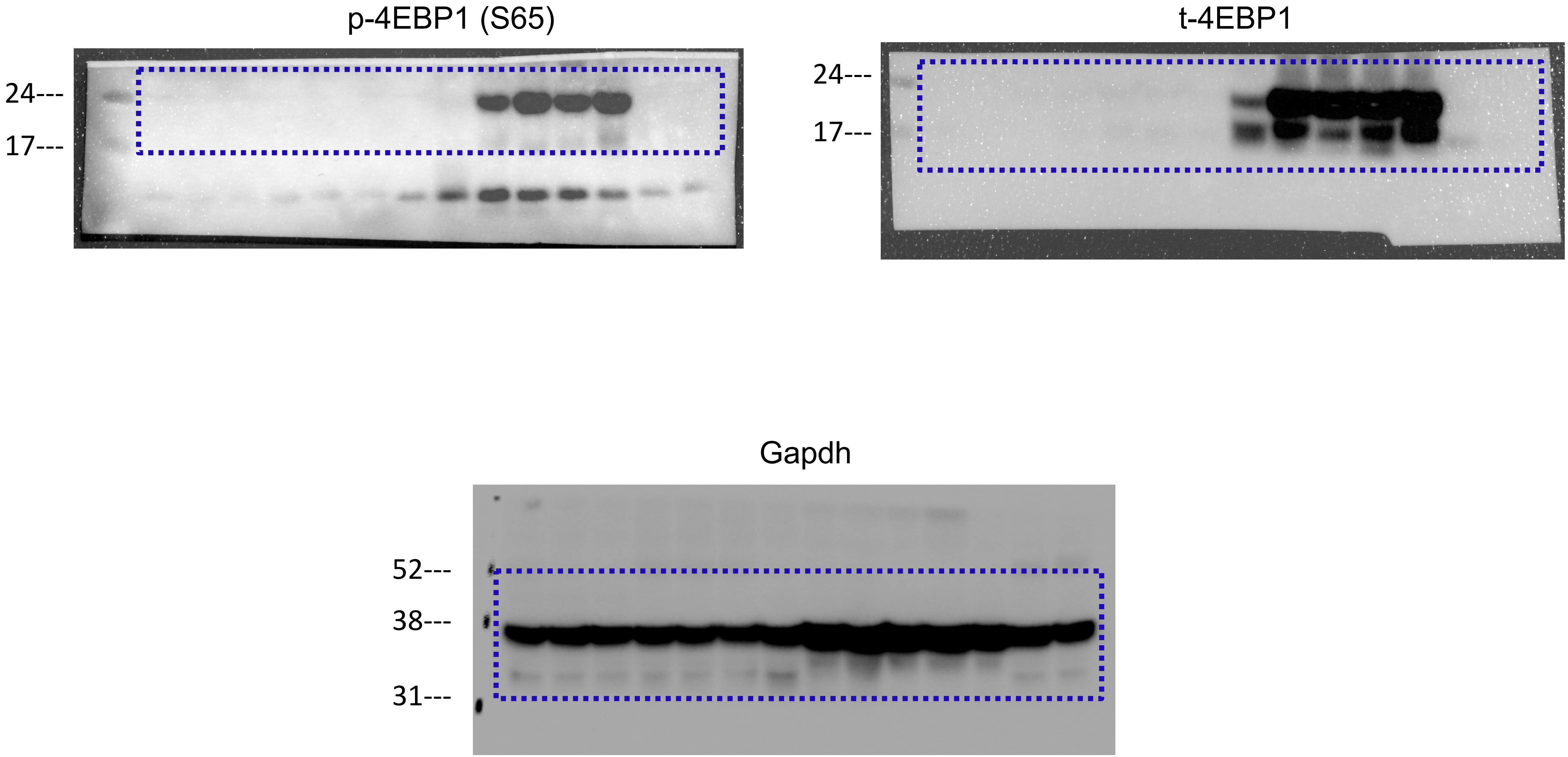

FIGURE 3F

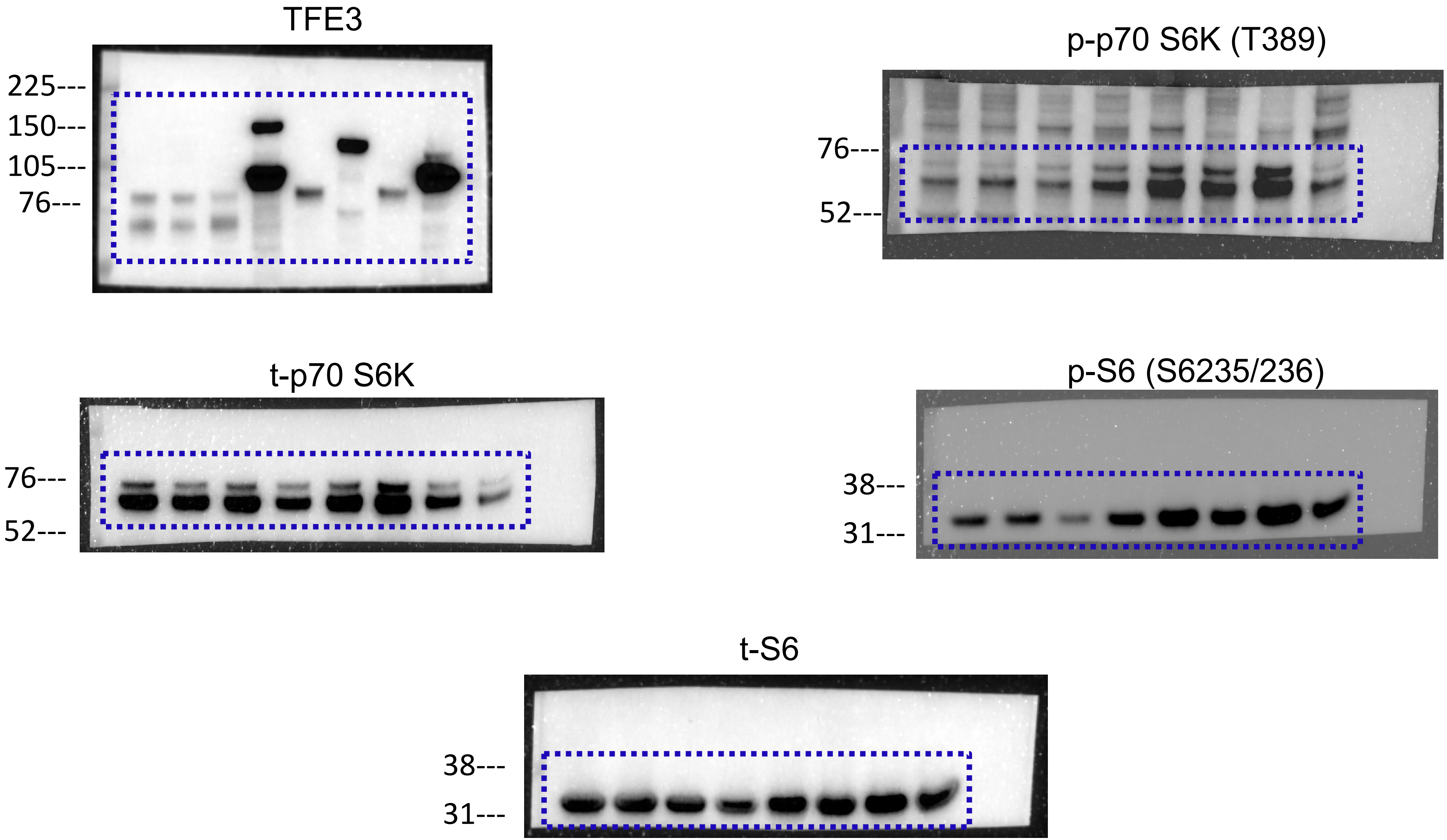
TFE3
225---
150---
105---
76---
p-p70 S6K (T389)
76---
52---
t-p70 S6K
76---
52---
p-S6 (S6235/236)
38---
31---
t-S6
38---
31---

FIGURE 3F

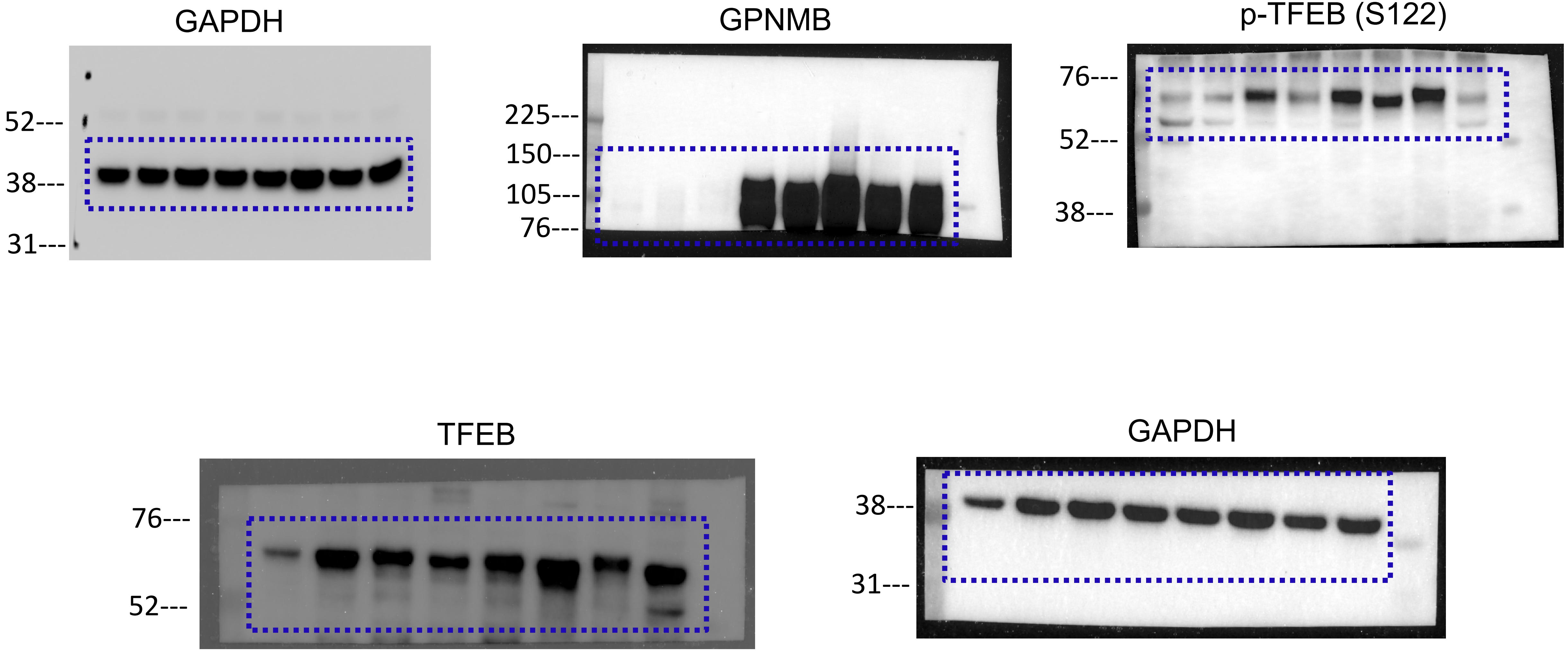
GAPDH
52---
38---
31---
GPNMB
225---
150---
105---
76---
p-TFEB (S122)
76---
52---
38---
TFEB
76---
52---
GAPDH
38---
31---

FIGURE 4C (Replicate 1 for quants in 4D)

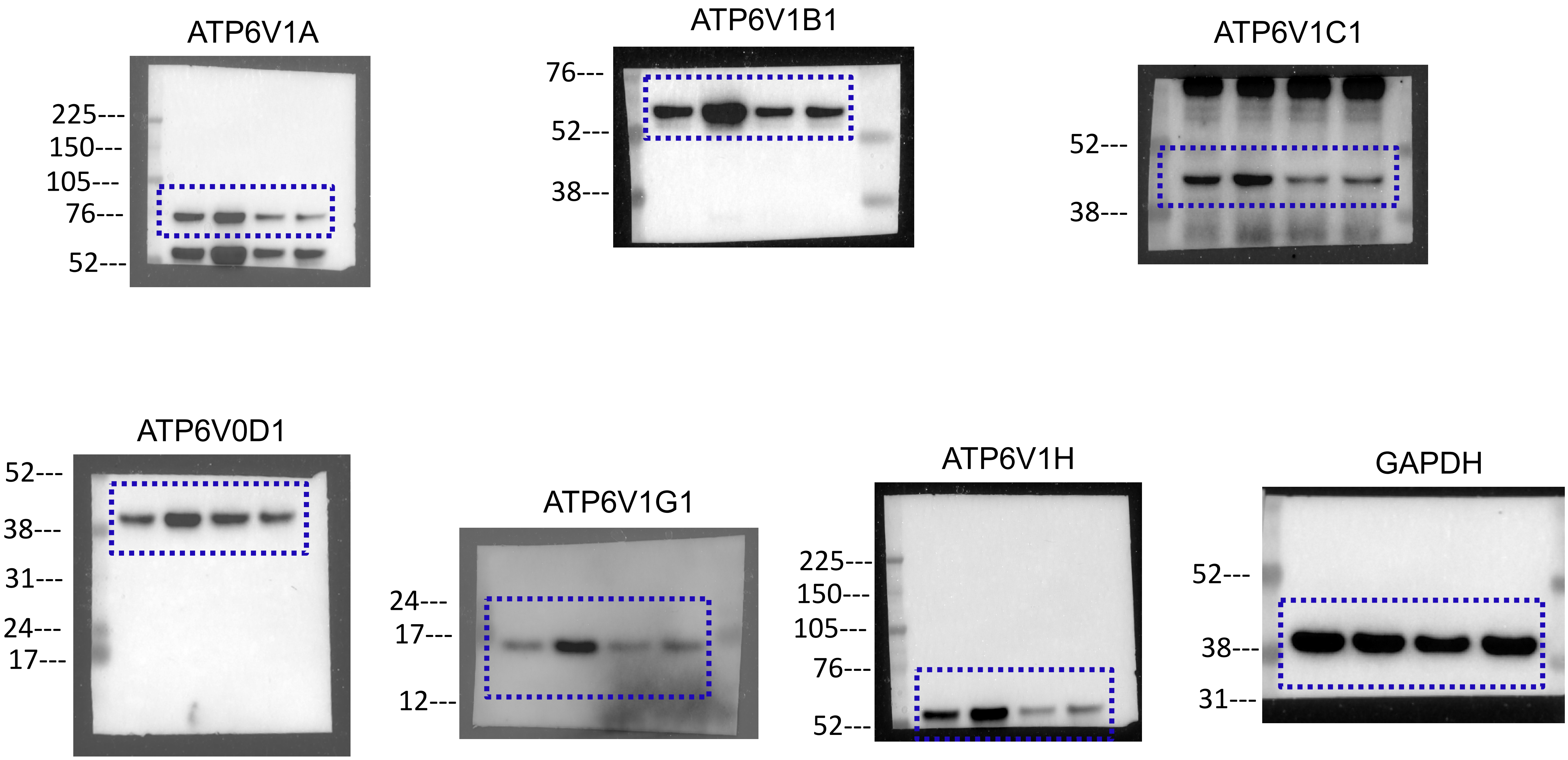

FIGURE 4C

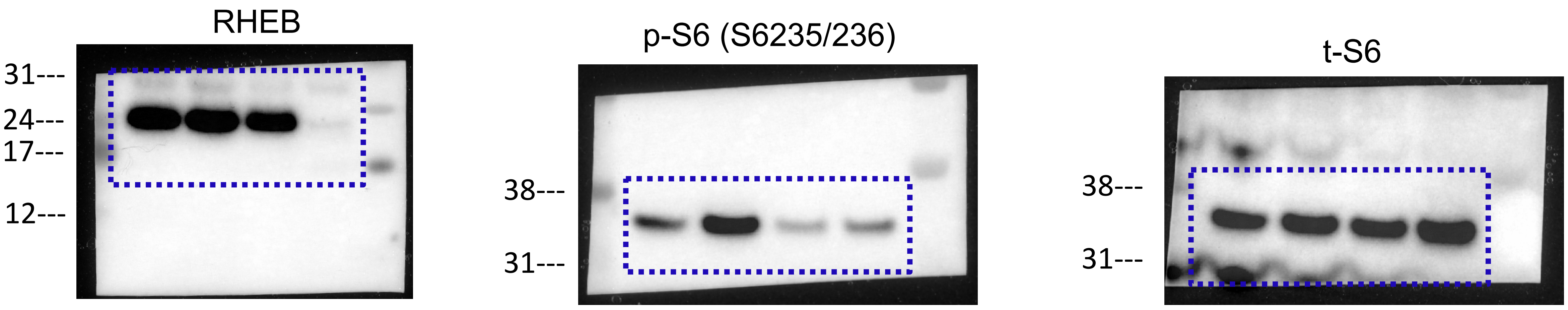

Replicate 2 for quants in 4D

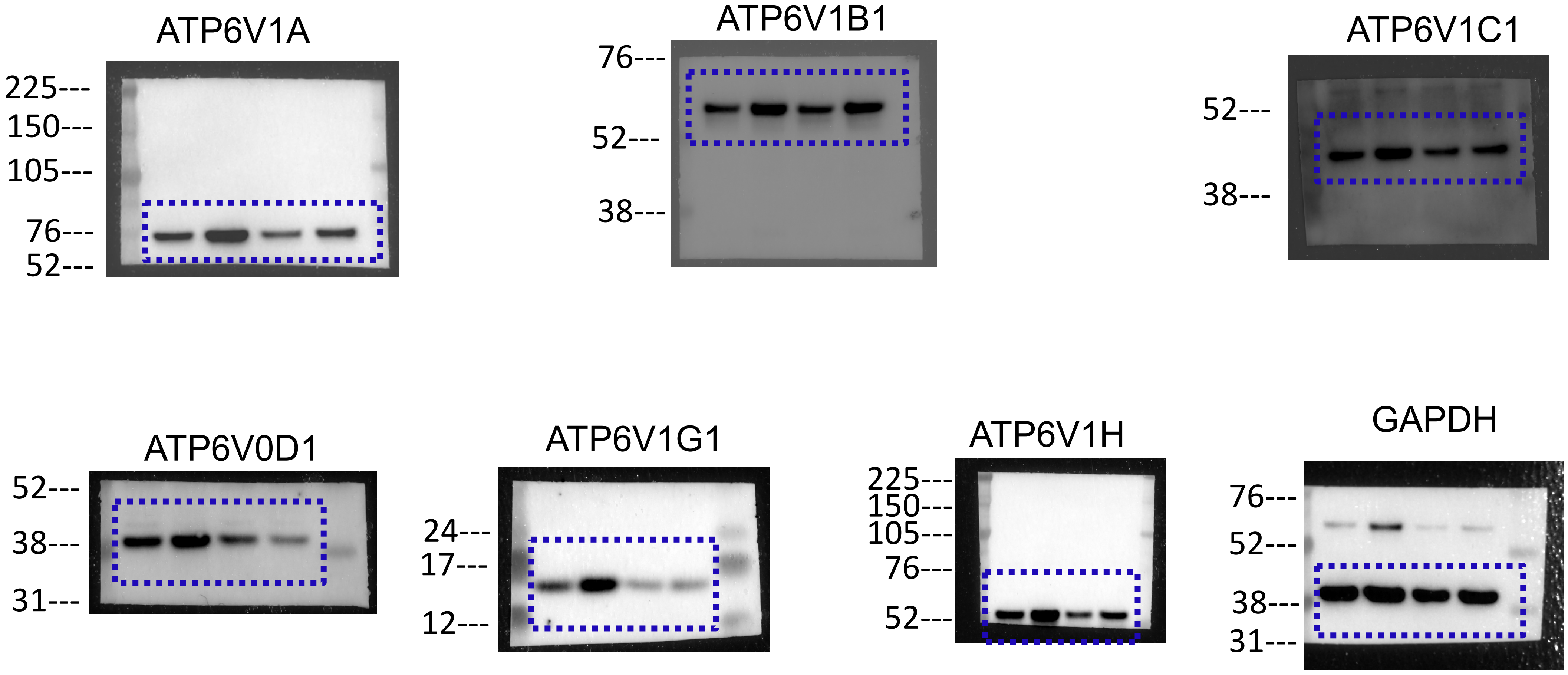

Replicate 3 for quants in 4D

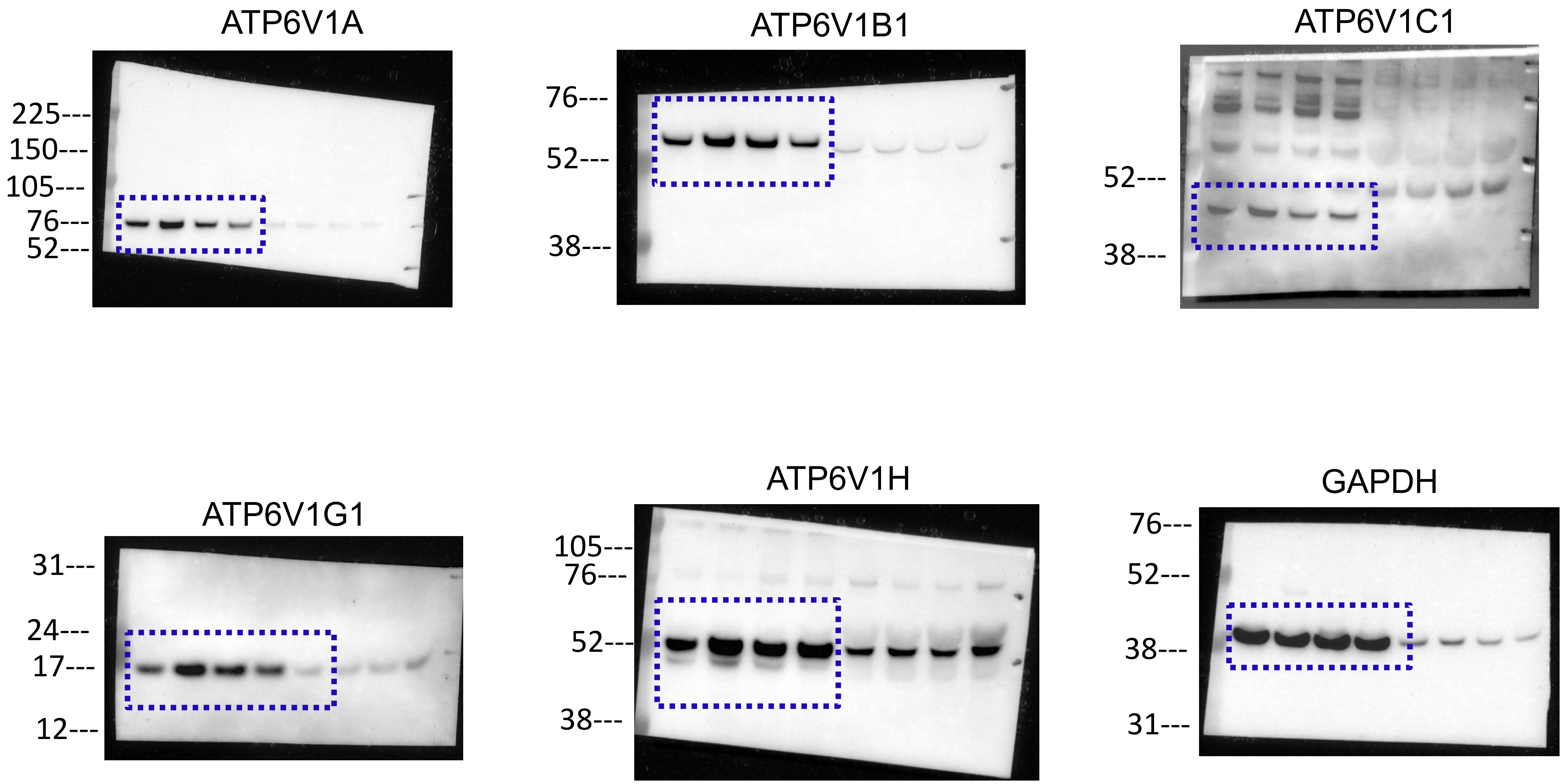

Replicate 4 for quants in 4D

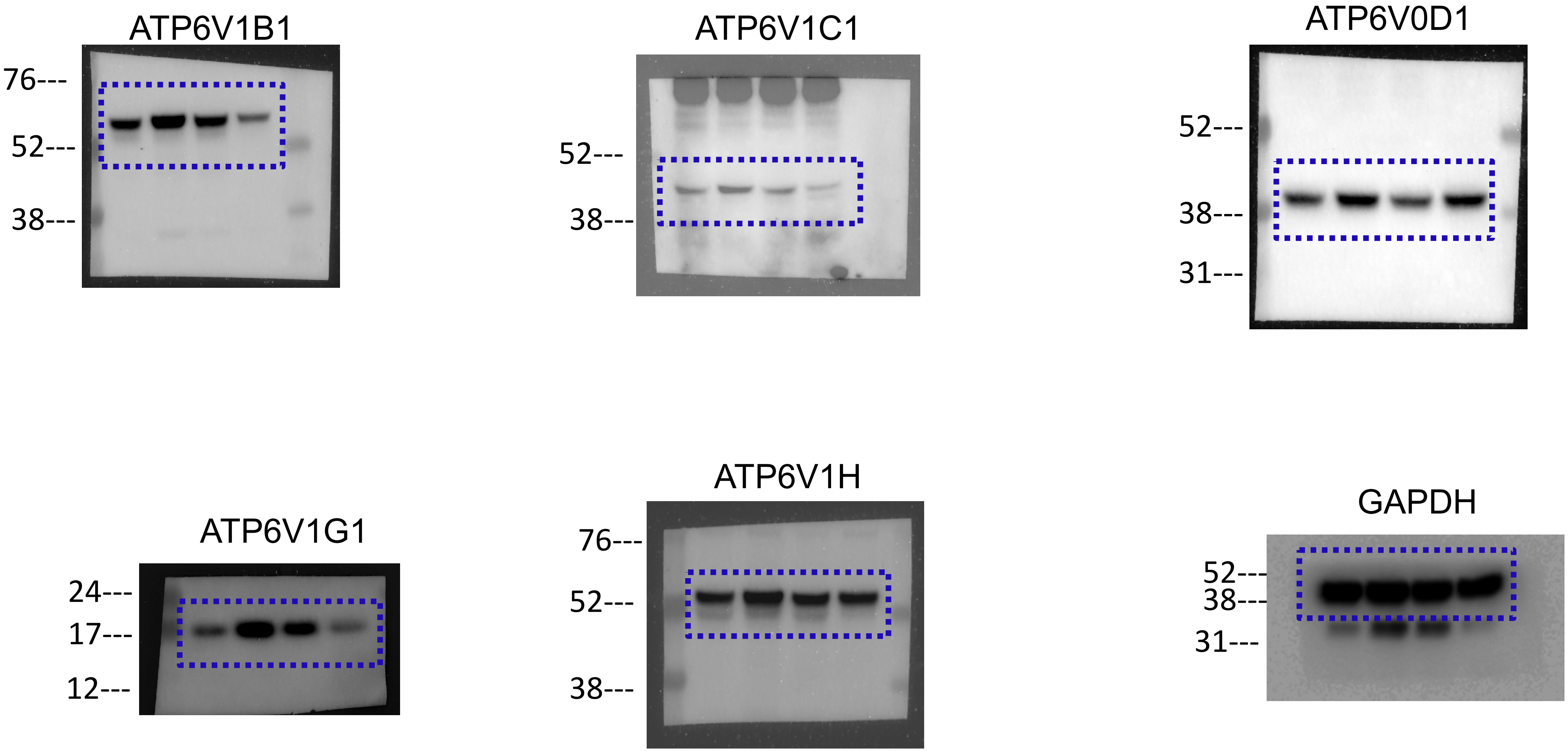

FIGURE 4E

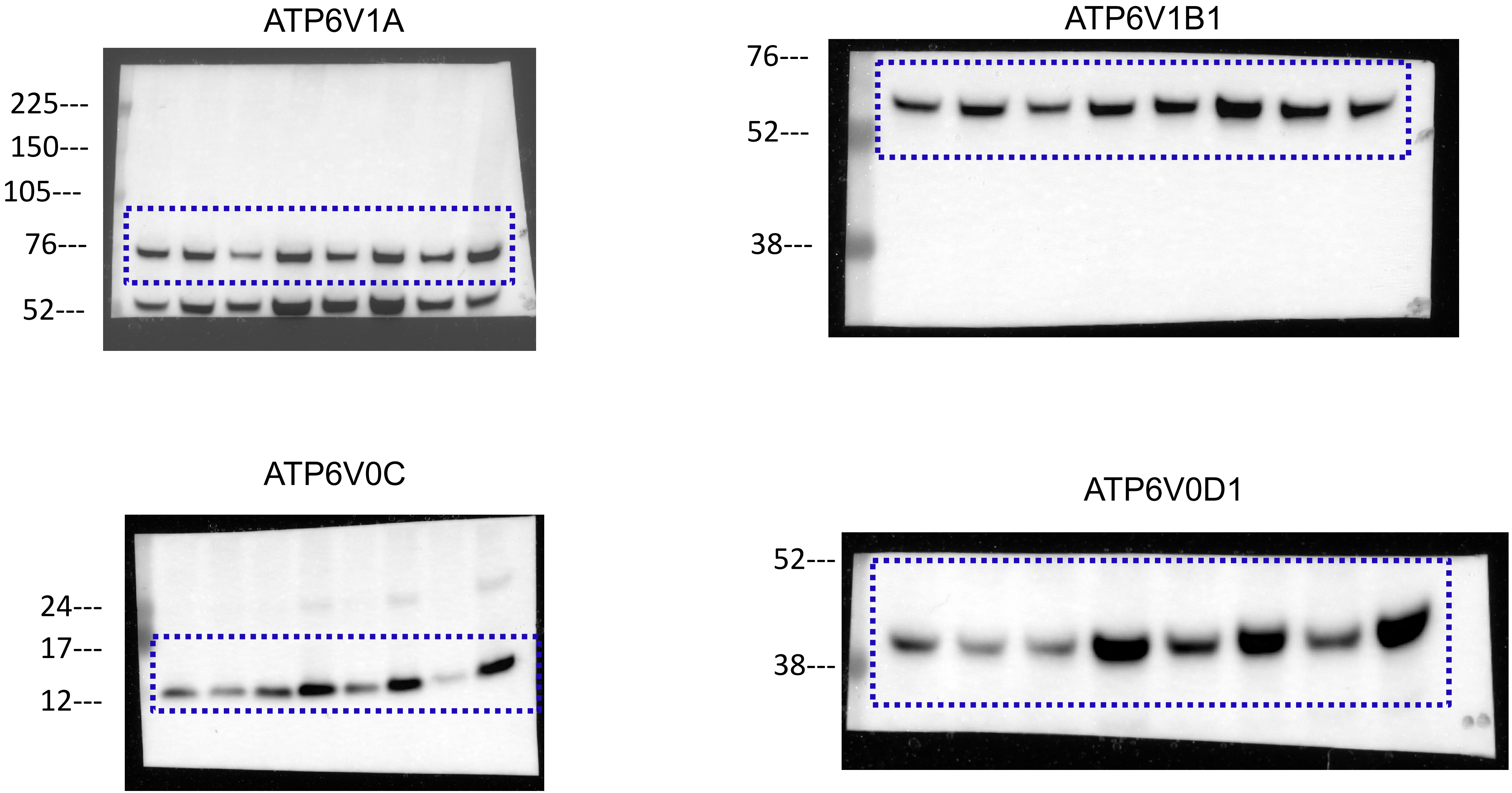

ATP6V1A
225---
150---
105---
76---
52---
ATP6V1B1
76---
52---
38---
ATP6V0C
24---
17---
12---
ATP6V0D1
52---
38---

FIGURE 4E

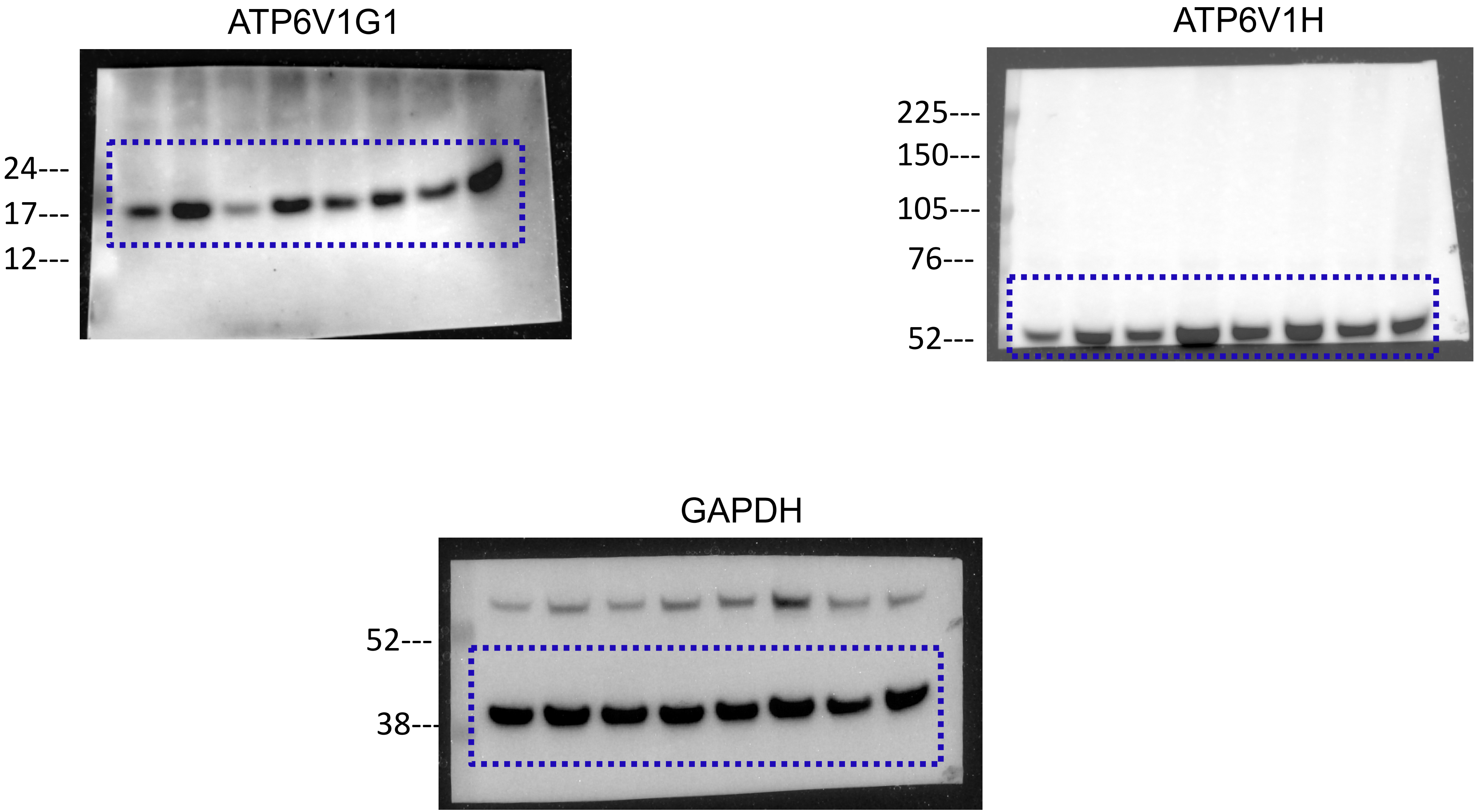

FIGURE 4F (Replicate 1 for quants)

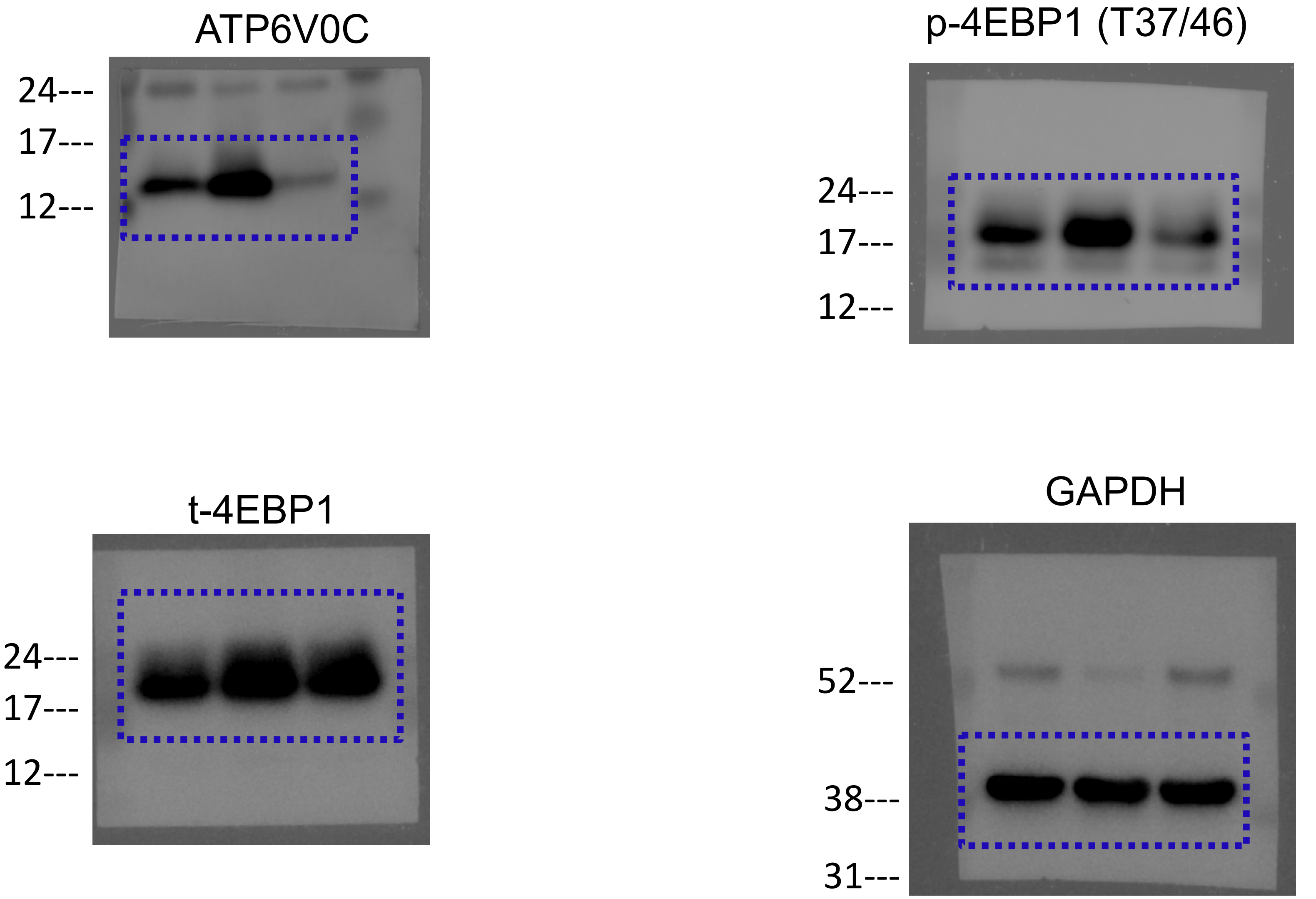

FIGURE 4F (Replicate 2 and 3 for quants)

ATP6V0C

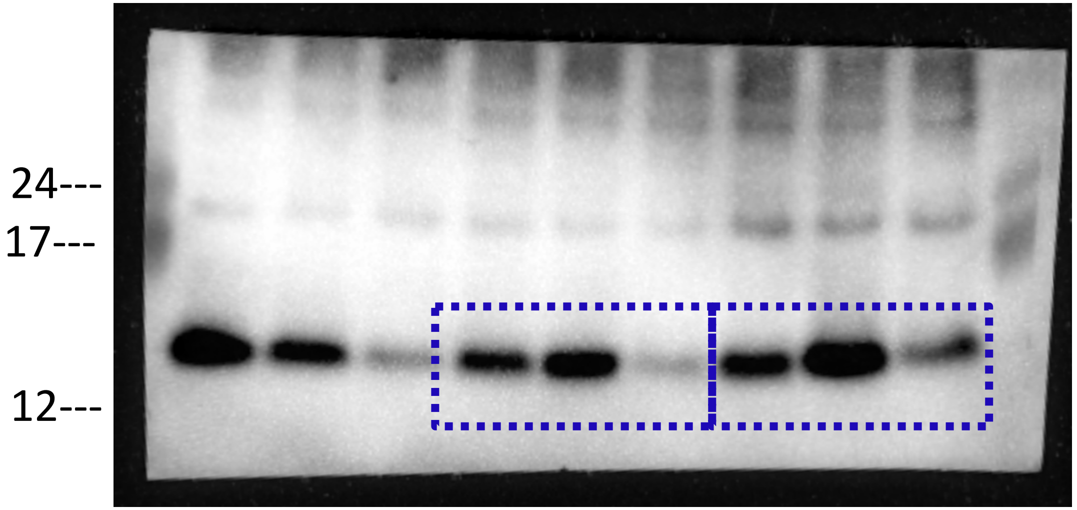

GAPDH

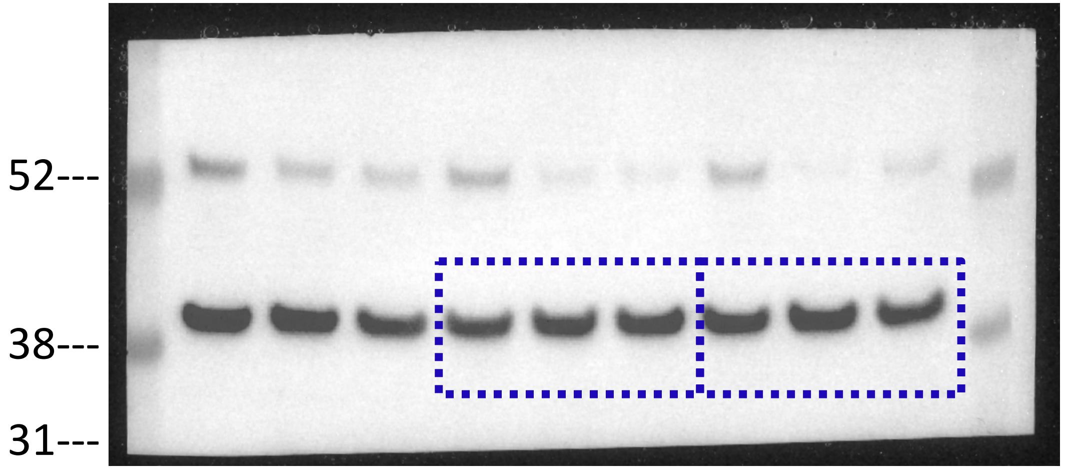

FIGURE 4G

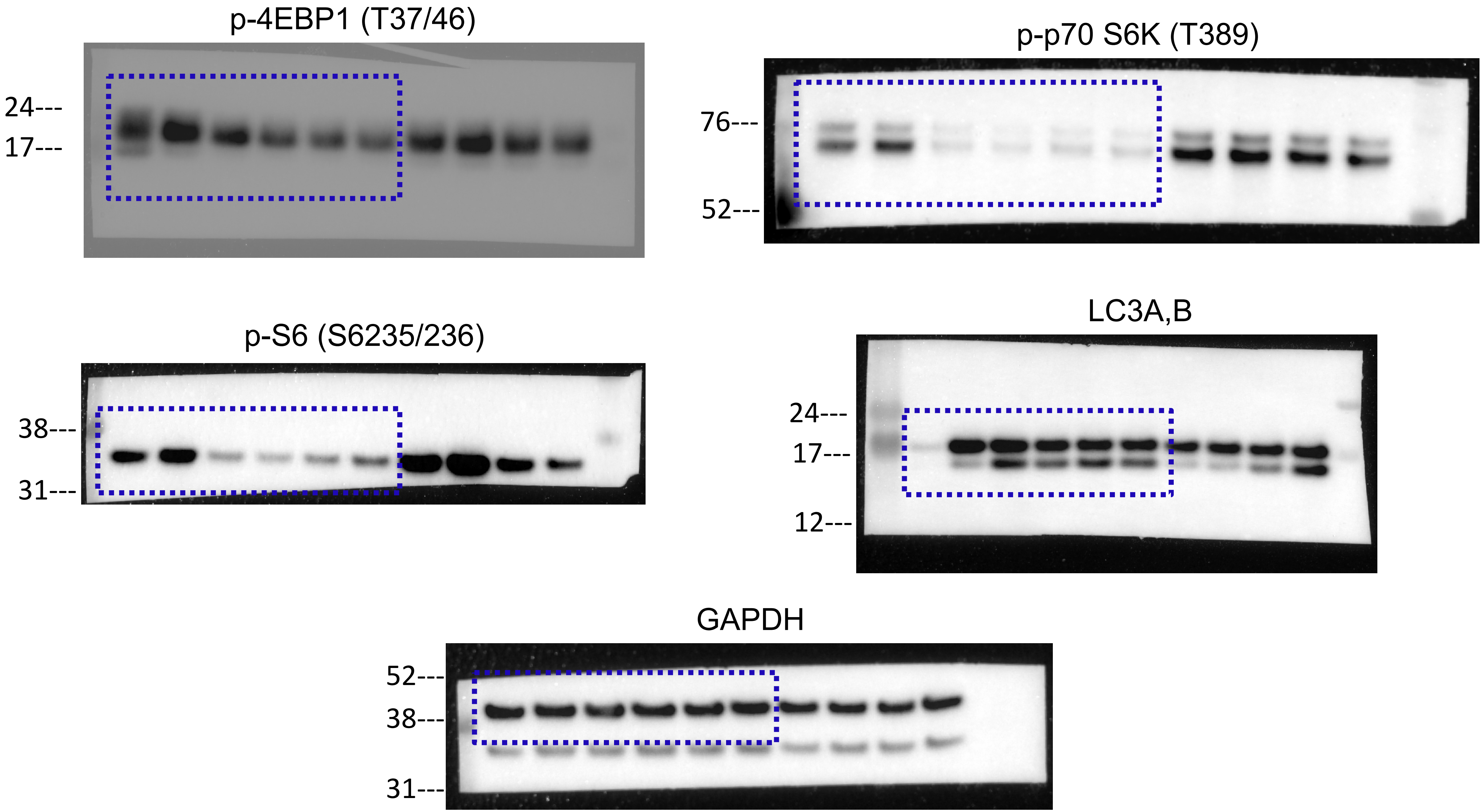
p-4EBP1 (T37/46)
24---
17---
p-p70 S6K (T389)
76---
52---
p-S6 (S6235/236)
38---
31---
LC3A,B
24---
17---
12---
GAPDH
52---
38---
31---

FIGURE 4G

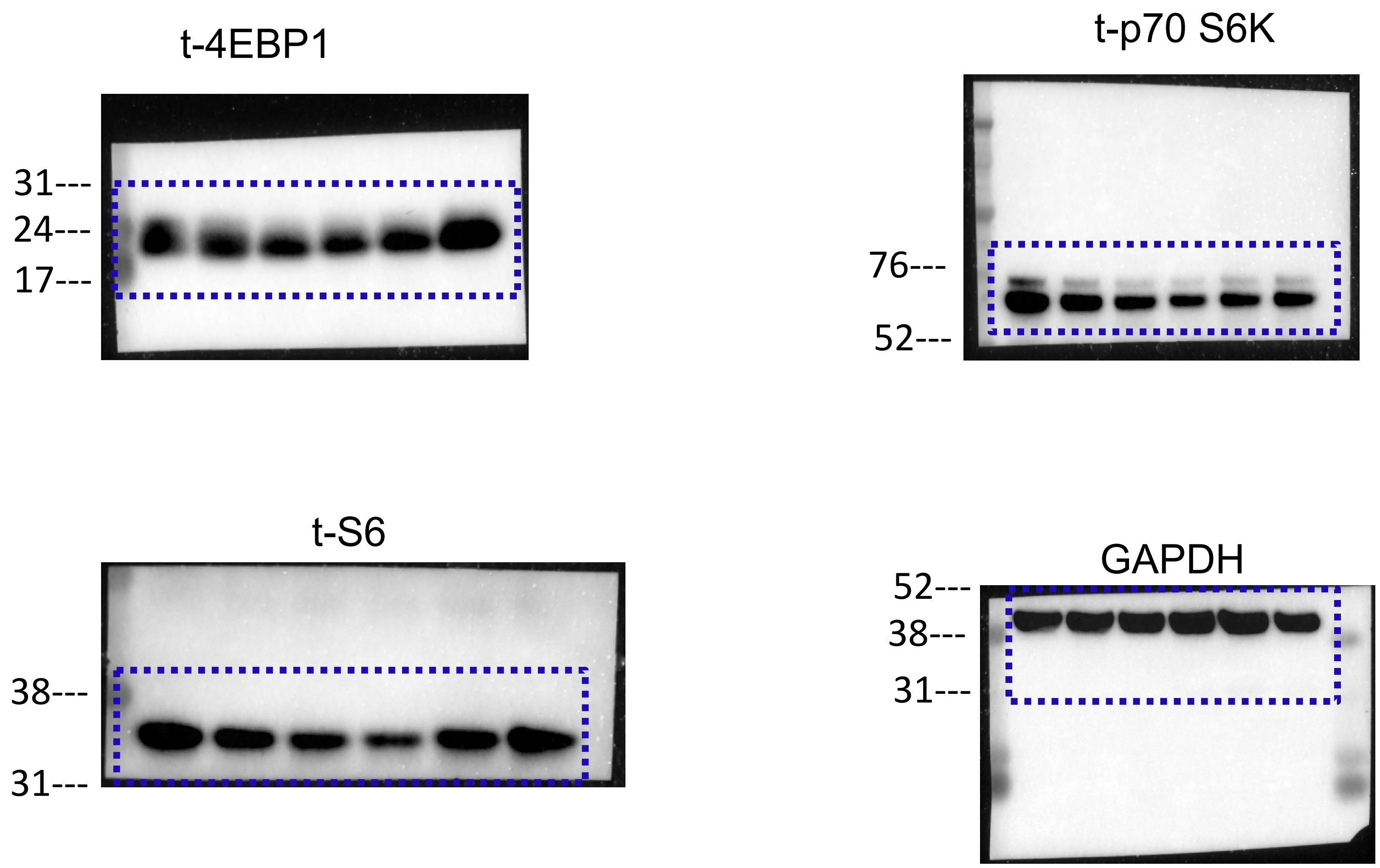

FIGURE 5H

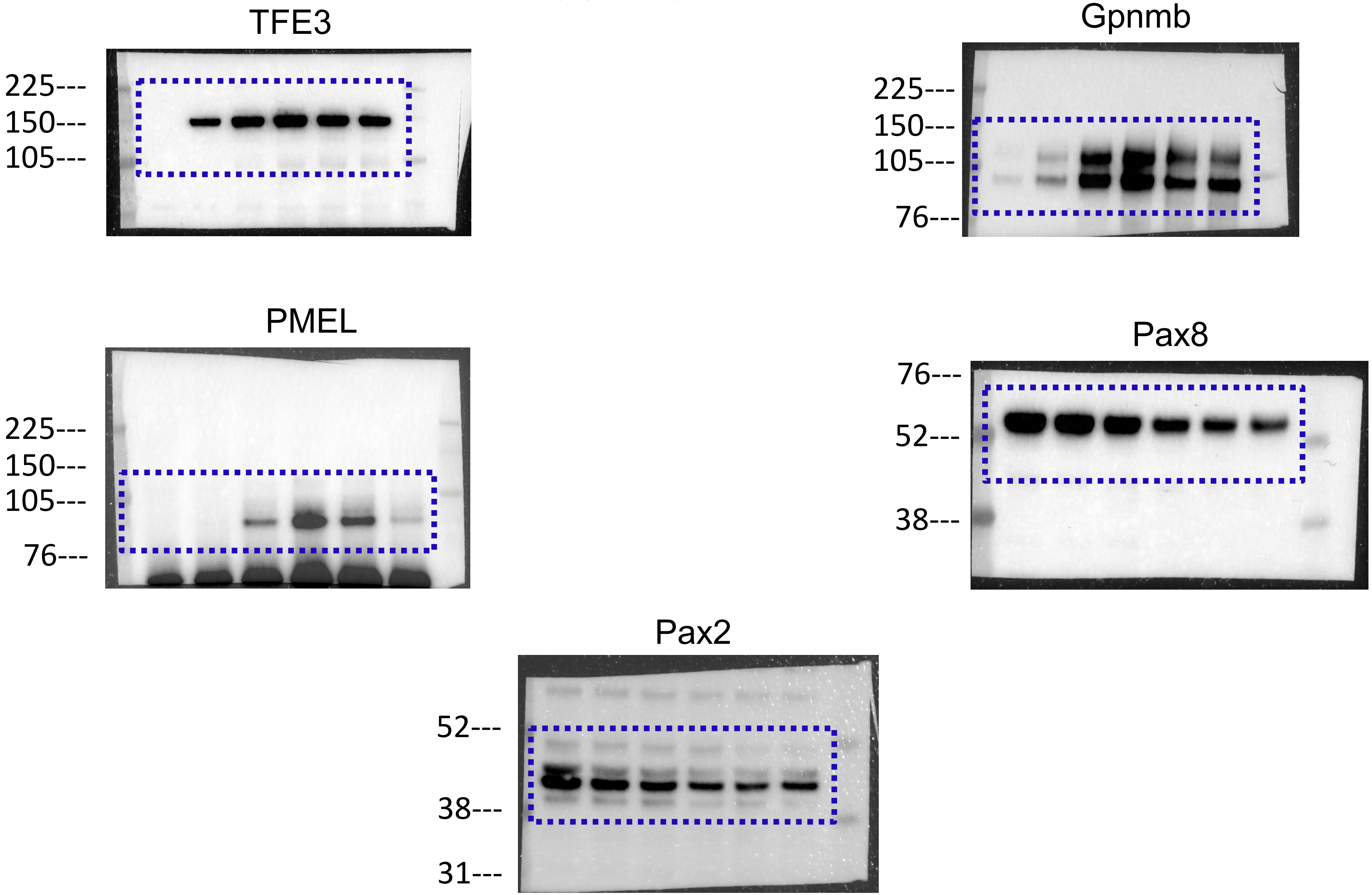
TFE3
225---
150---
105---
Gpnmb
225---
150---
105---
76---
PMEL
225---
150---
105---
76---
Pax8
76---
52---
38---
Pax2
52---
38---
31---

FIGURE 5H

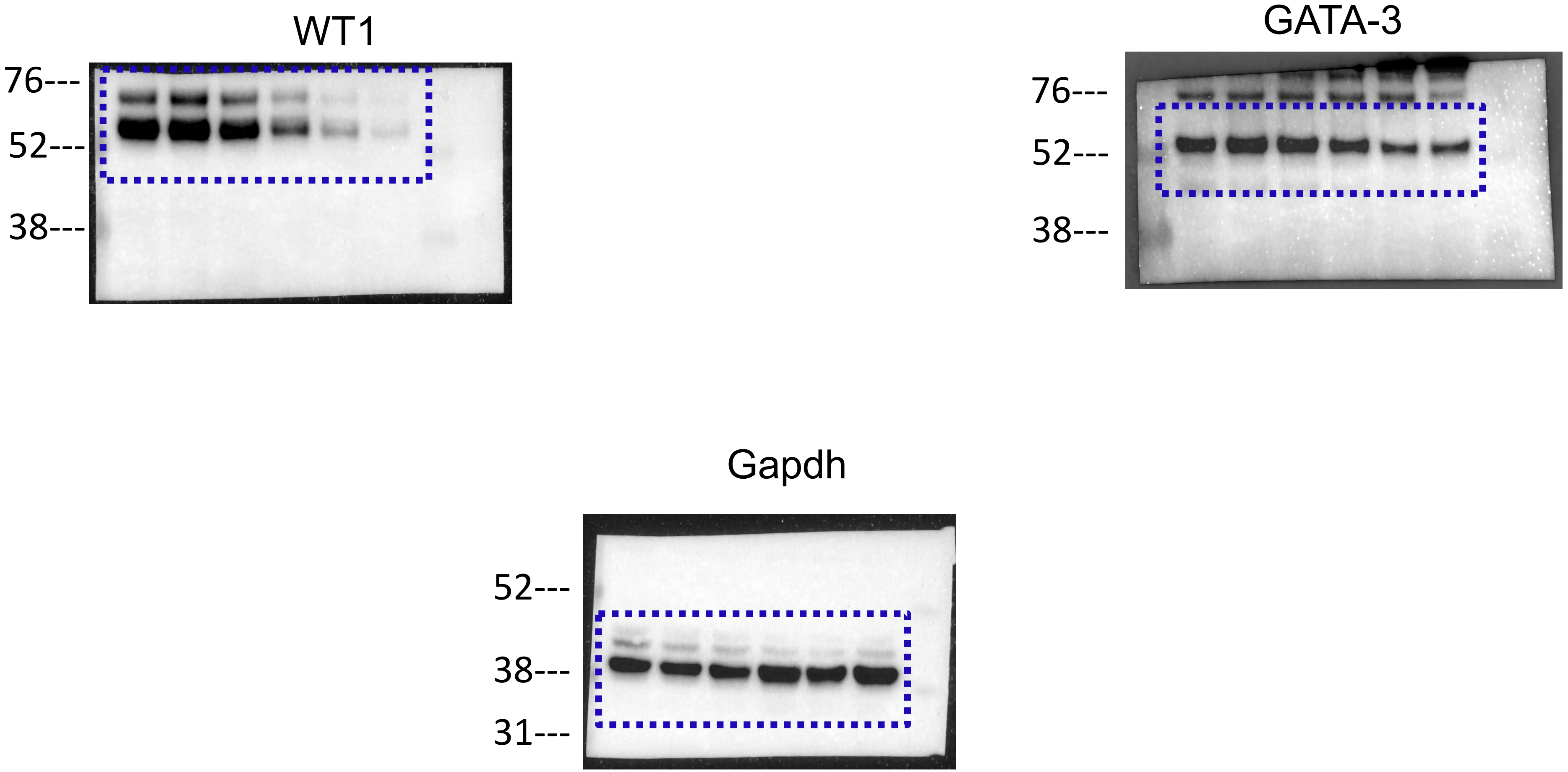

FIGURE 5H

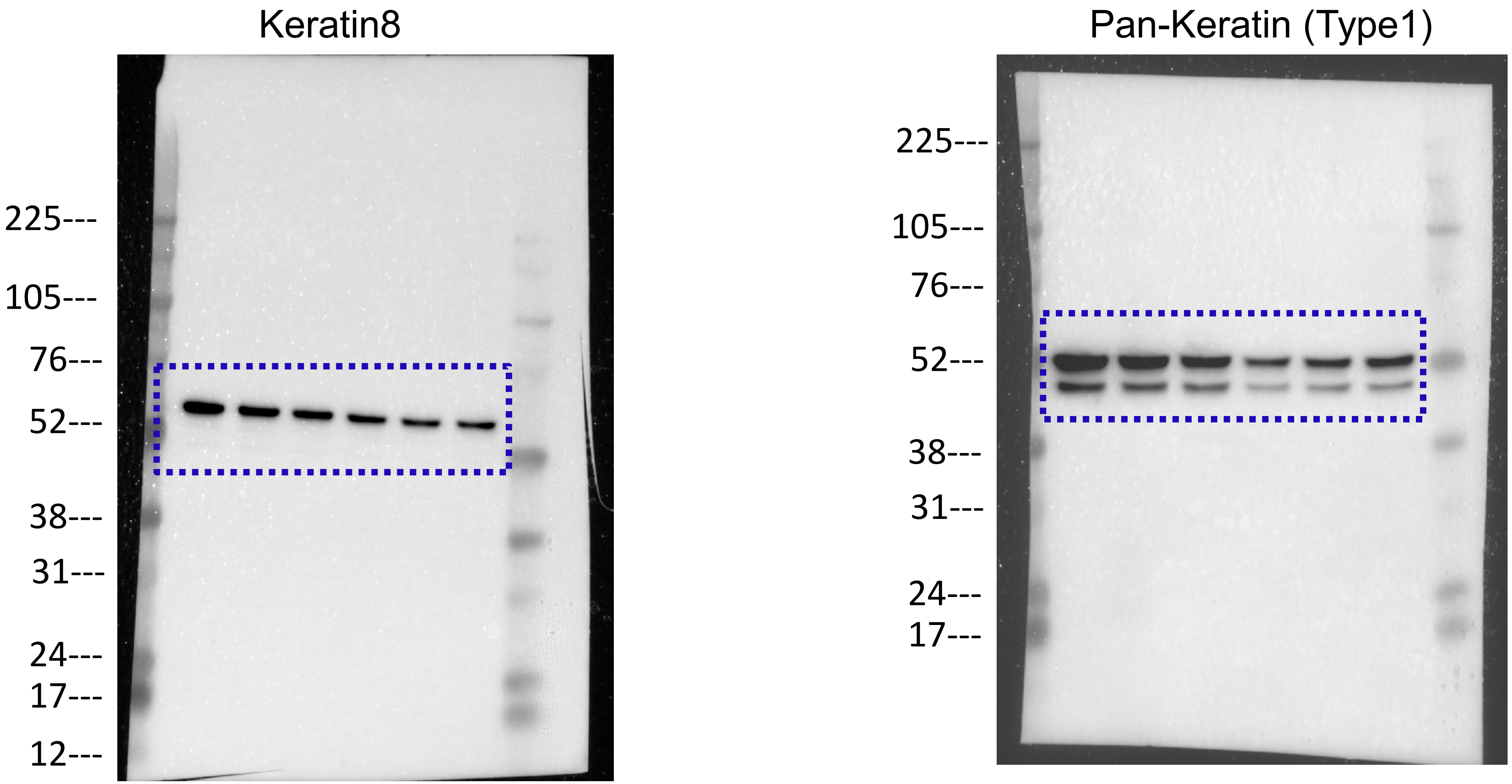
Keratin8
225---
105---
76---
52---
38---
31---
24---
17---
12---
Pan-Keratin (Type1)
225---
105---
76---
52---
38---
31---
24---
17---

FIGURE 6A

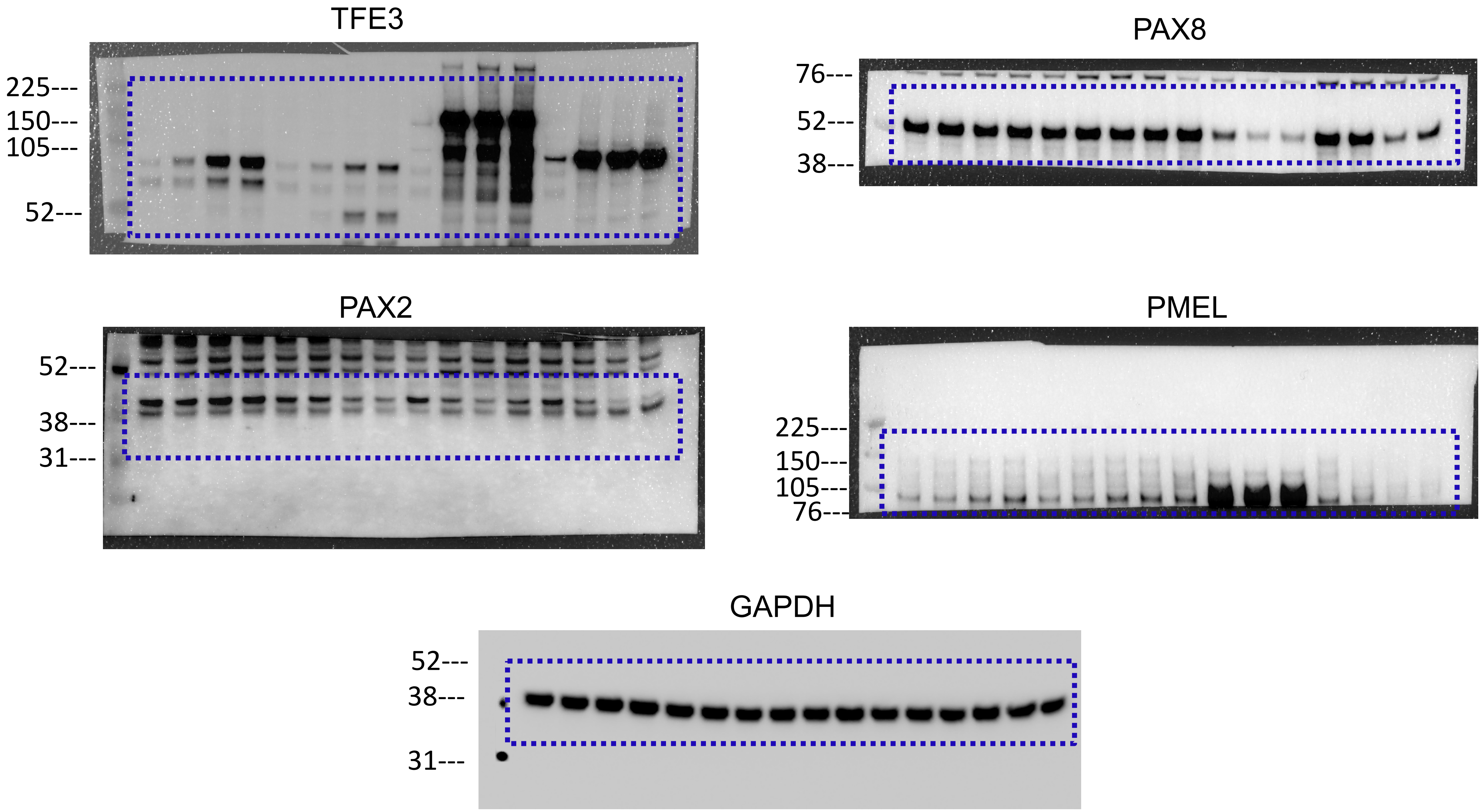
TFE3
225---
150---
105---
52---
PAX8
76---
52---
38---
PAX2
52---
38---
31---
PMEL
225---
150---
105---
76---
GAPDH
52---
38---
31---

FIGURE 6B

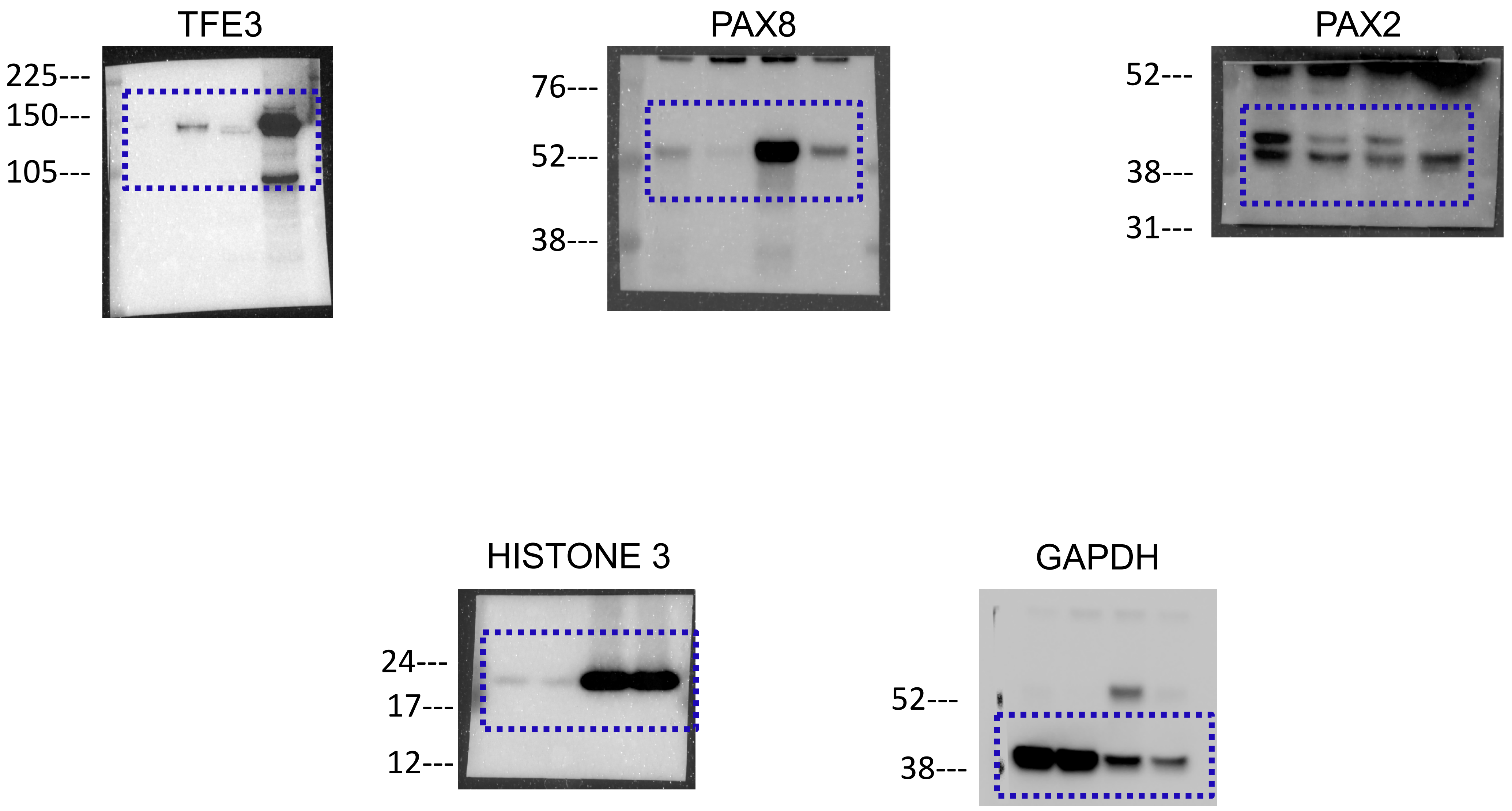
TFE3
225---
150---
105---
PAX8
76---
52---
38---
PAX2
52---
38---
31---
HISTONE 3
24---
17---
12---
GAPDH
52---
38---

FIGURE 6F

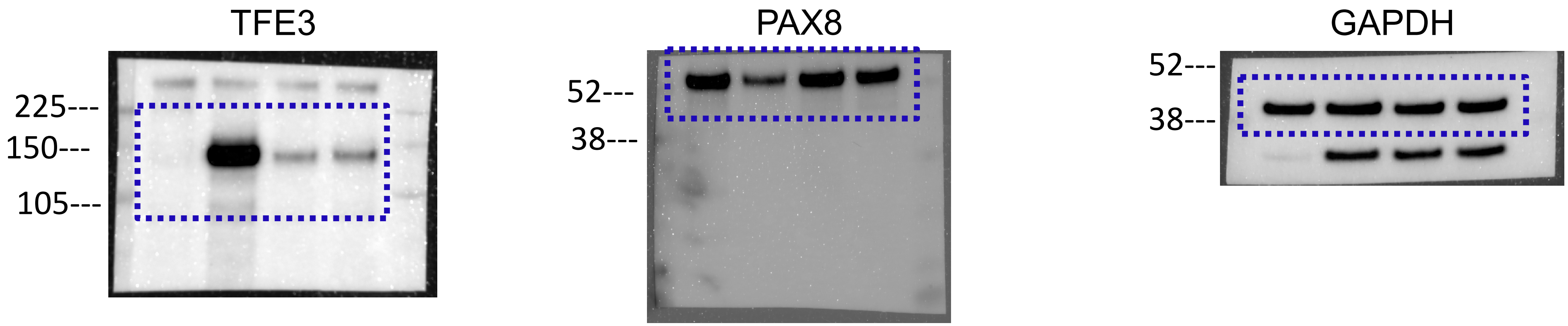

FIGURE 6G (Replicate 1 for Quants in 6H)

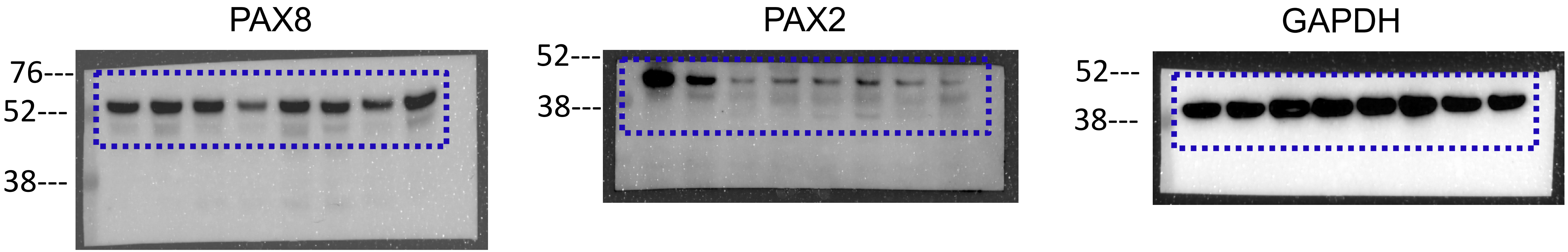

FIGURE 6G (Replicate 2 for Quants in 6H)

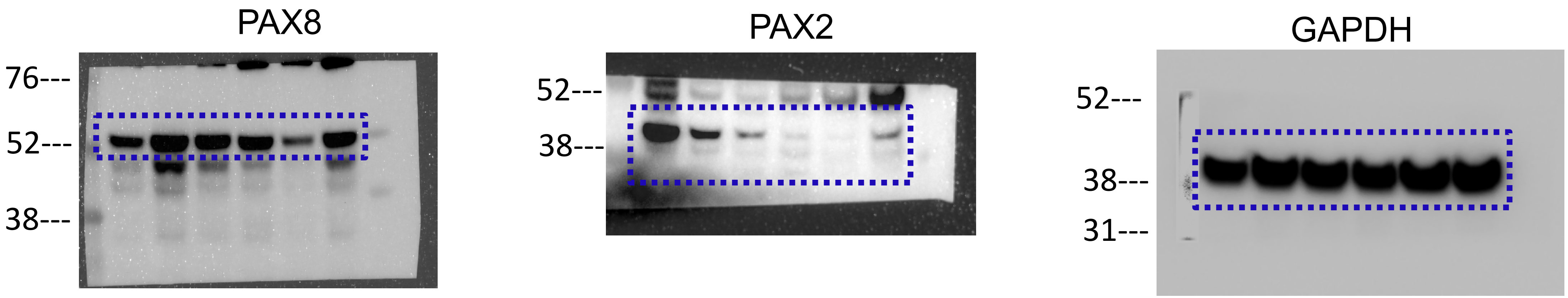

FIGURE 6G (Replicate 3 for Quants in 6H)

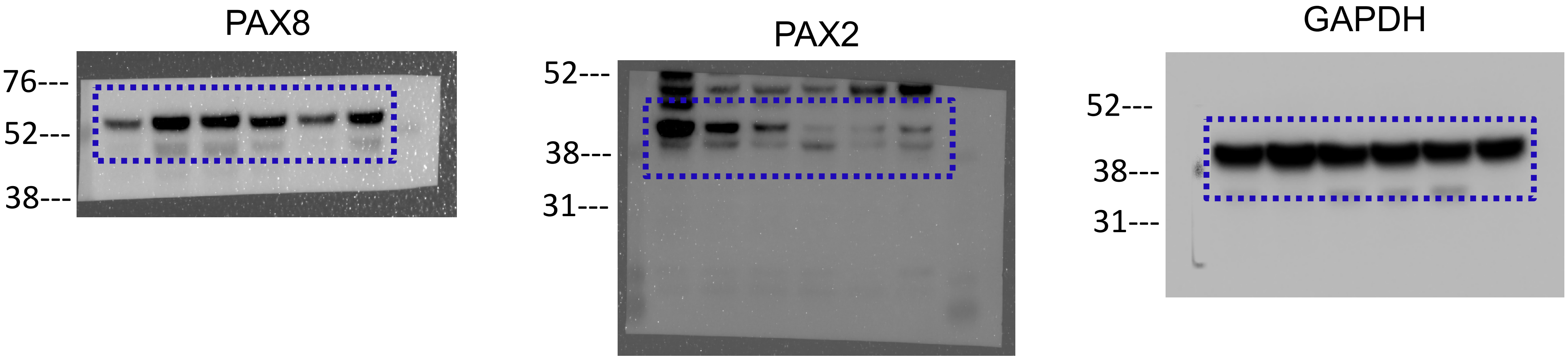

FIGURE 6G (Replicate 4 for Quants in 6H)

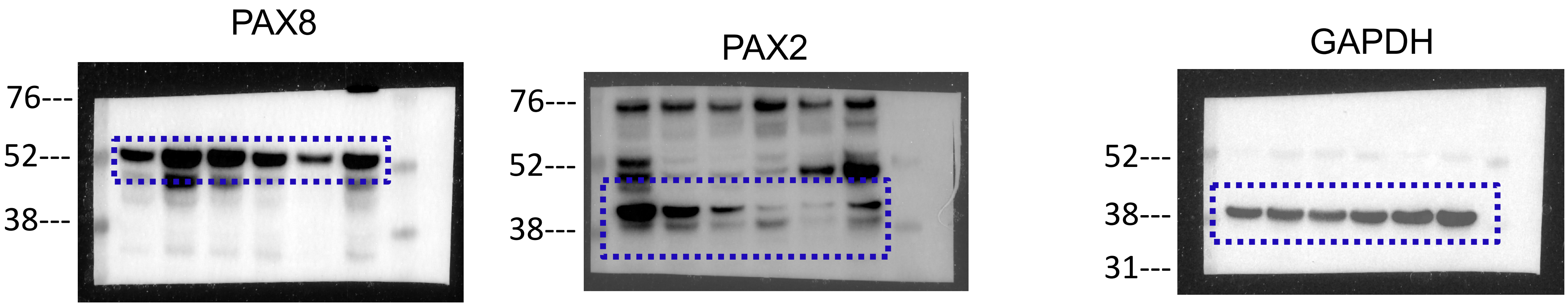

FIGURE 7A-Replicate 1 for quants in 7B

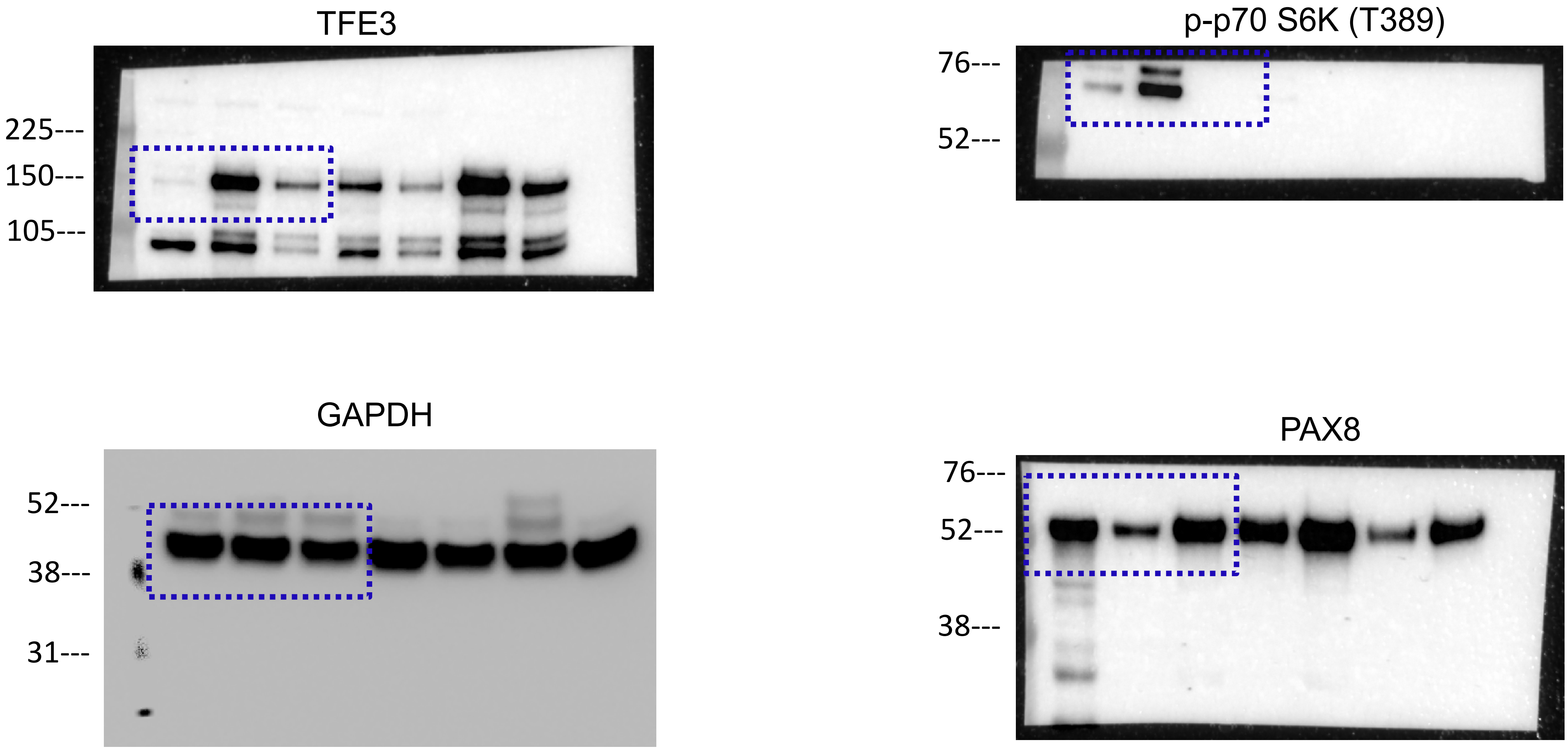

FIGURE 7A-Replicate 1 for quants in 7B

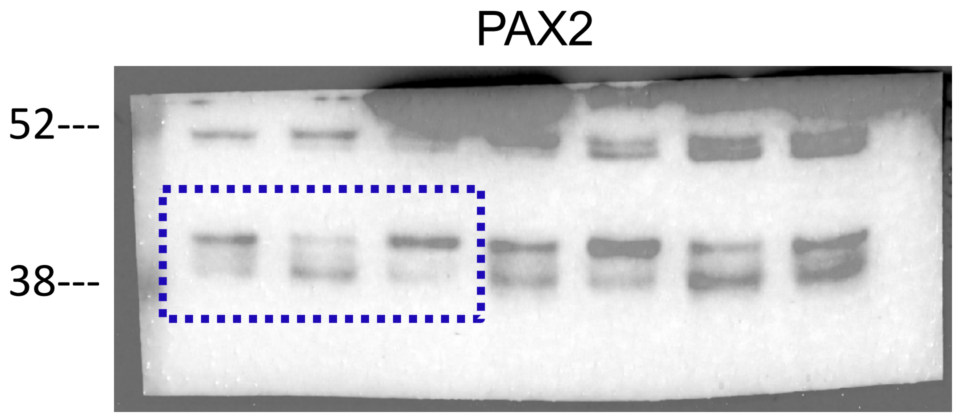

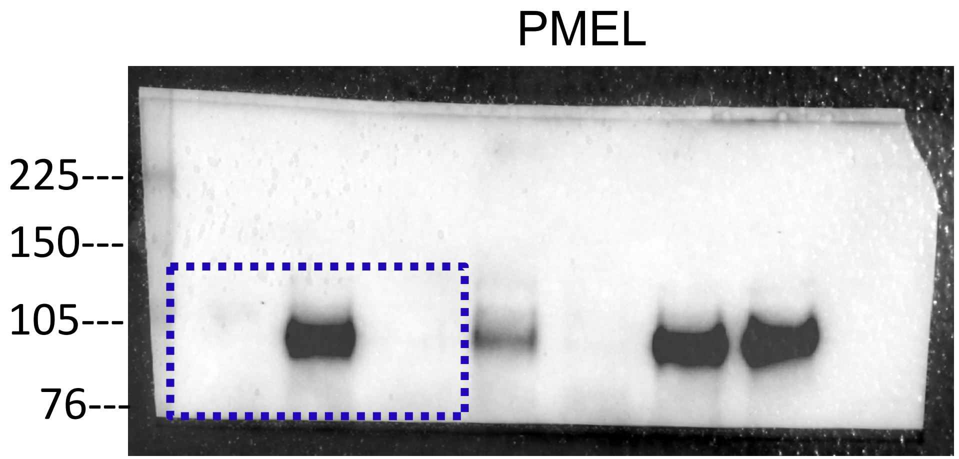

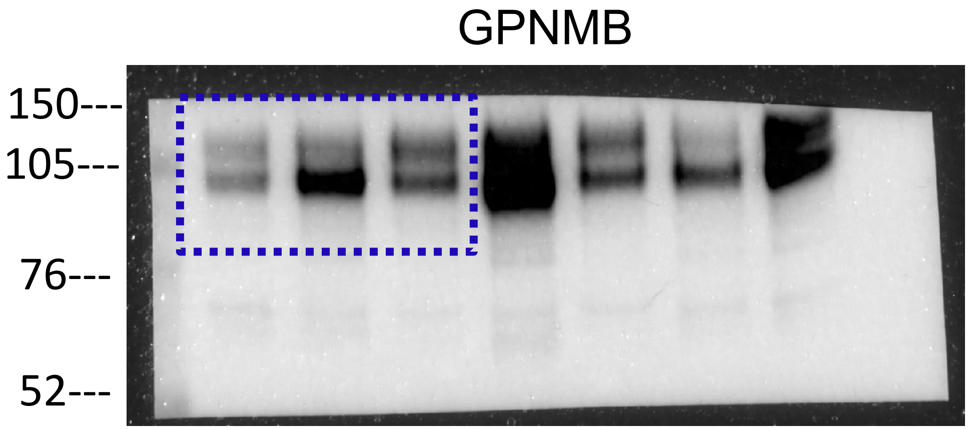

FIGURE 7A-Replicate 1 for quants in 7B

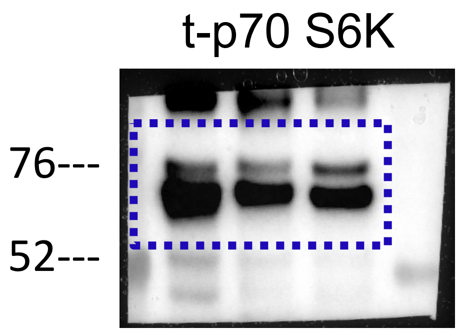

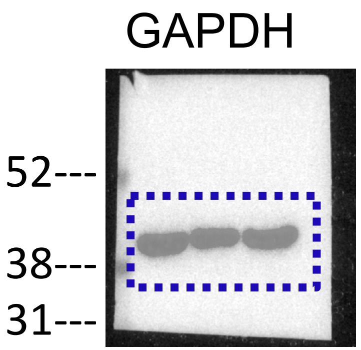

FIGURE 7A-Replicate 2 for quants in 7B (See S9B)

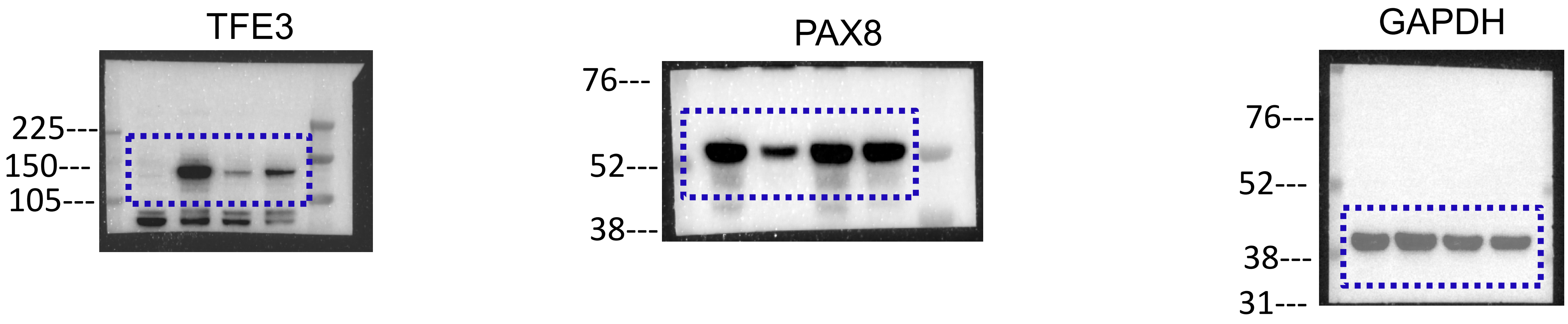

FIGURE 7A-Replicate 3 for quants in 7B

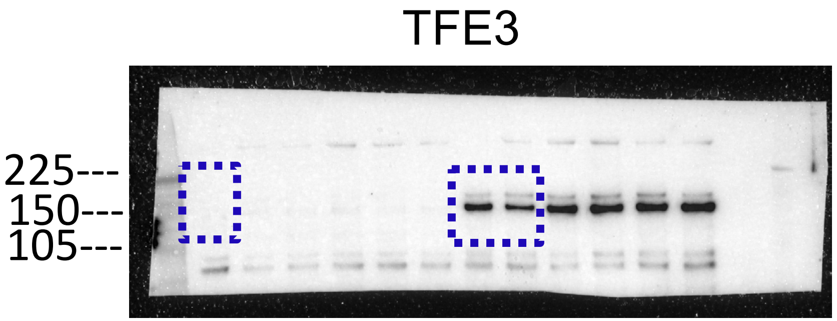

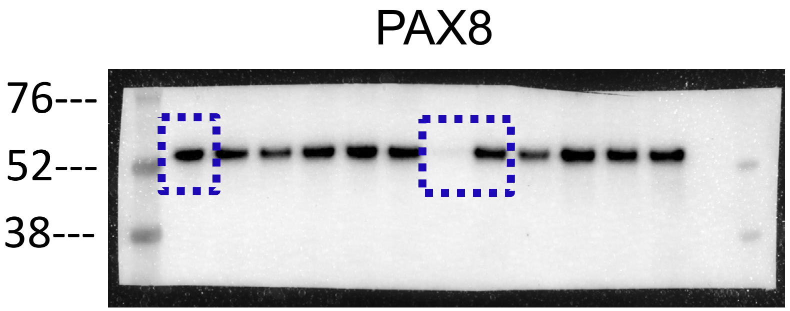

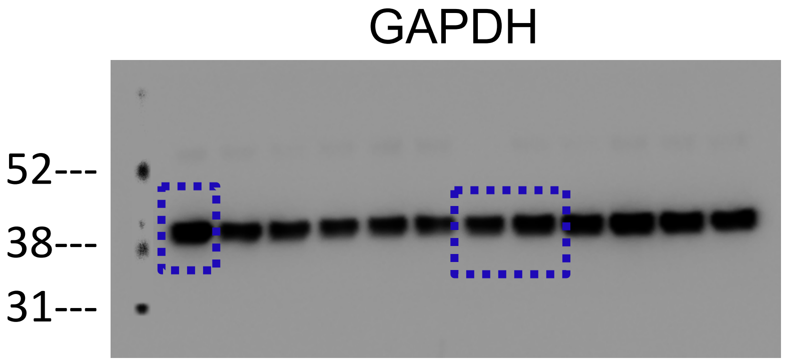

FIGURE 7A-Replicate 4 for quants in 7B (See S9A)

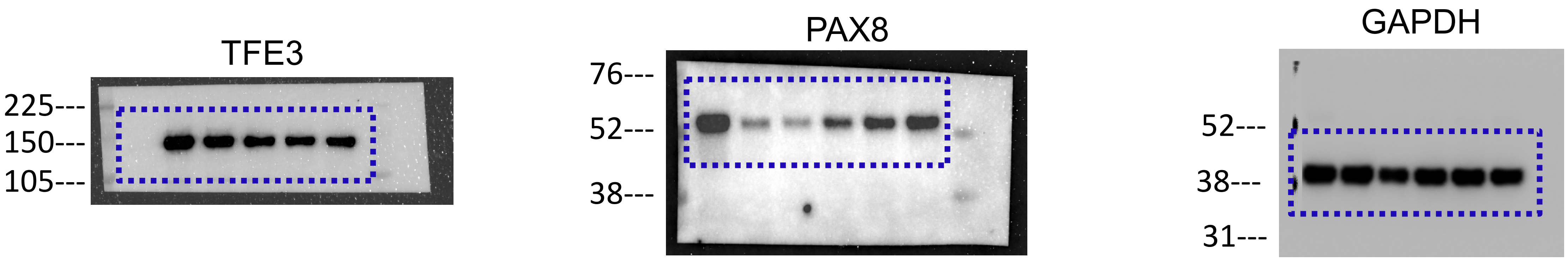

FIGURE 7C- Replicate 1 for quants in 7D

RHEB

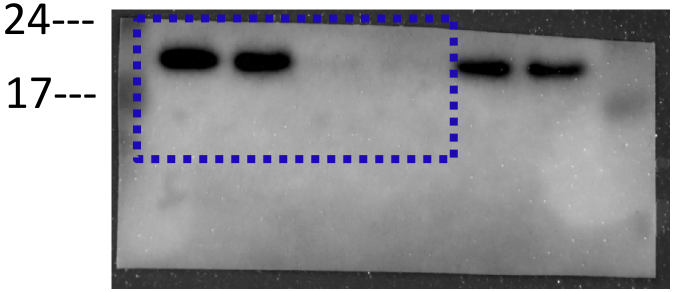

p-S6 (S6235/236)

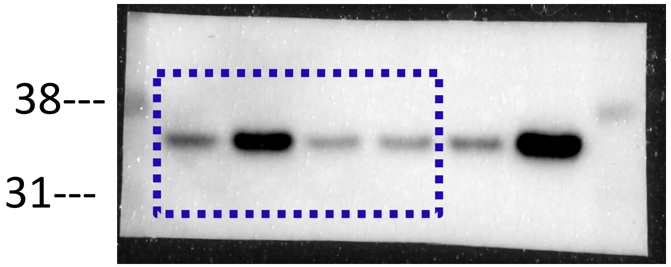

p-4EBP1 (S65)

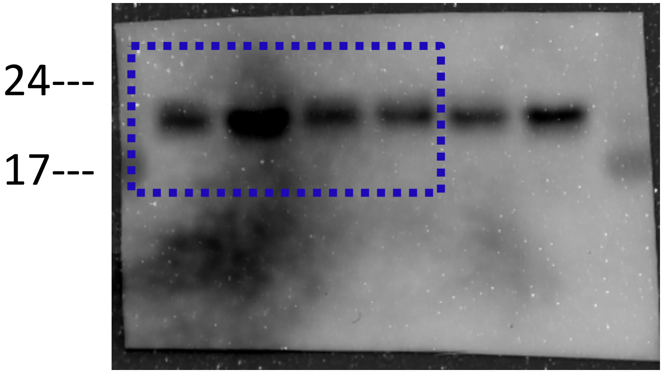

RRAGD

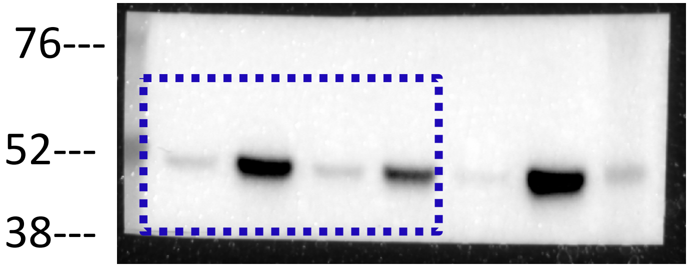

FIGURE 7C- Replicate 1 for quants in 7D

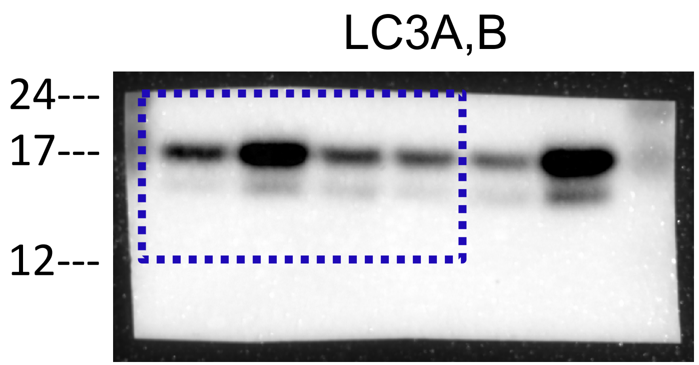

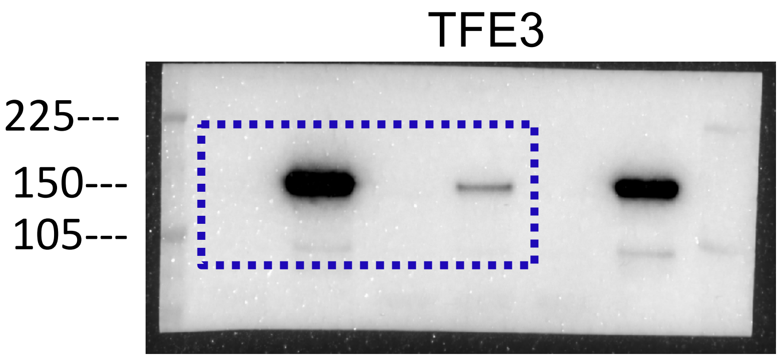

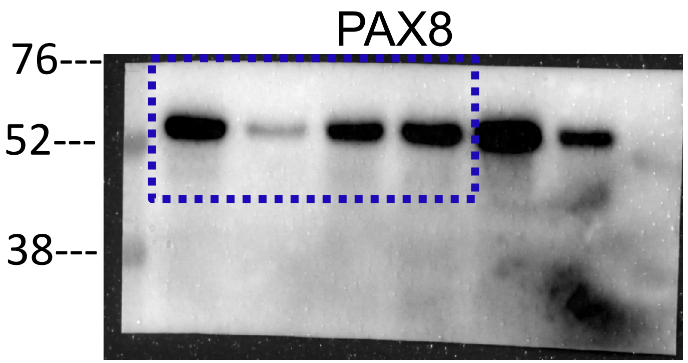

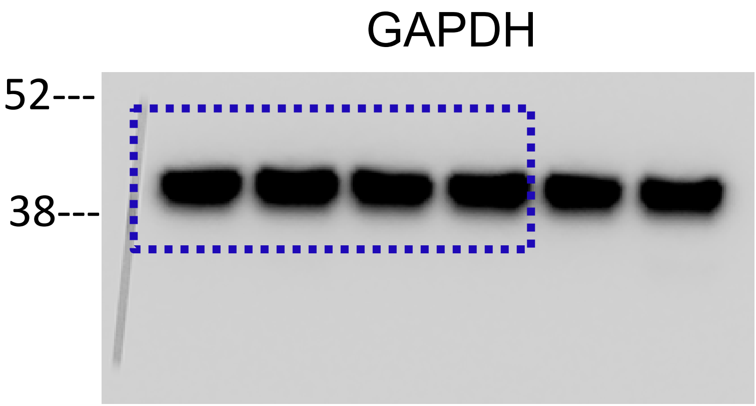

FIGURE 7C- Replicate 2 for quants in 7D

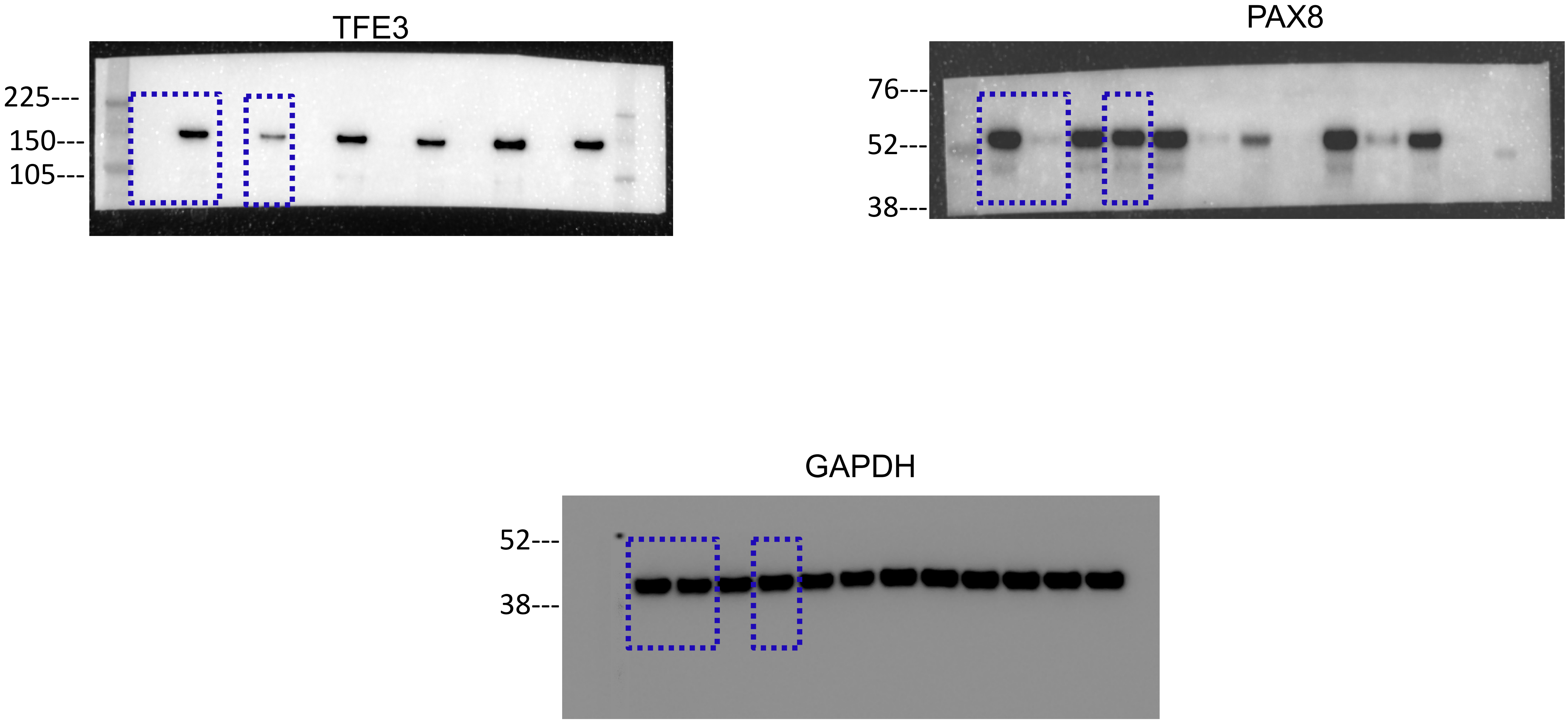

FIGURE 7C- Replicate 3 for quants in 7D

TFE3

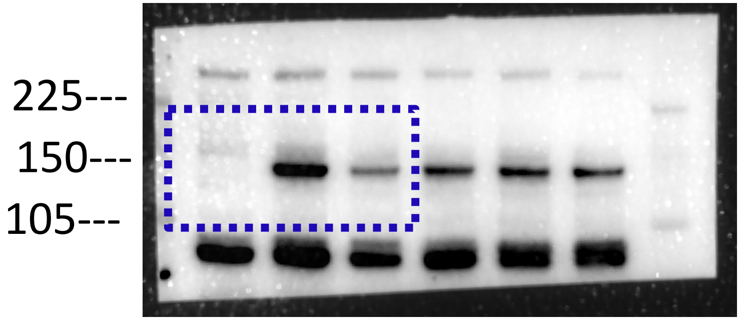

PAX8

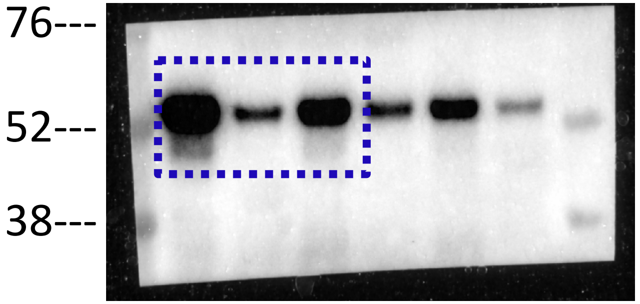

GAPDH

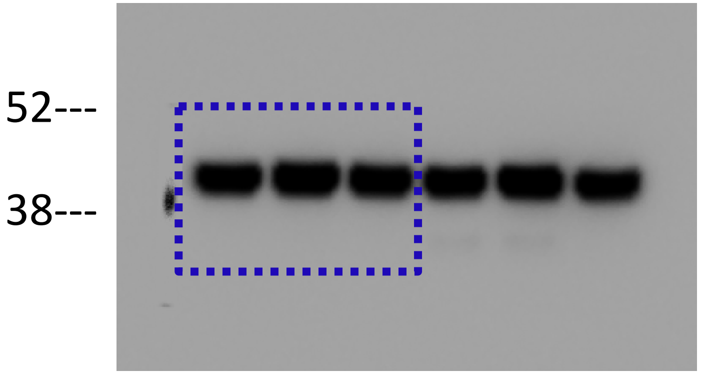

FIGURE 7C- Replicate 4 for quants in 7D (See S9B)

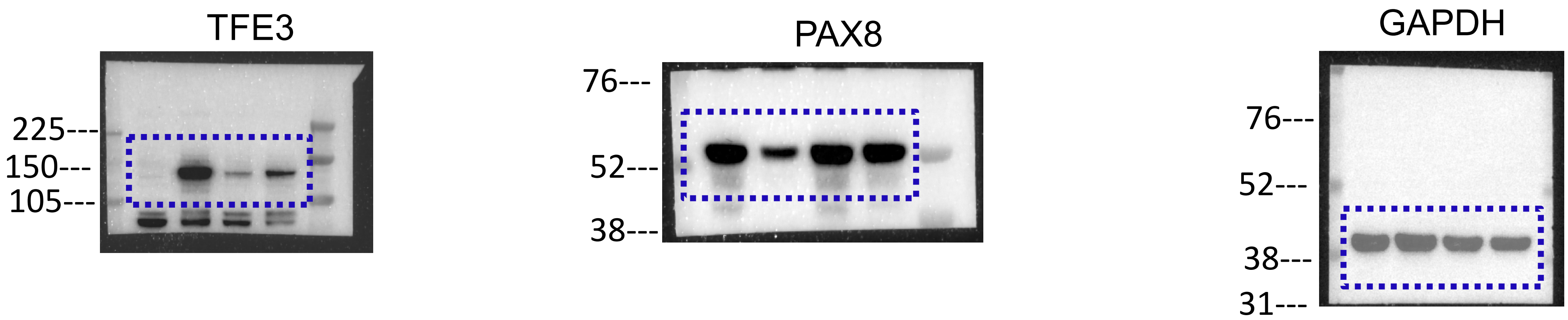

FIGURE 7C- Replicate 5 for quants in 7D

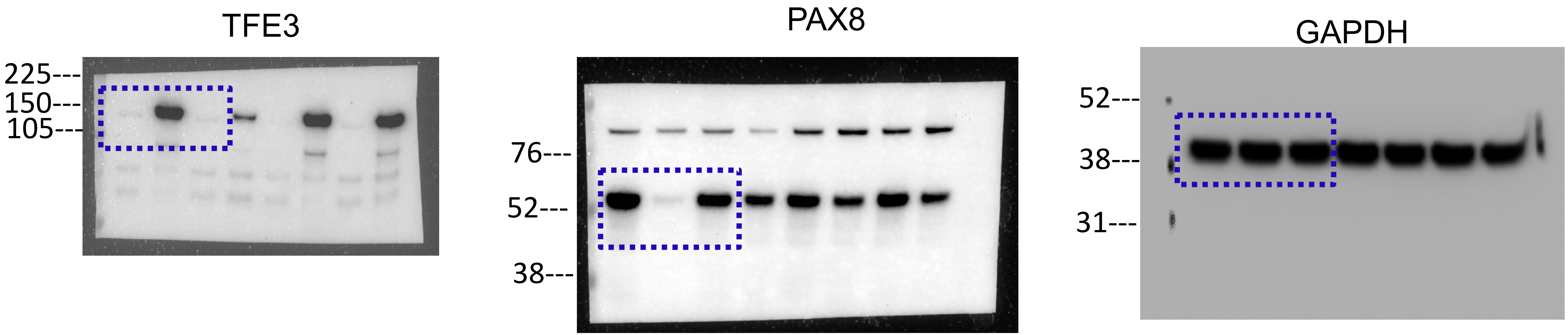

FIGURE 7E

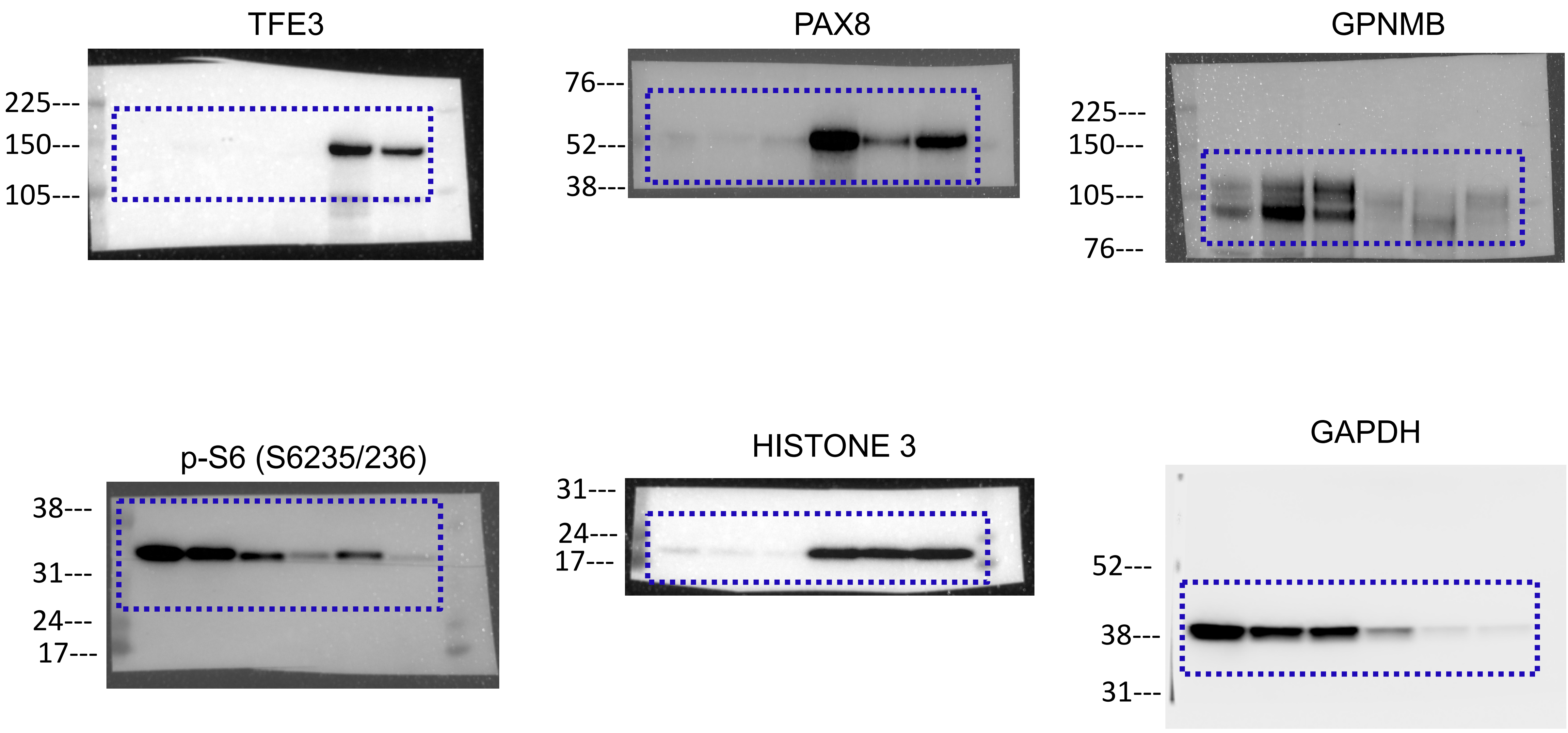
TFE3
225---
150---
105---
PAX8
76---
52---
38---
GPNMB
225---
150---
105---
76---
p-S6 (S6235/236)
38---
31---
24---
17---
HISTONE 3
31---
24---
17---
GAPDH
52---
38---
31---

FIGURE 7I (Replicate 1 for quants)

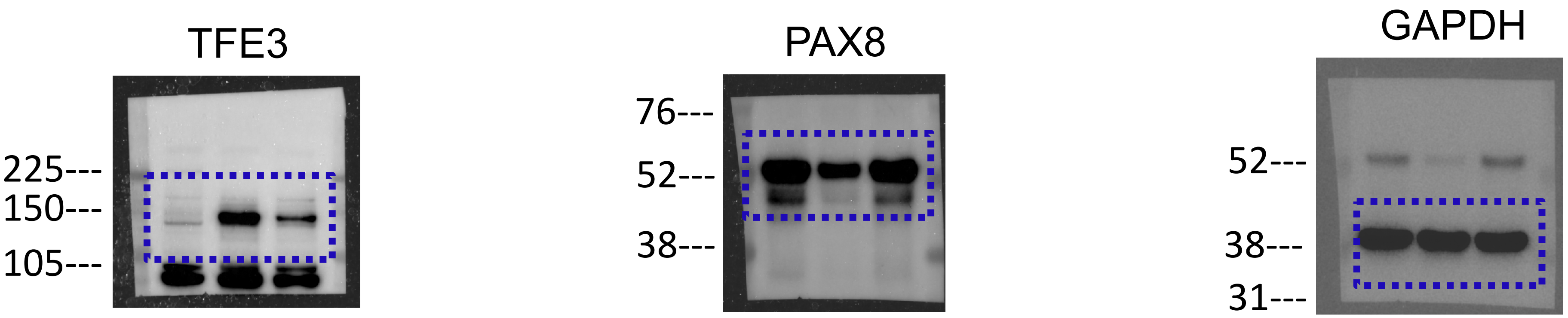

FIGURE 7I (Replicate 2 for quants)

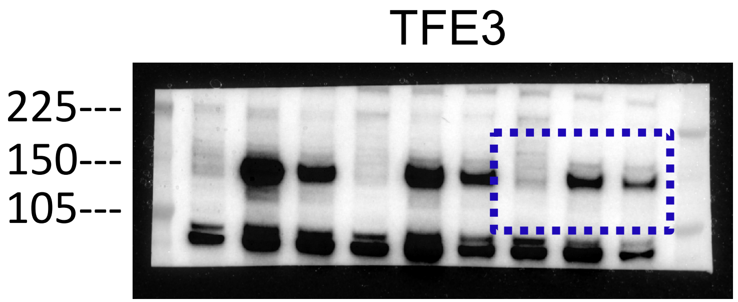

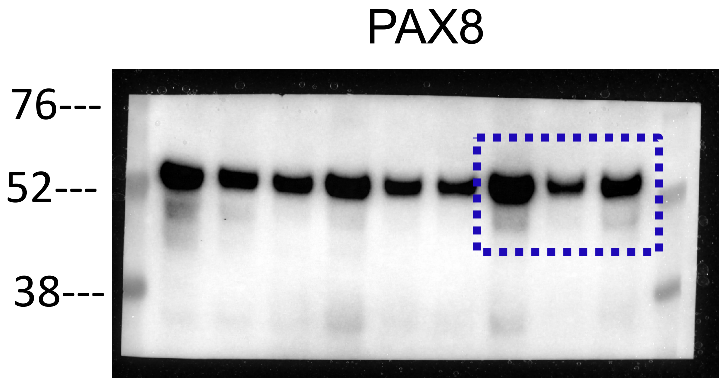

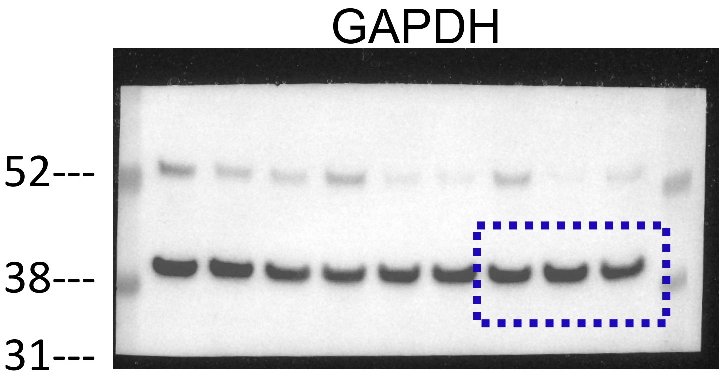

FIGURE 7I (Replicate 3 and 4 for quants)

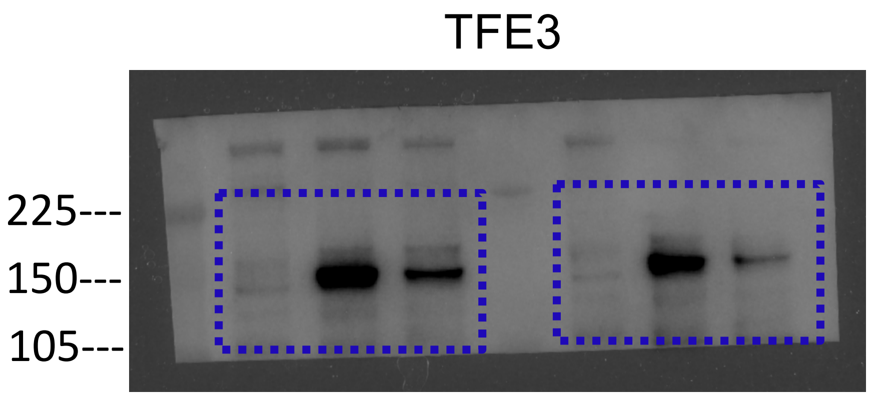

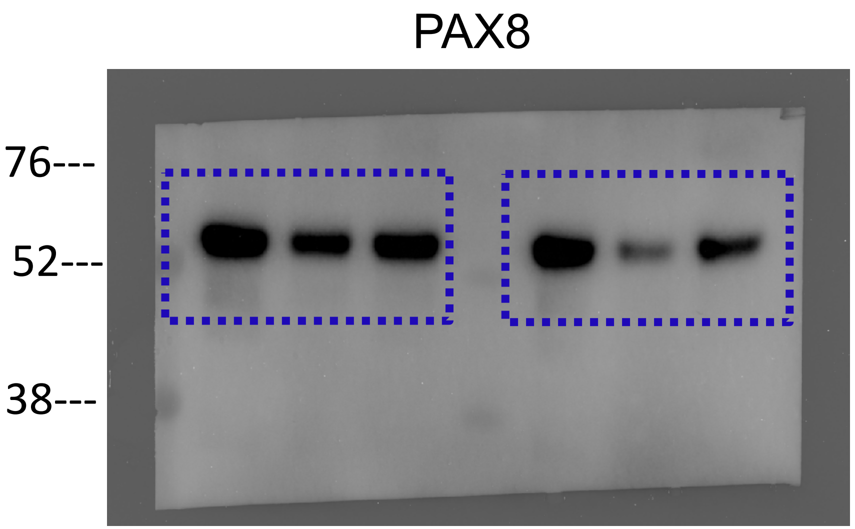

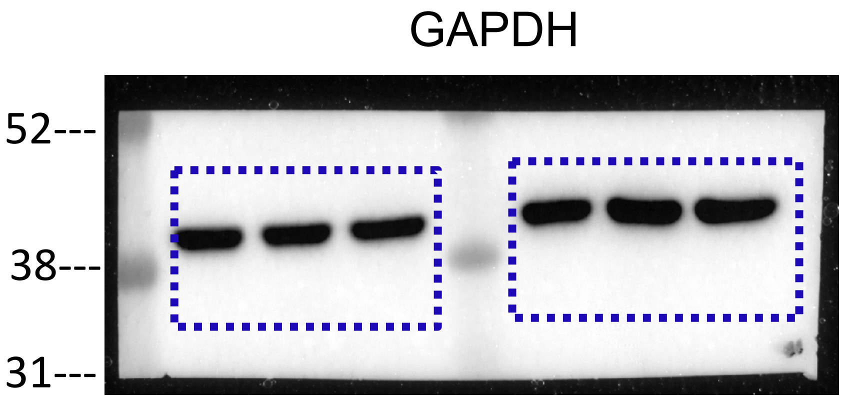

Uncropped and unprocessed gels

(Supplemental Figures)

FIGURE S1B

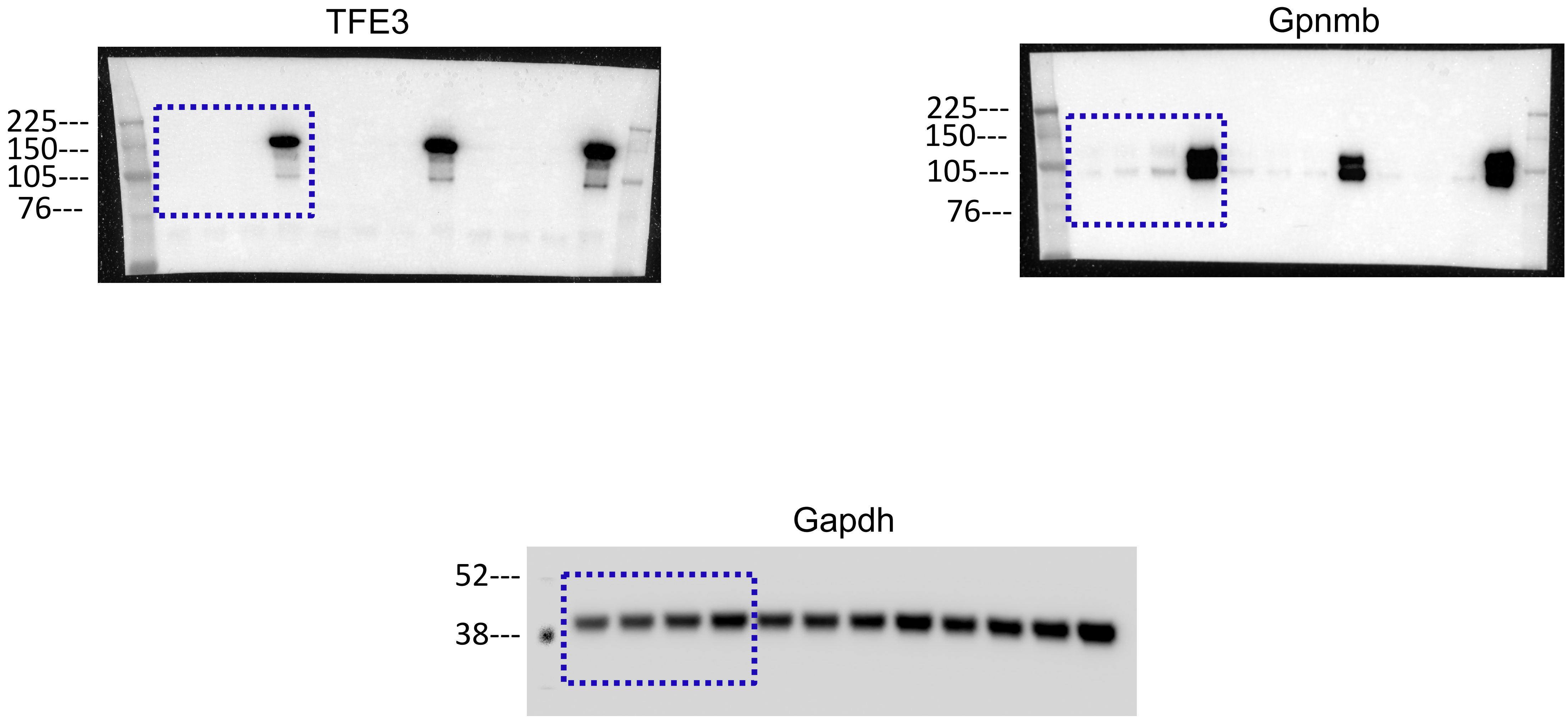

FIGURE S1C

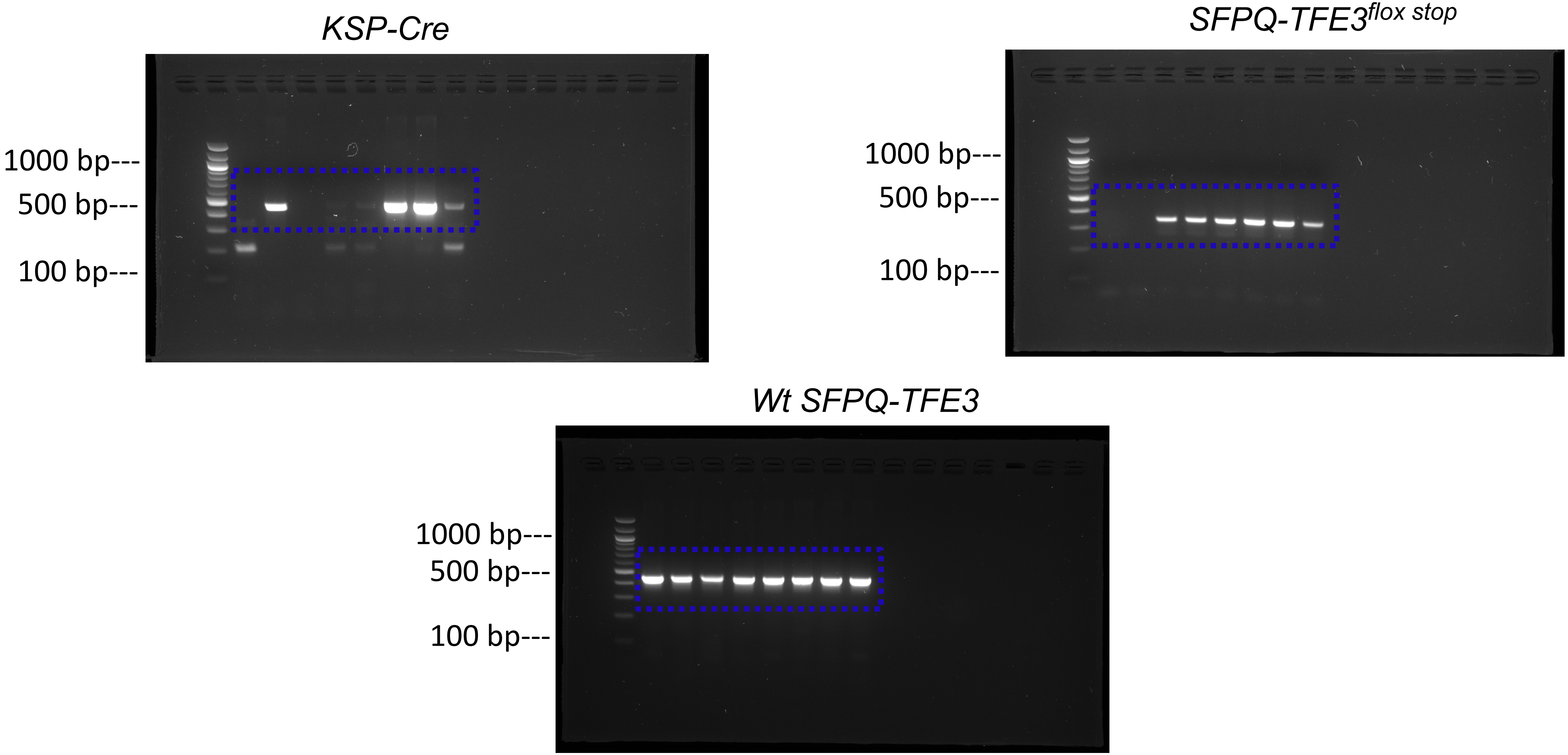

KSP-Cre
1000 bp---
500 bp---
100 bp---
SFPQ-TFE3flox stop
1000 bp---
500 bp---
100 bp---
Wt SFPQ-TFE3
1000 bp---
500 bp---
100 bp---

FIGURE S1D

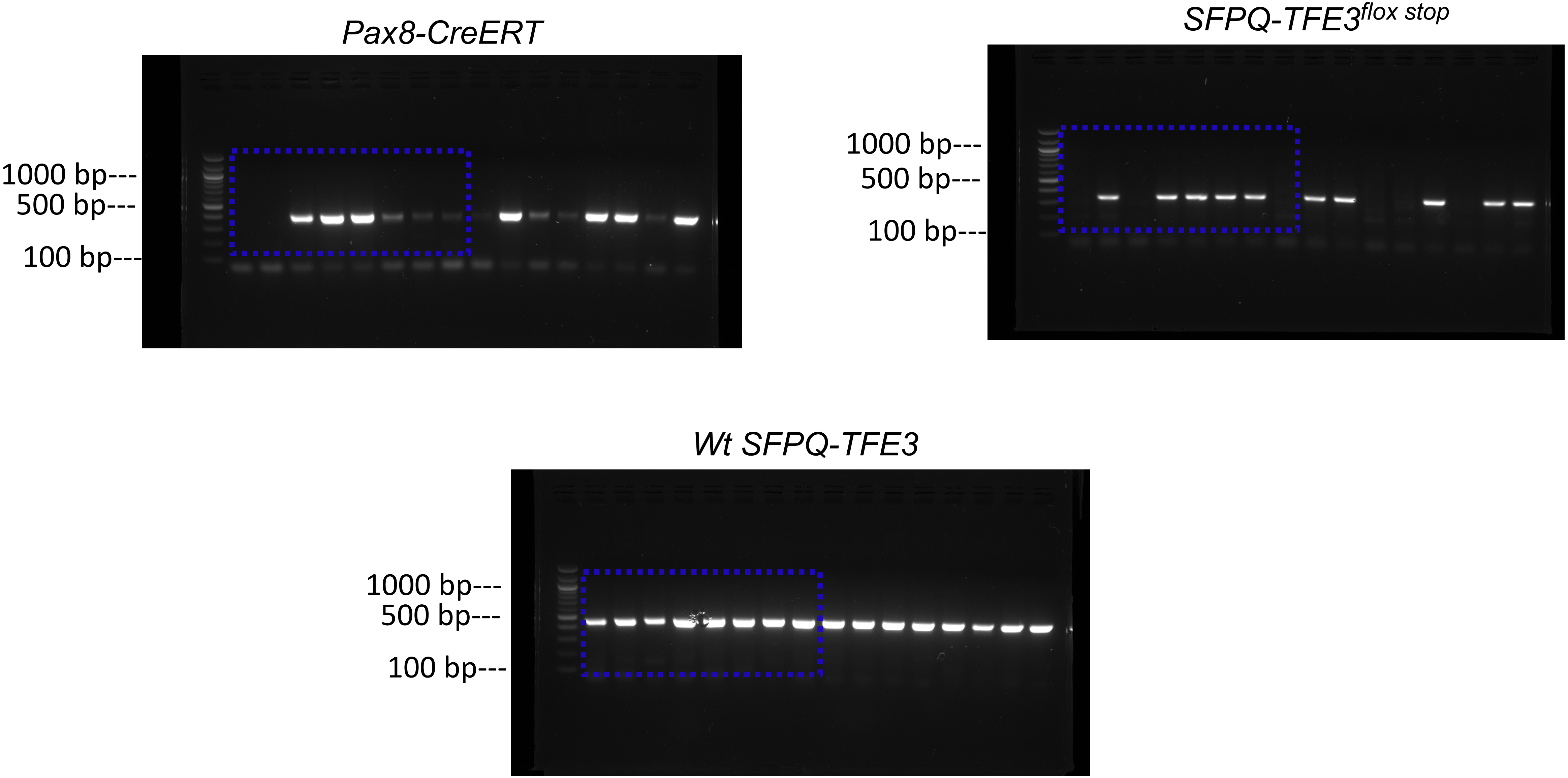

Pax8-CreERT
1000 bp---
500 bp---
100 bp---
SFPQ-TFE3flox stop
1000 bp---
500 bp---
100 bp---
Wt SFPQ-TFE3
1000 bp---
500 bp---
100 bp---

FIGURE S4B

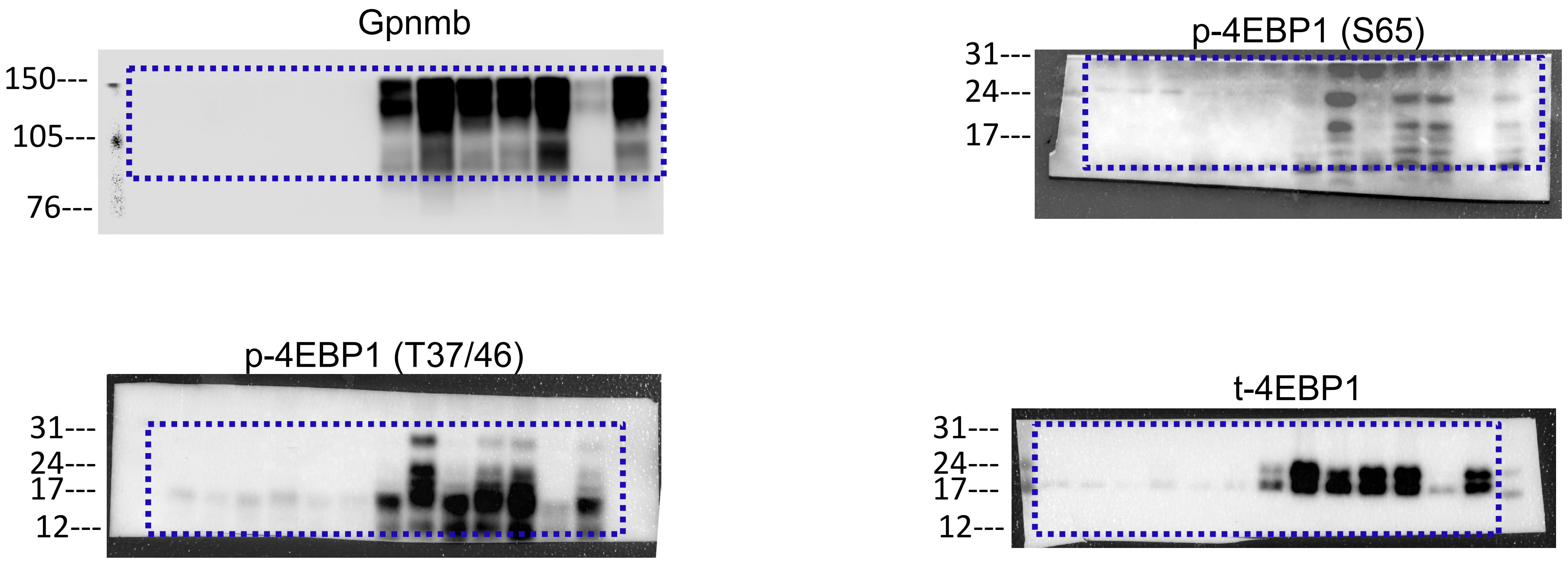
Gpnmb
150---
105---
76---
p-4EBP1 (S65)
31---
24---
17---
p-4EBP1 (T37/46)
31---
24---
17---
12---
t-4EBP1
31---
24---
17---
12---

FIGURE S4B

p-S6 (S6235/236)

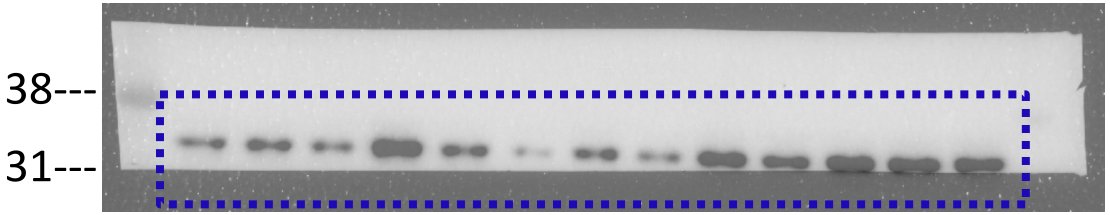

t-S6

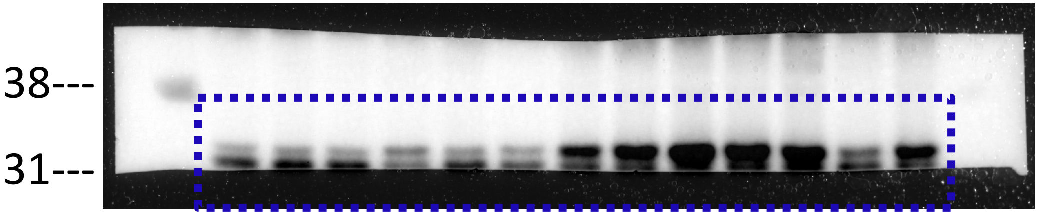

Gapdh

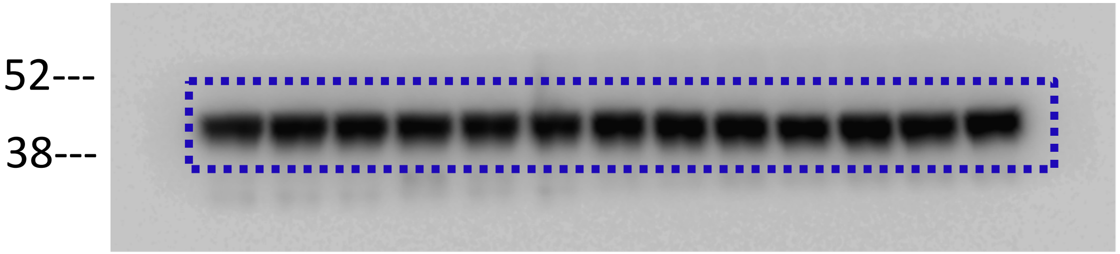

FIGURE S4D

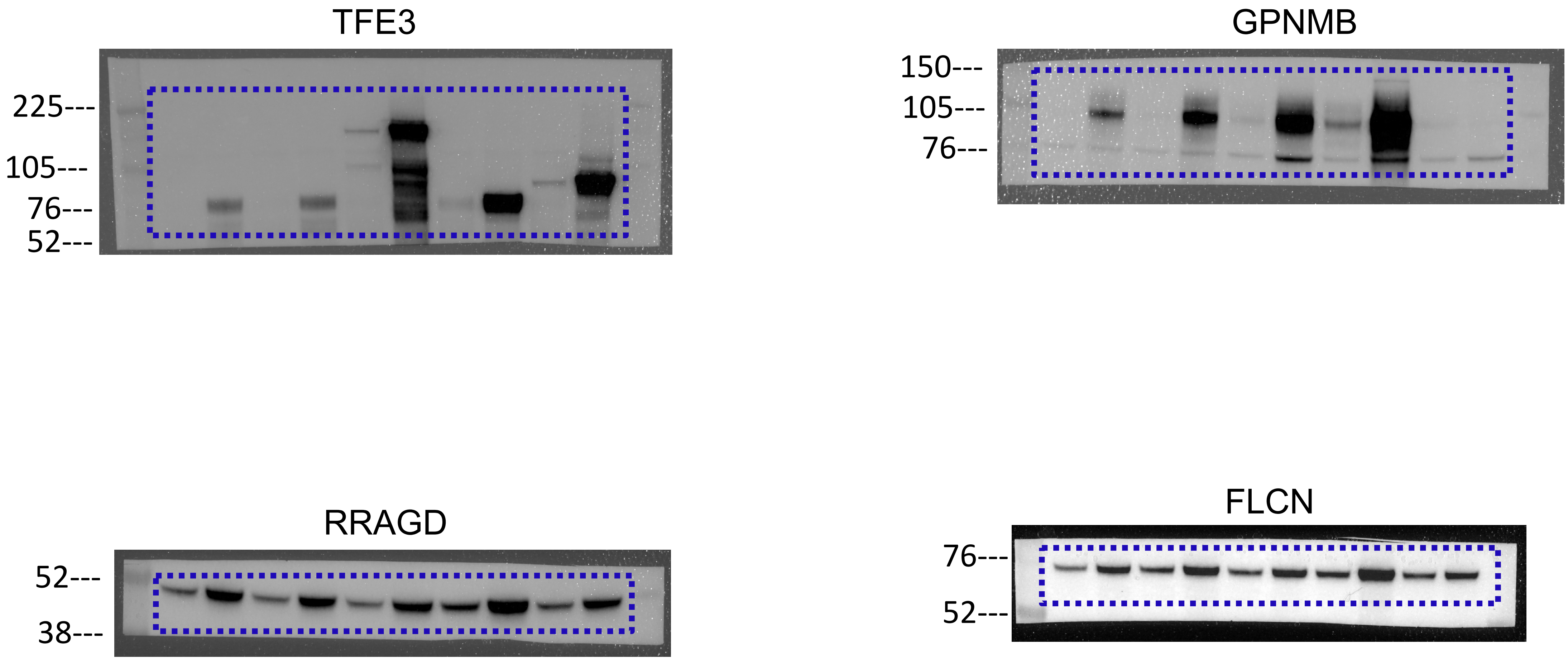

FIGURE S4D

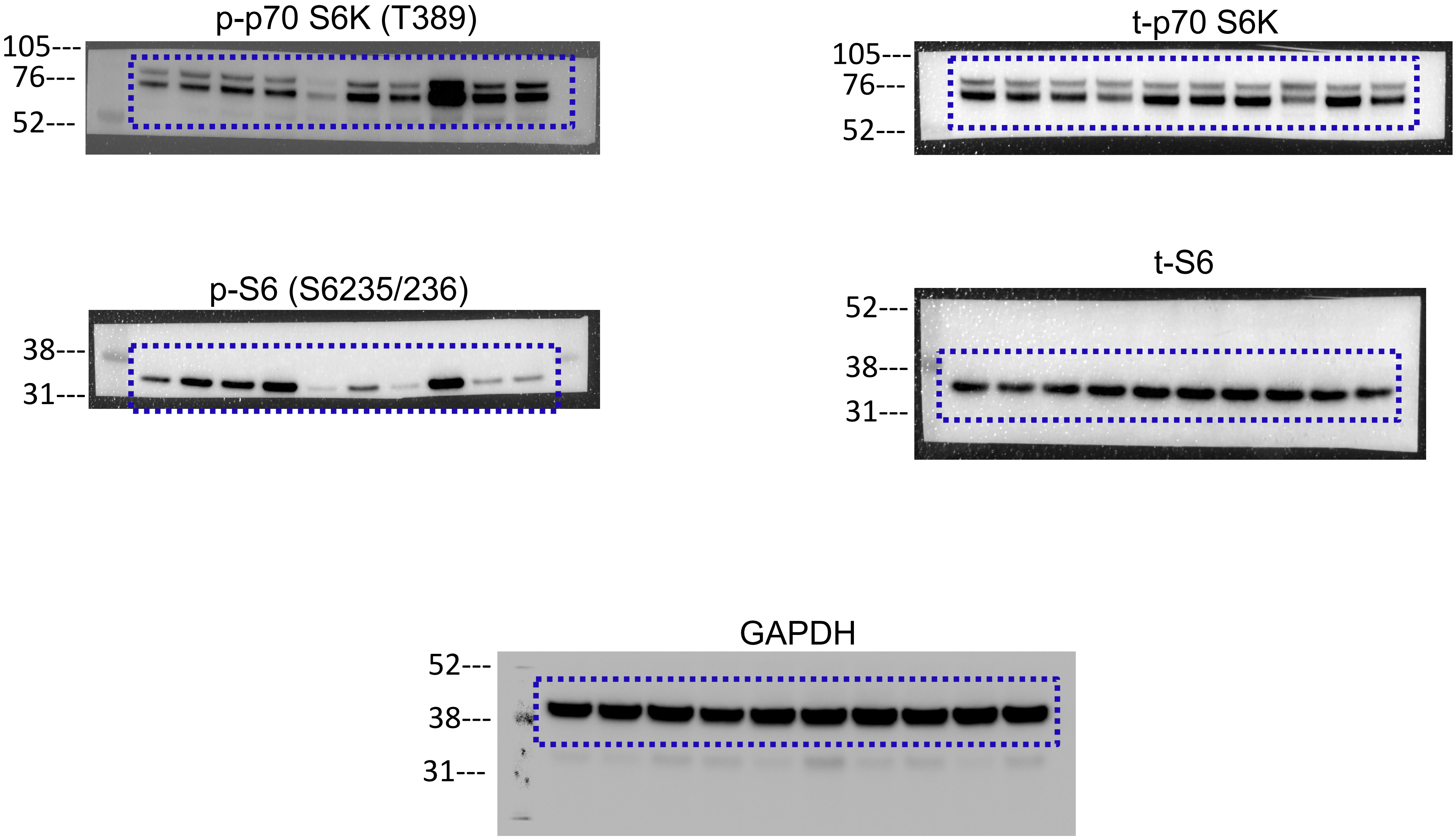
p-p70 S6K (T389)
105---
76---
52---
t-p70 S6K
105---
76---
52---
p-S6 (S6235/236)
38---
31---
t-S6
52---
38---
31---
GAPDH
52---
38---
31---

FIGURE S4E

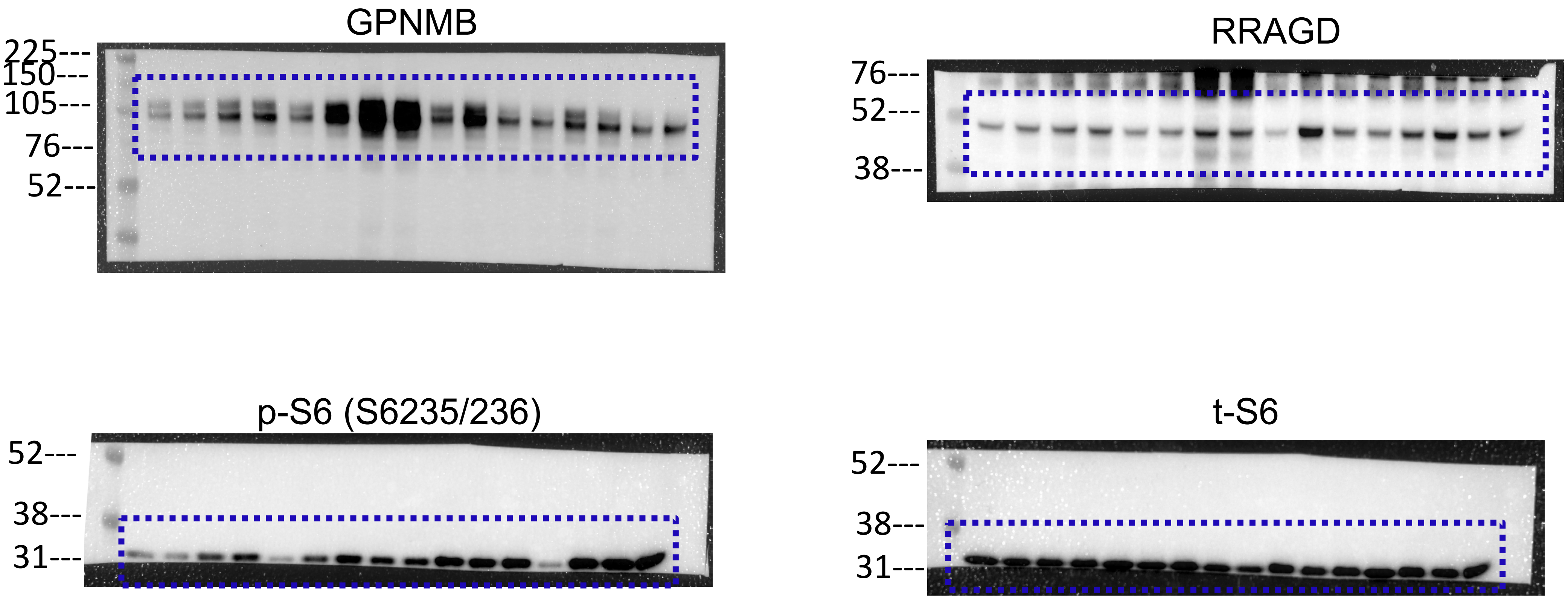
GPNMB
225---
150---
105---
76---
52---
RRAGD
76---
52---
38---
p-S6 (S6235/236)
52---
38---
31---
t-S6
52---
38---
31---

FIGURE S4E

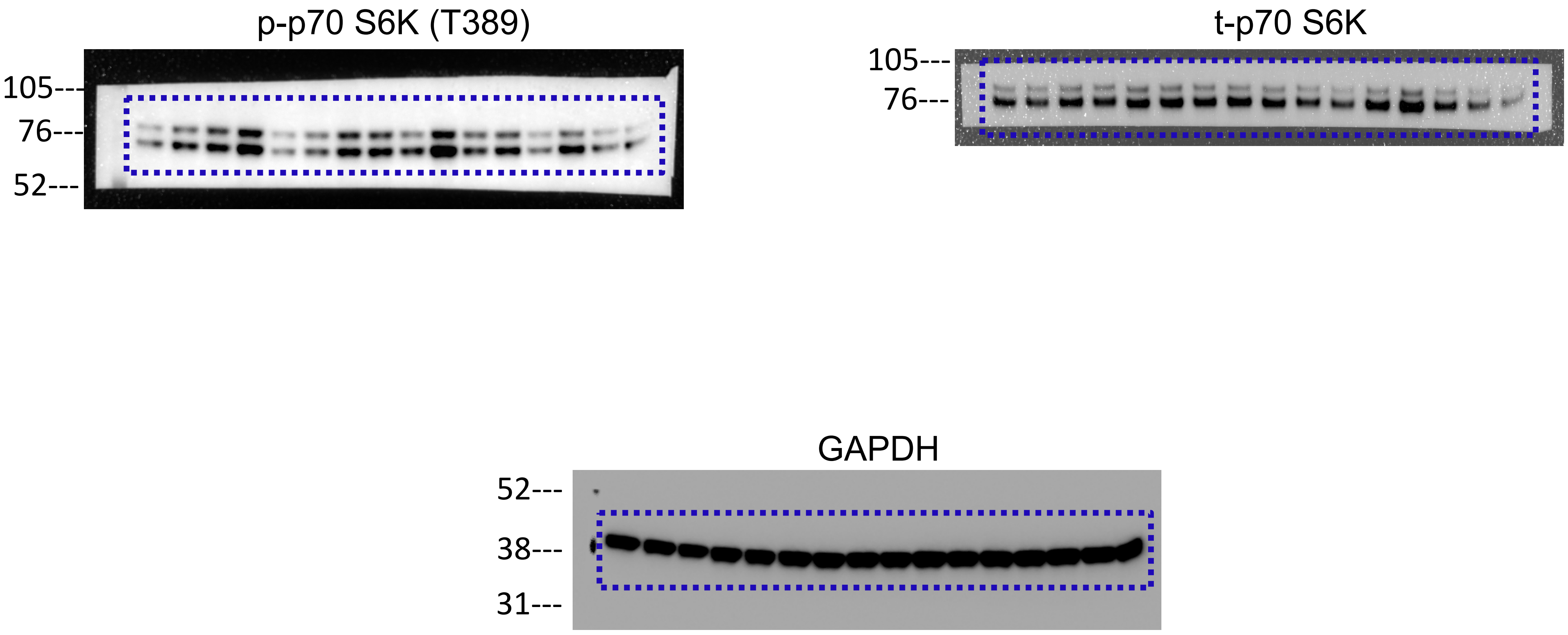

FIGURE S4F

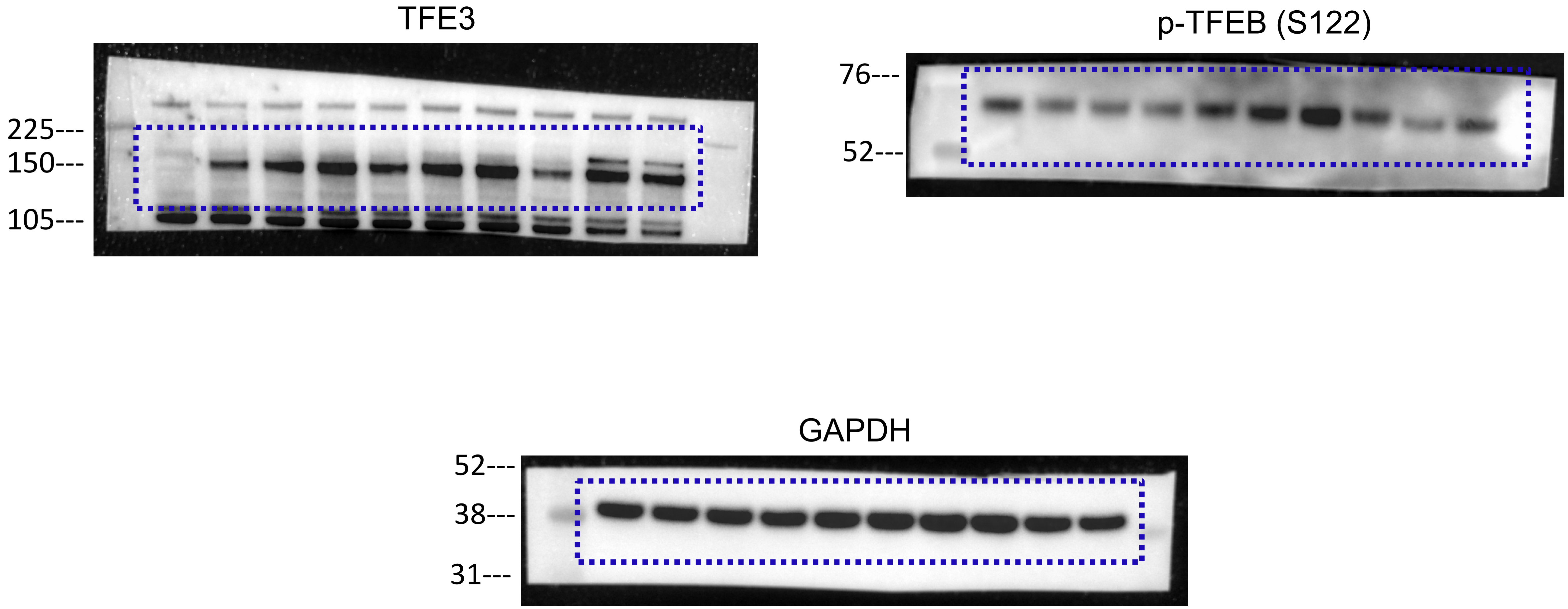

FIGURE S5D

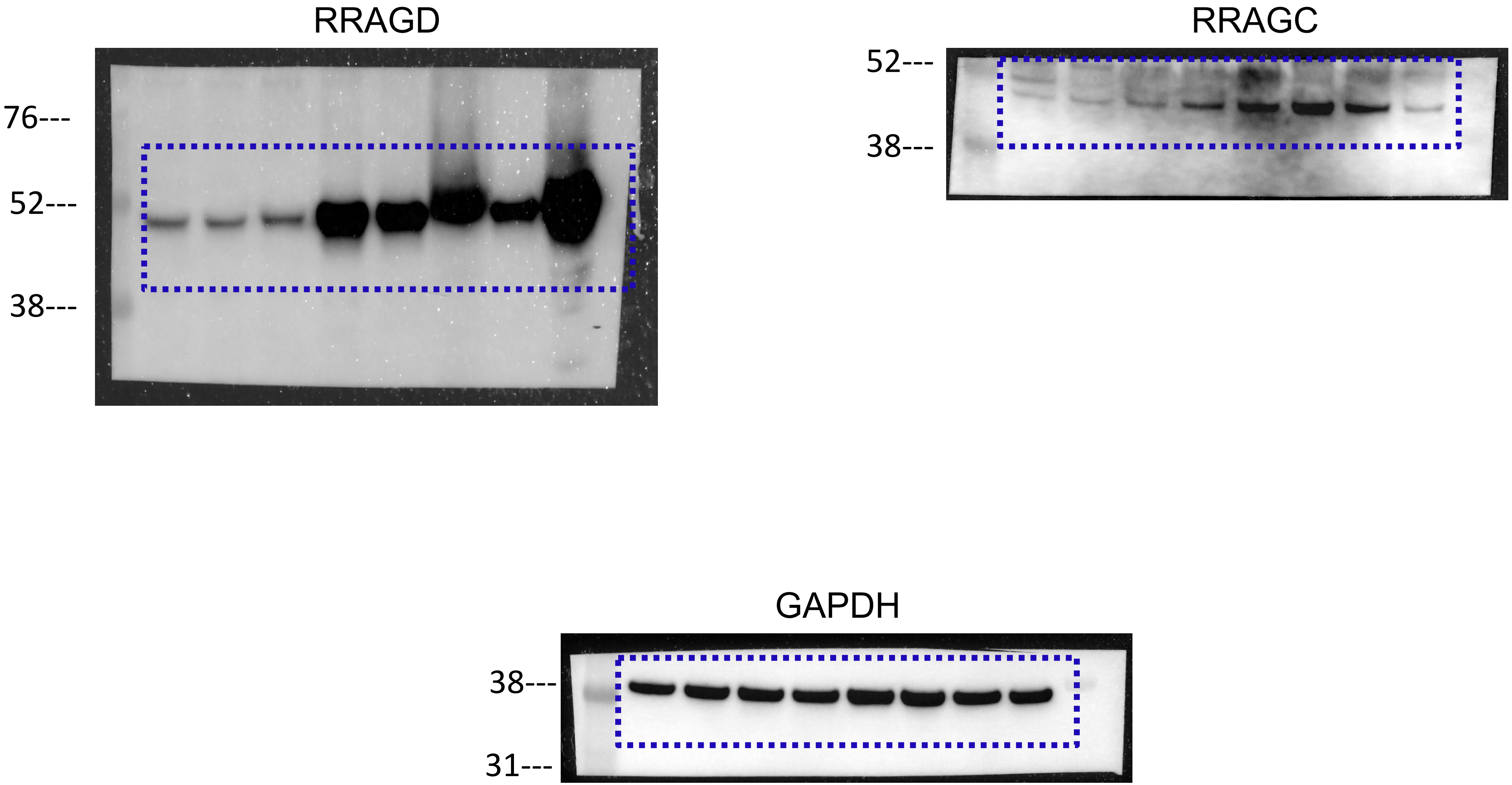

FIGURE S5D

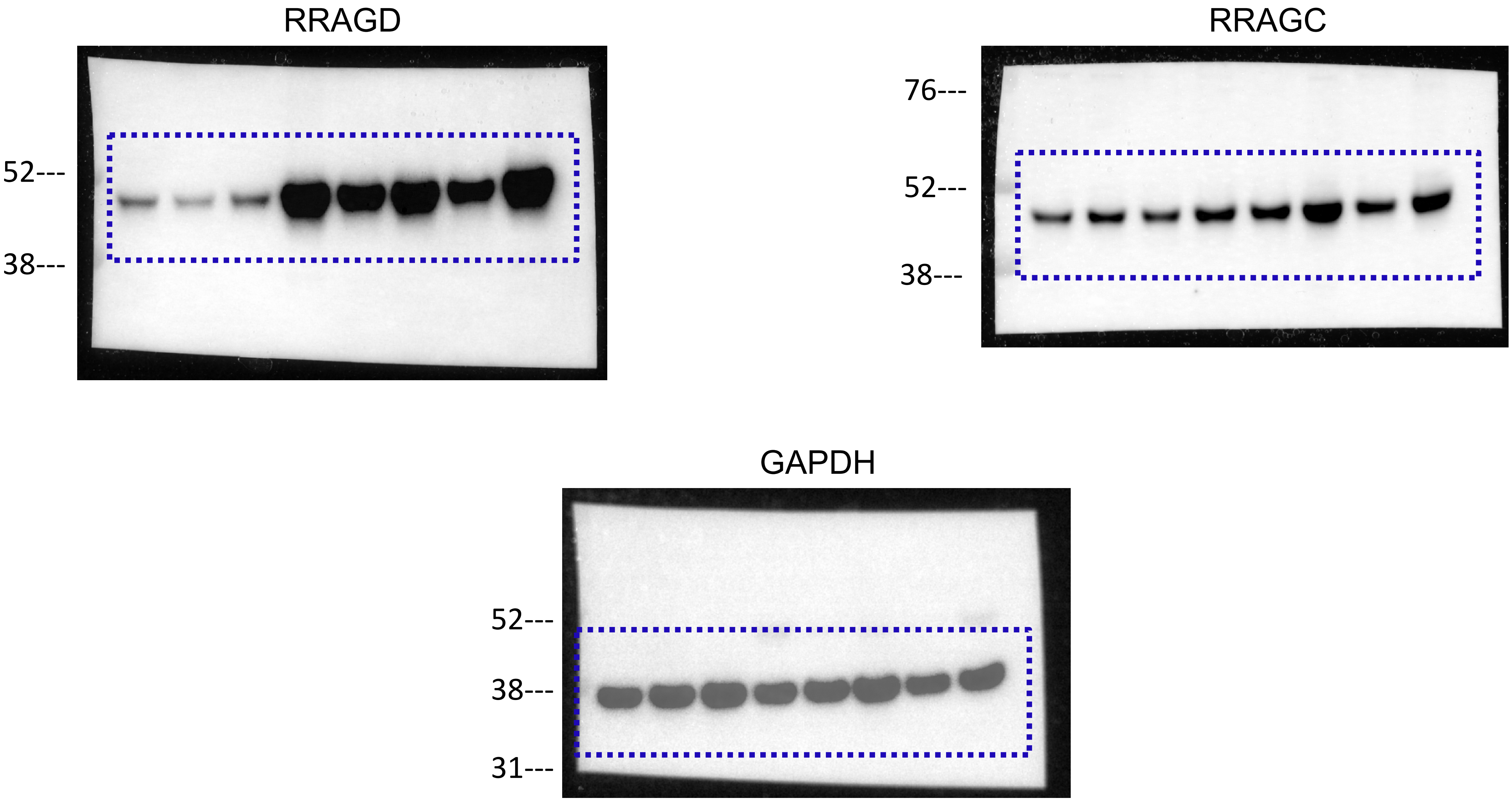

FIGURE S5G

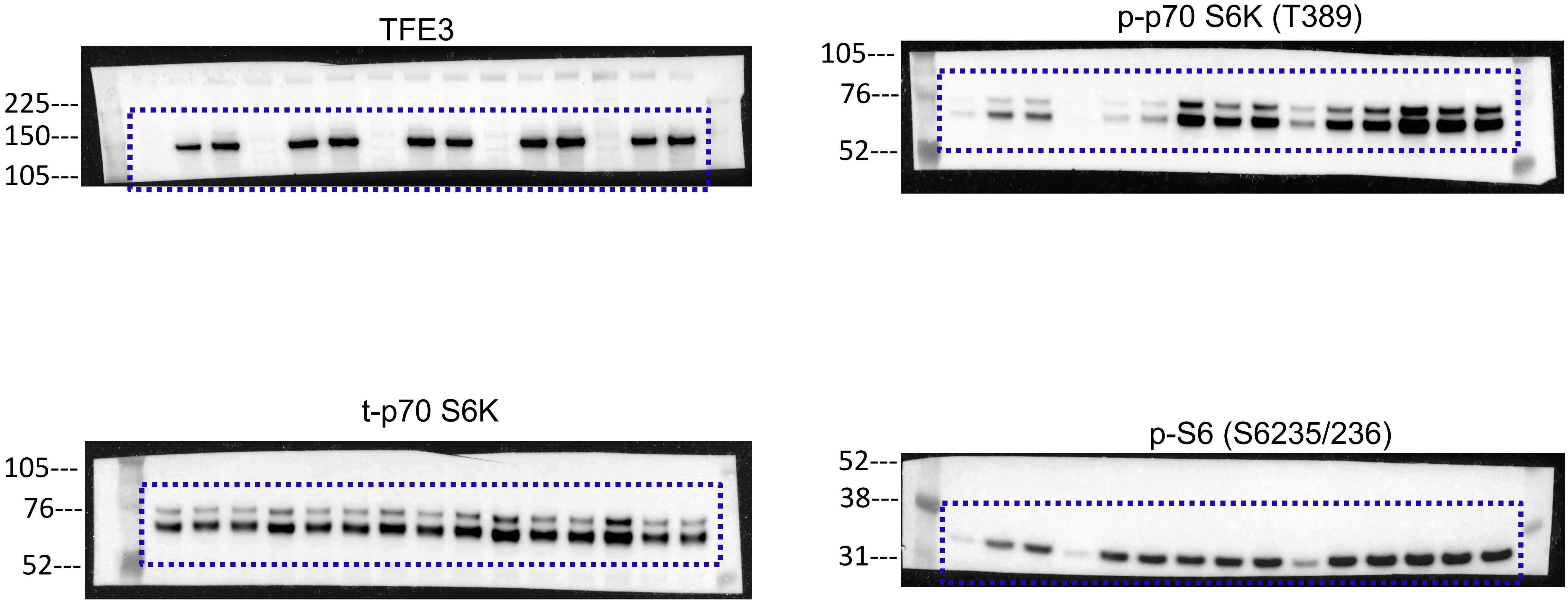
TFE3
225---
150---
105---
p-p70 S6K (T389)
105---
76---
52---
t-p70 S6K
105---
76---
52---
p-S6 (S6235/236)
52---
38---
31---

FIGURE S5G

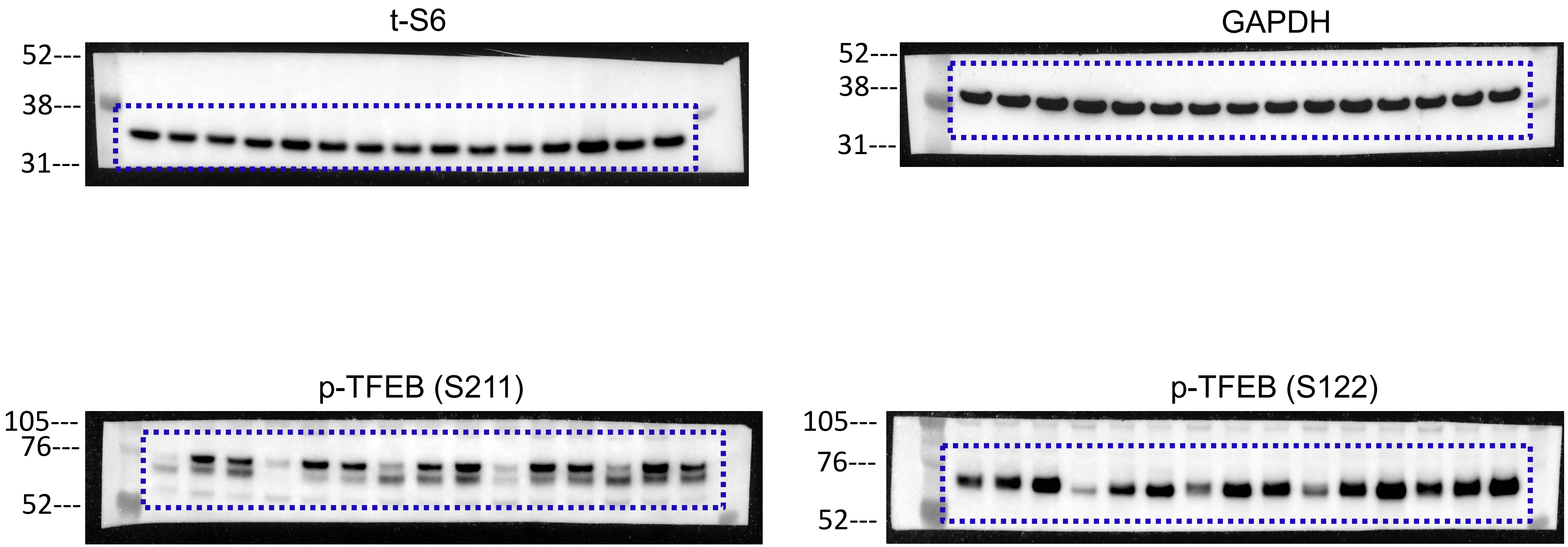

t-S6
52---
38---
31---
GAPDH
52---
38---
31---
p-TFEB (S211)
105---
76---
52---
p-TFEB (S122)
105---
76---
52---

FIGURE S5G

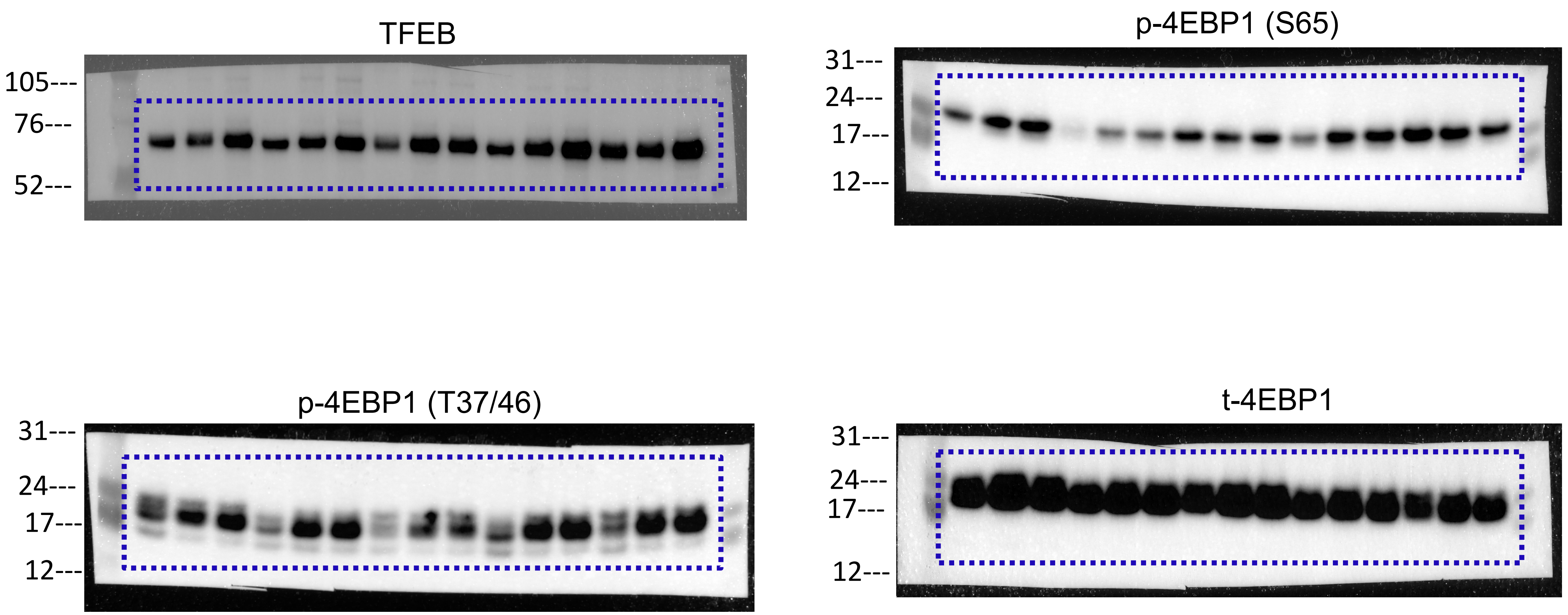
TFEB
105---
76---
52---
p-4EBP1 (S65)
31---
24---
17---
12---
p-4EBP1 (T37/46)
31---
24---
17---
12---
t-4EBP1
31---
24---
17---
12---

FIGURE S5H

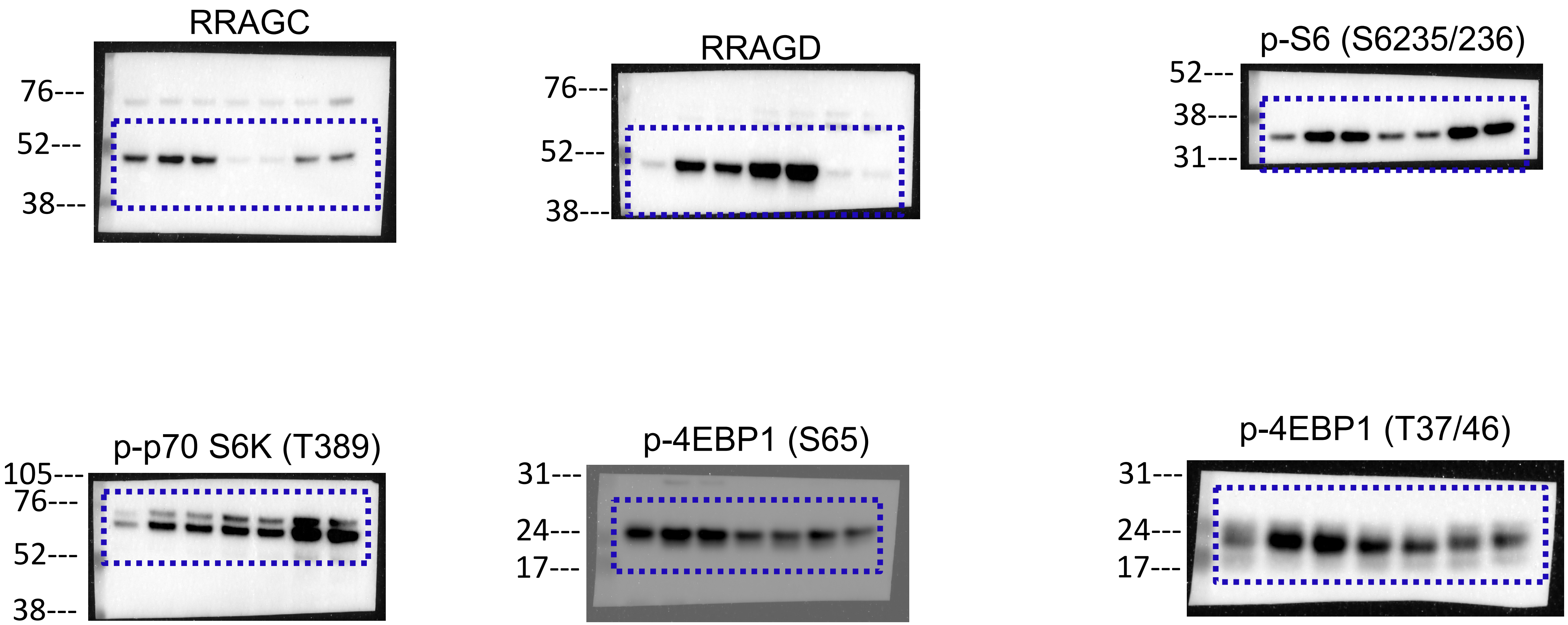
RRAGC
76---
52---
38---
RRAGD
76---
52---
38---
p-S6 (S6235/236)
52---
38---
31---
p-p70 S6K (T389)
105---
76---
52---
38---
p-4EBP1 (S65)
31---
24---
17---
p-4EBP1 (T37/46)
31---
24---
17---

FIGURE S5H

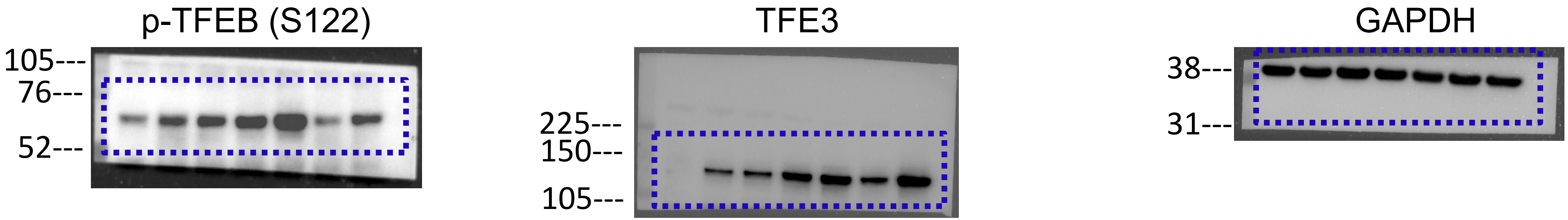

FIGURE S6A

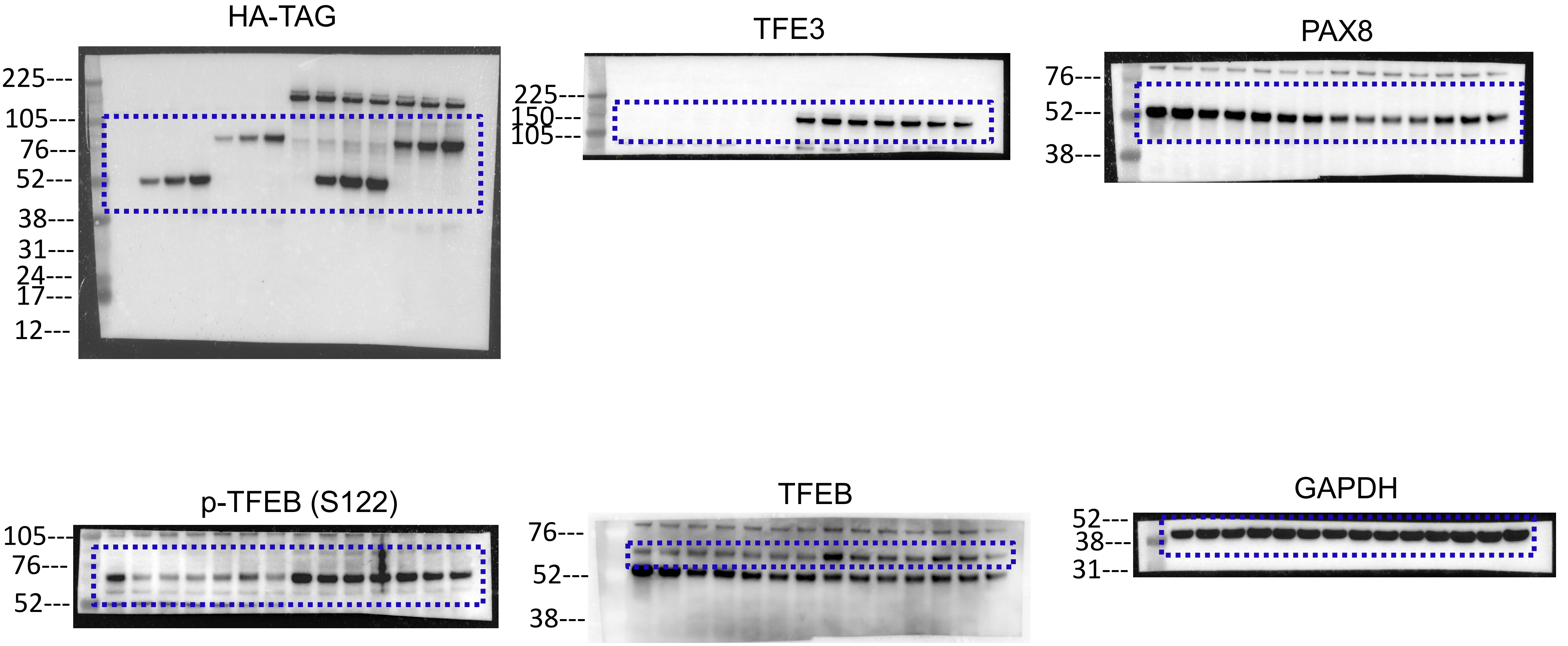
HA-TAG
225---
105---
76---
52---
38---
31---
24---
17---
12---
TFE3
225---
150---
105---
PAX8
76---
52---
38---
p-TFEB (S122)
105---
76---
52---
TFEB
76---
52---
38---
GAPDH
52---
38---
31---

FIGURE S6A

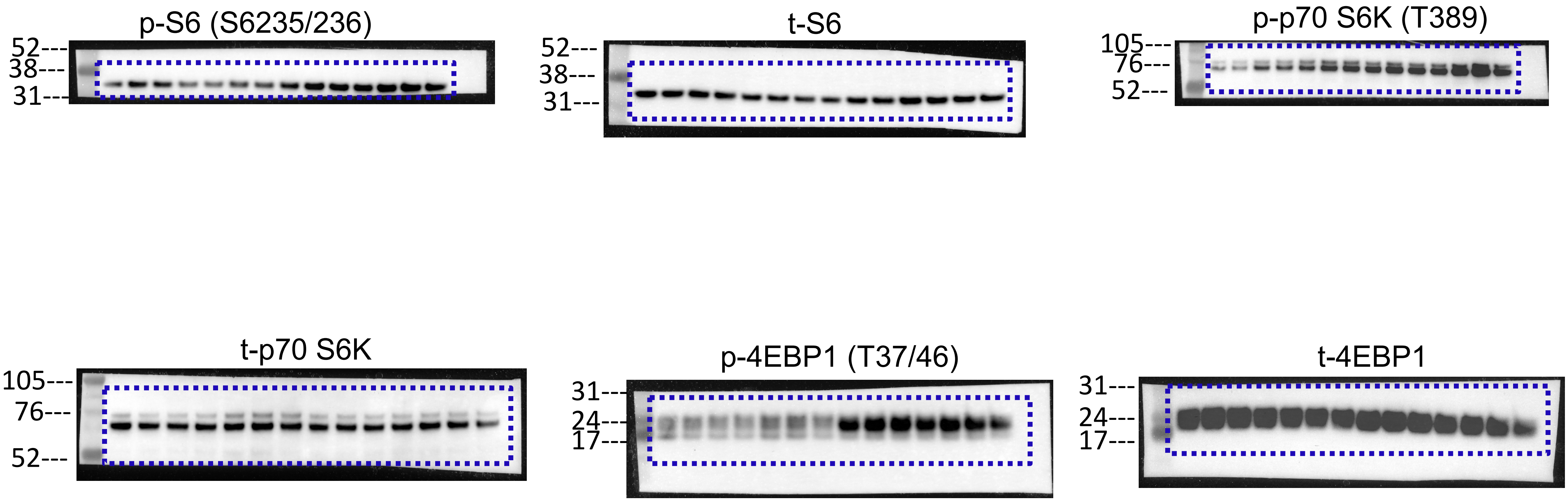
p-S6 (S6235/236)
52---
38---
31---
t-S6
52---
38---
31---
p-p70 S6K (T389)
105---
76---
52---
t-p70 S6K
105---
76---
52---
p-4EBP1 (T37/46)
31---
24---
17---
t-4EBP1
31---
24---
17---

FIGURE S6B (Top)

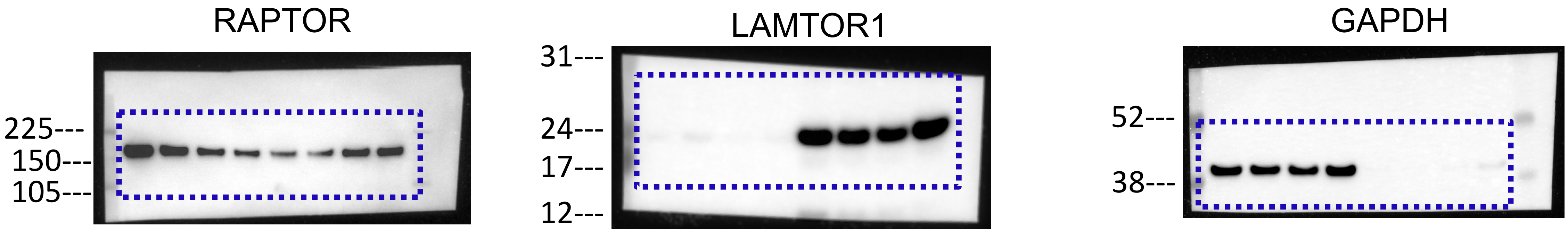

FIGURE S6B (Bottom)

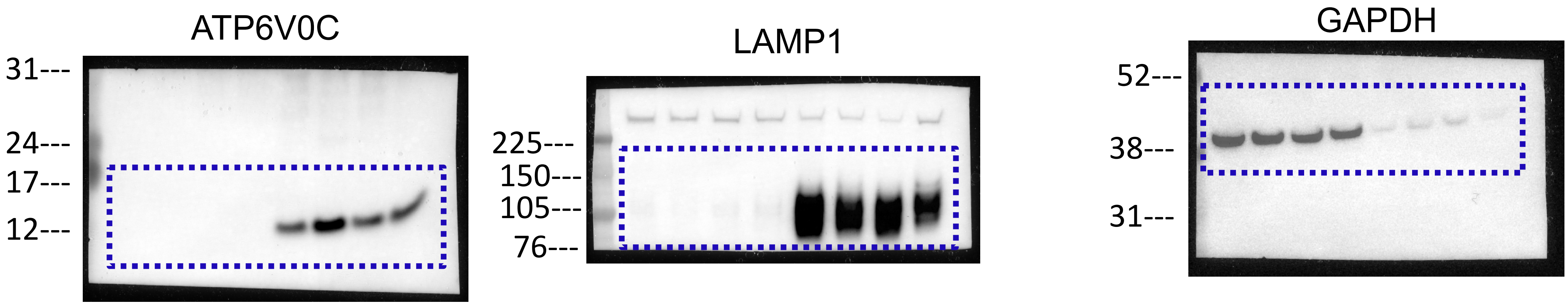

FIGURE S6C (Replicate 1 for quants in S6D)

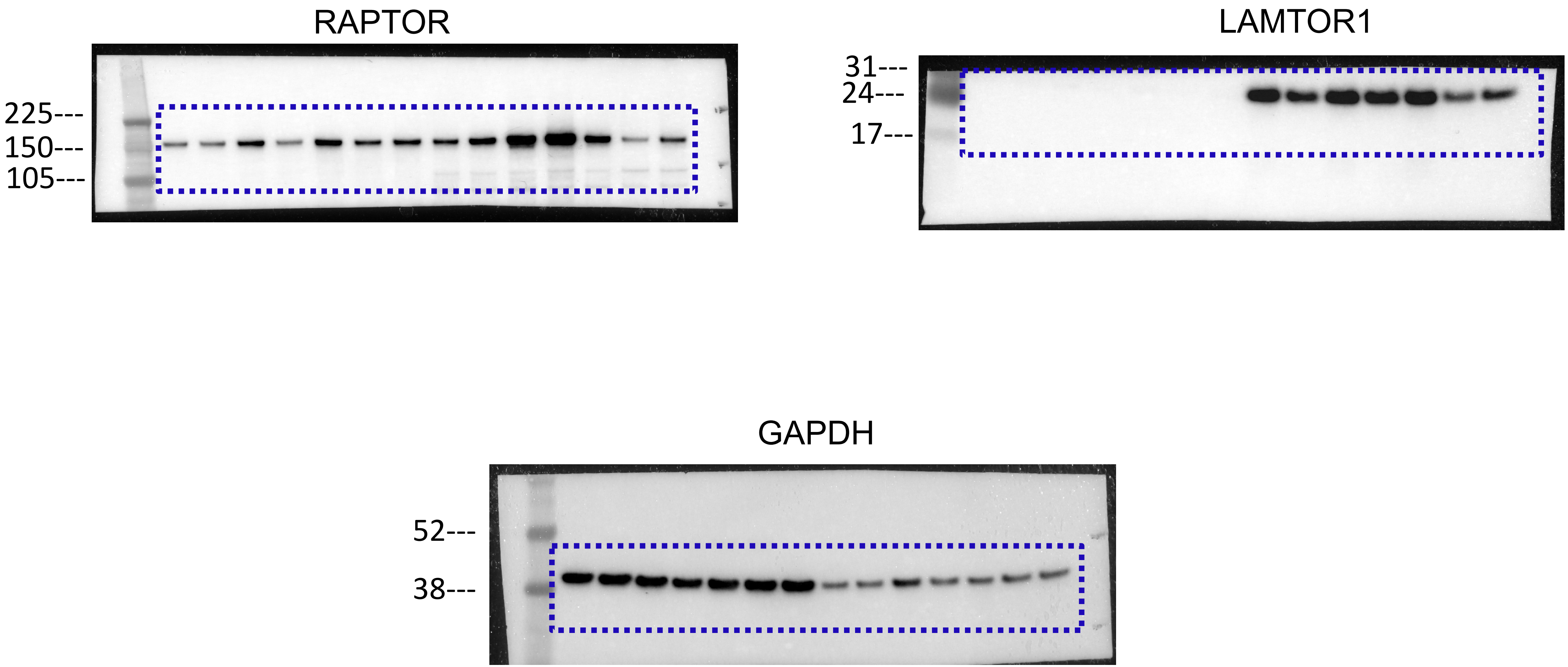

FIGURE S6C (Replicate 2 for quants in S6D)

RAPTOR

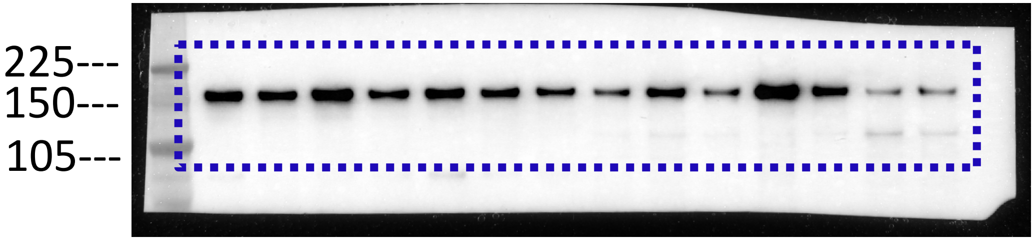

LAMTOR1

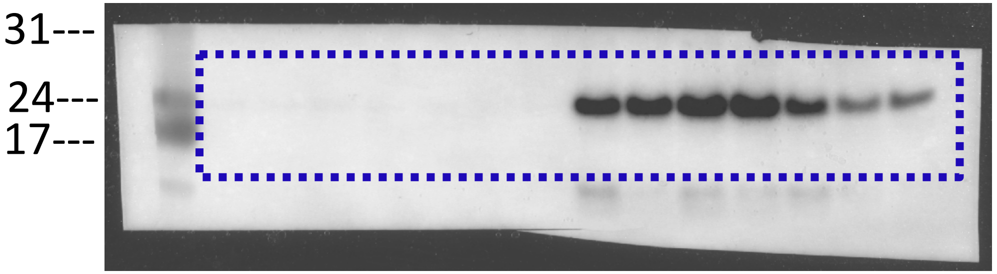

GAPDH

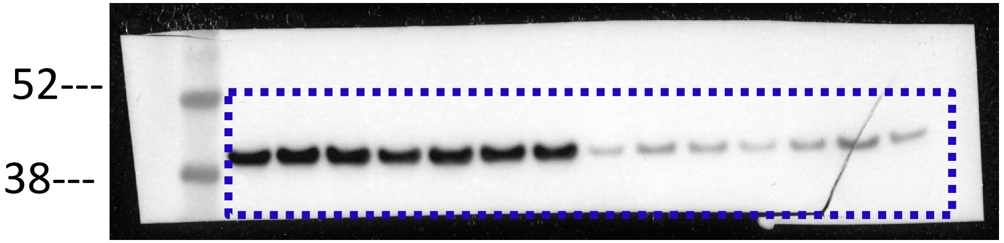

FIGURE S6C (Replicate 3 for quants in S6D)

RAPTOR

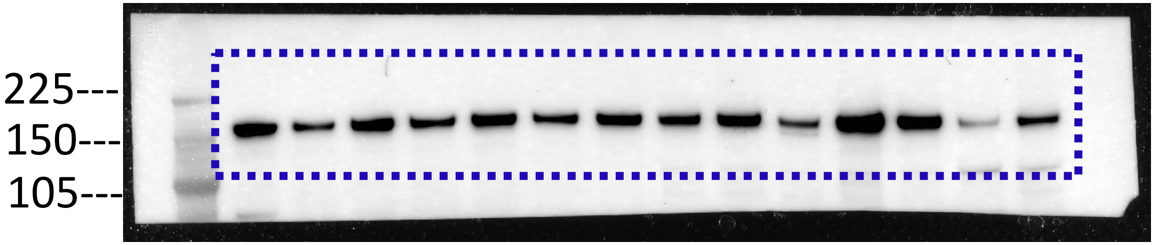

LAMTOR1

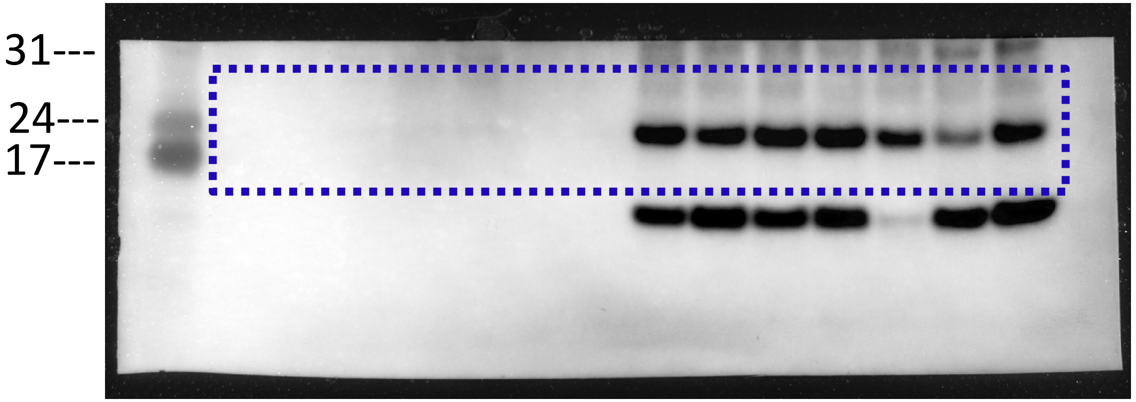

GAPDH

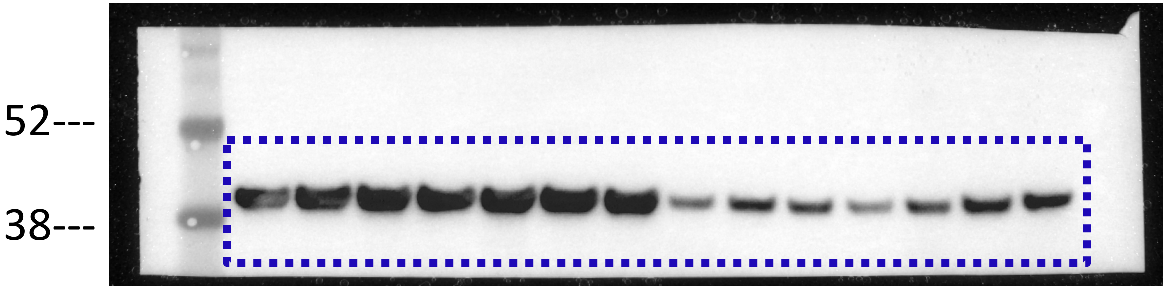

FIGURE S6E

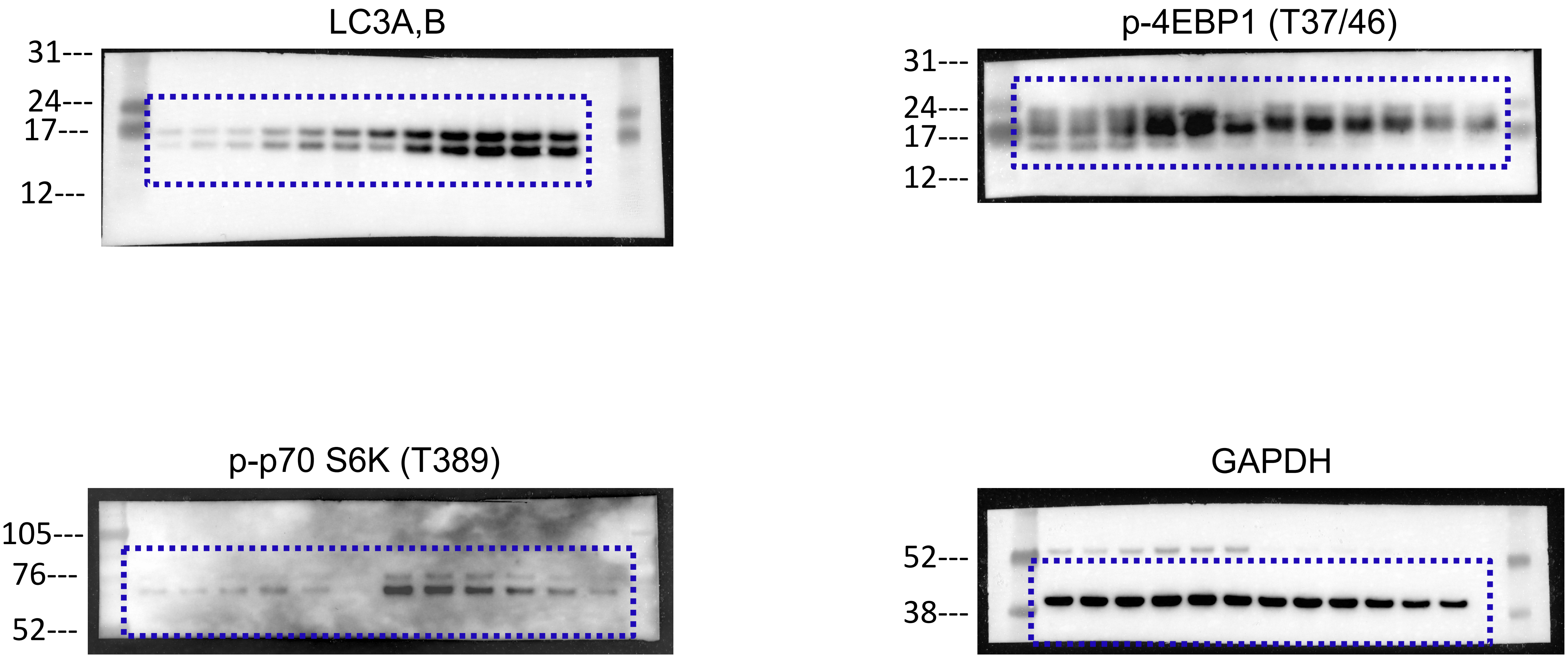

LC3A,B
31---
24---
17---
12---
p-4EBP1 (T37/46)
31---
24---
17---
12---
p-p70 S6K (T389)
105---
76---
52---
GAPDH
52---
38---

FIGURE S8B

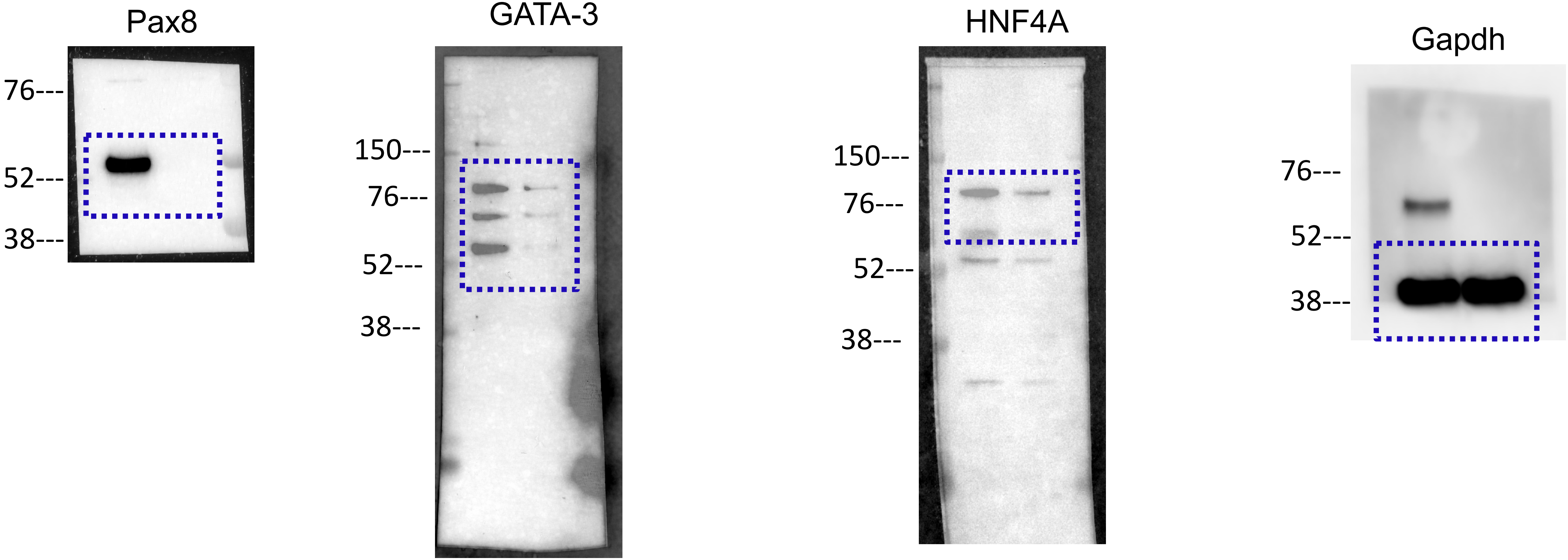

FIGURE S8E

GATA-3

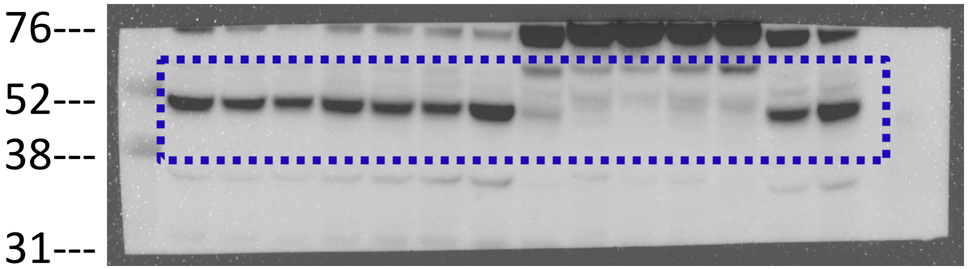

Pan-Keratin (Type1)

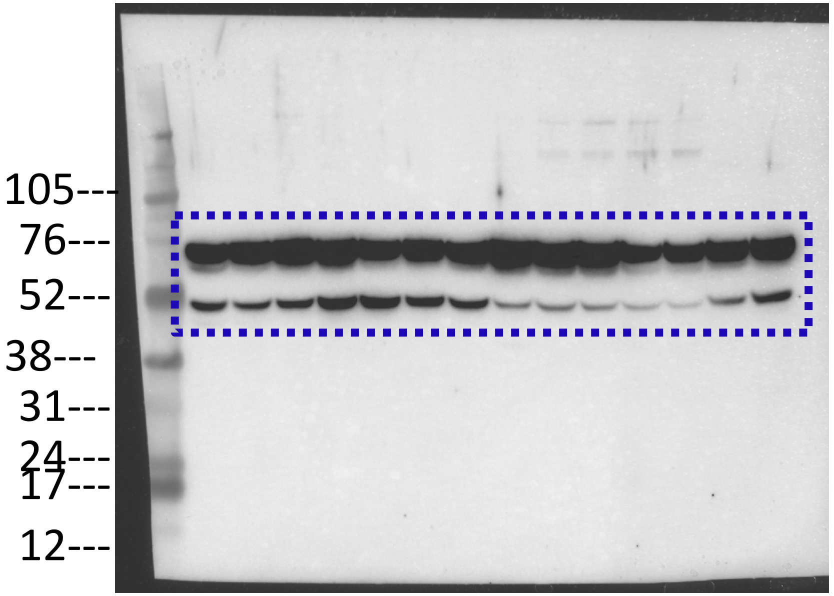

Gapdh

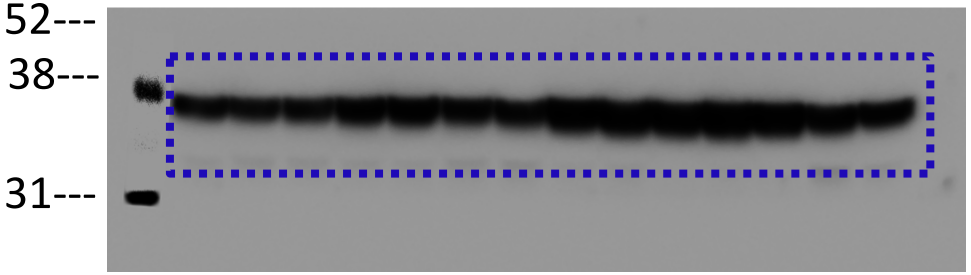

Gapdh

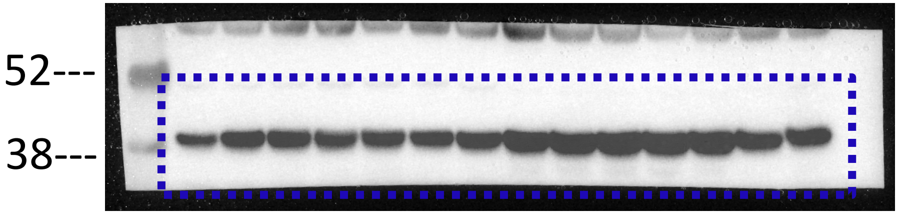

FIGURE S8F

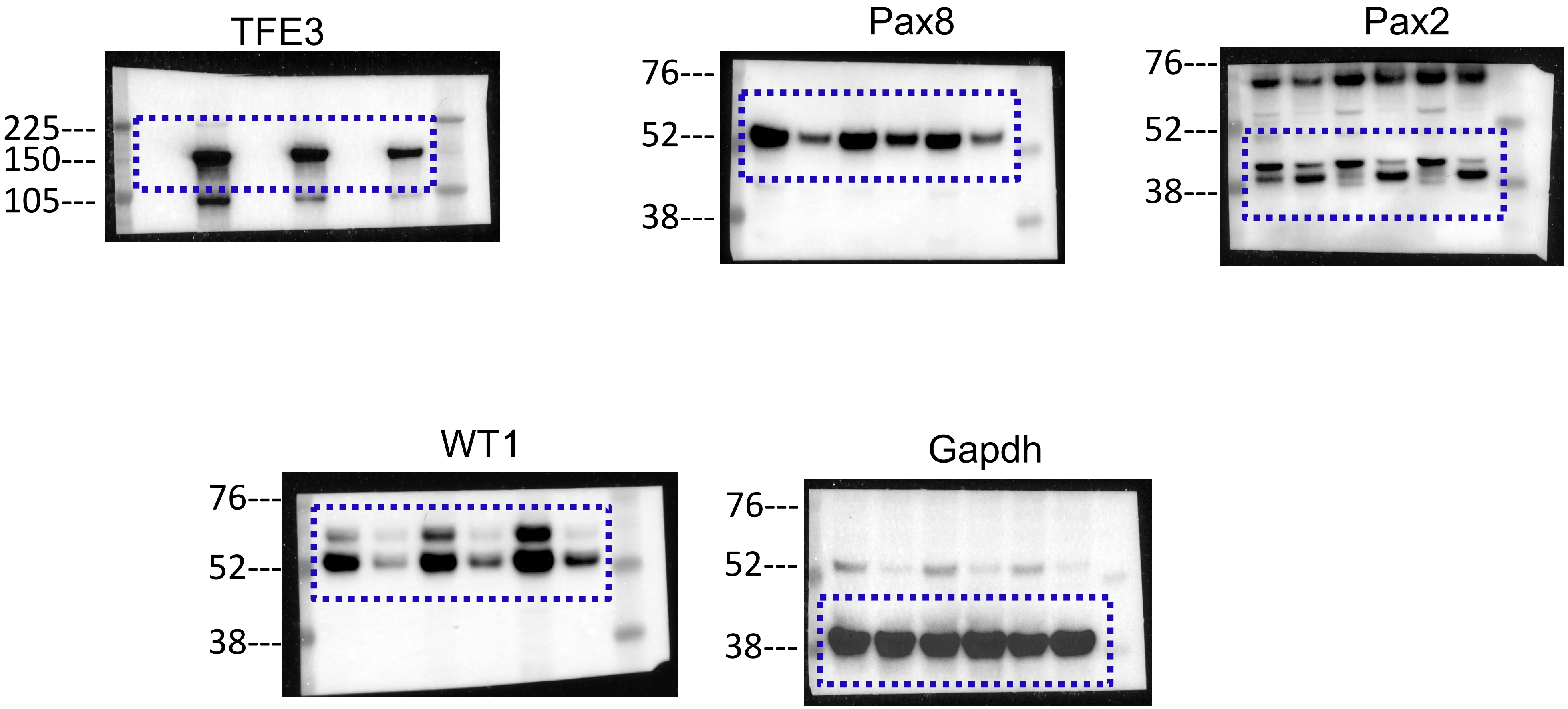

TFE3
225---
150---
105---
Pax8
76---
52---
38---
Pax2
76---
52---
38---
WT1
76---
52---
38---
Gapdh
76---
52---
38---

FIGURE S9A

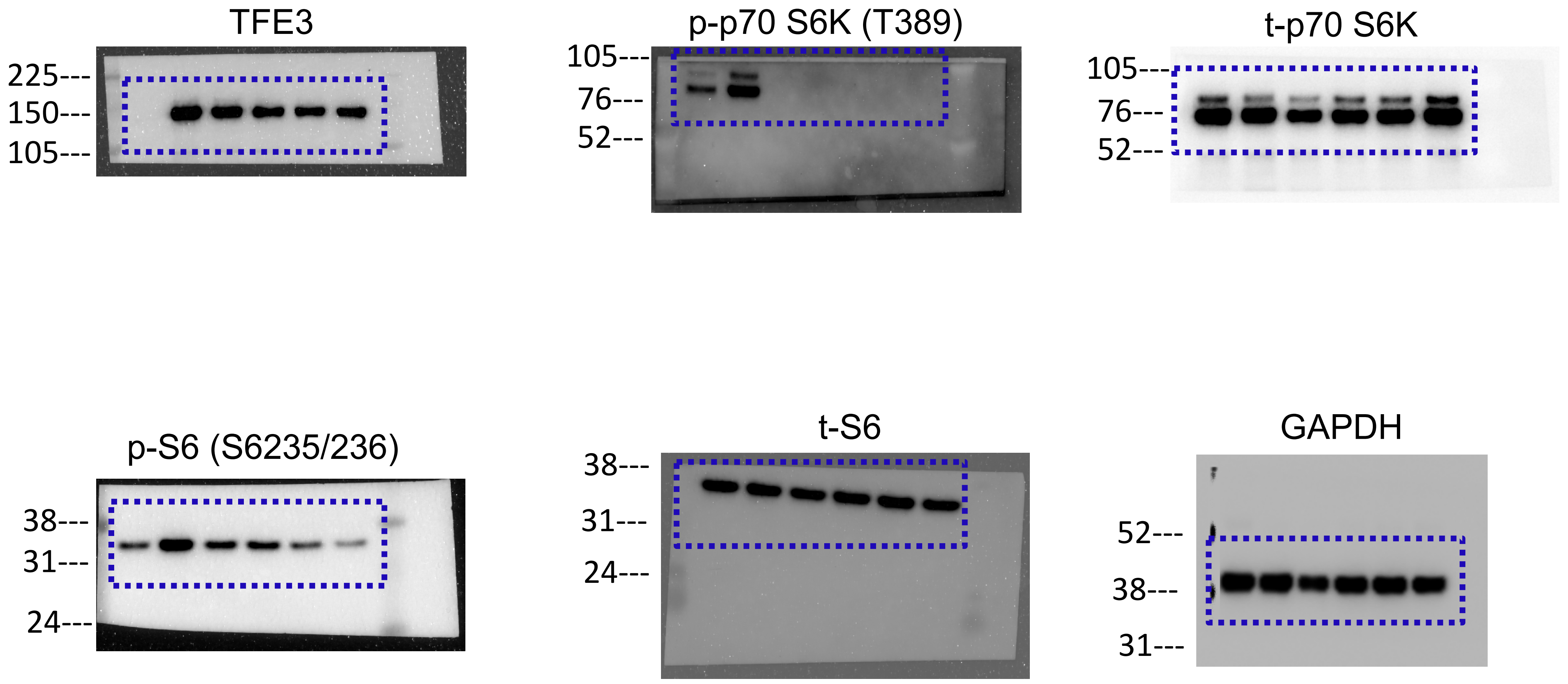
TFE3
225---
150---
105---
p-p70 S6K (T389)
105---
76---
52---
t-p70 S6K
105---
76---
52---
p-S6 (S6235/236)
38---
31---
24---
t-S6
38---
31---
24---
GAPDH
52---
38---
31---

FIGURE S9A

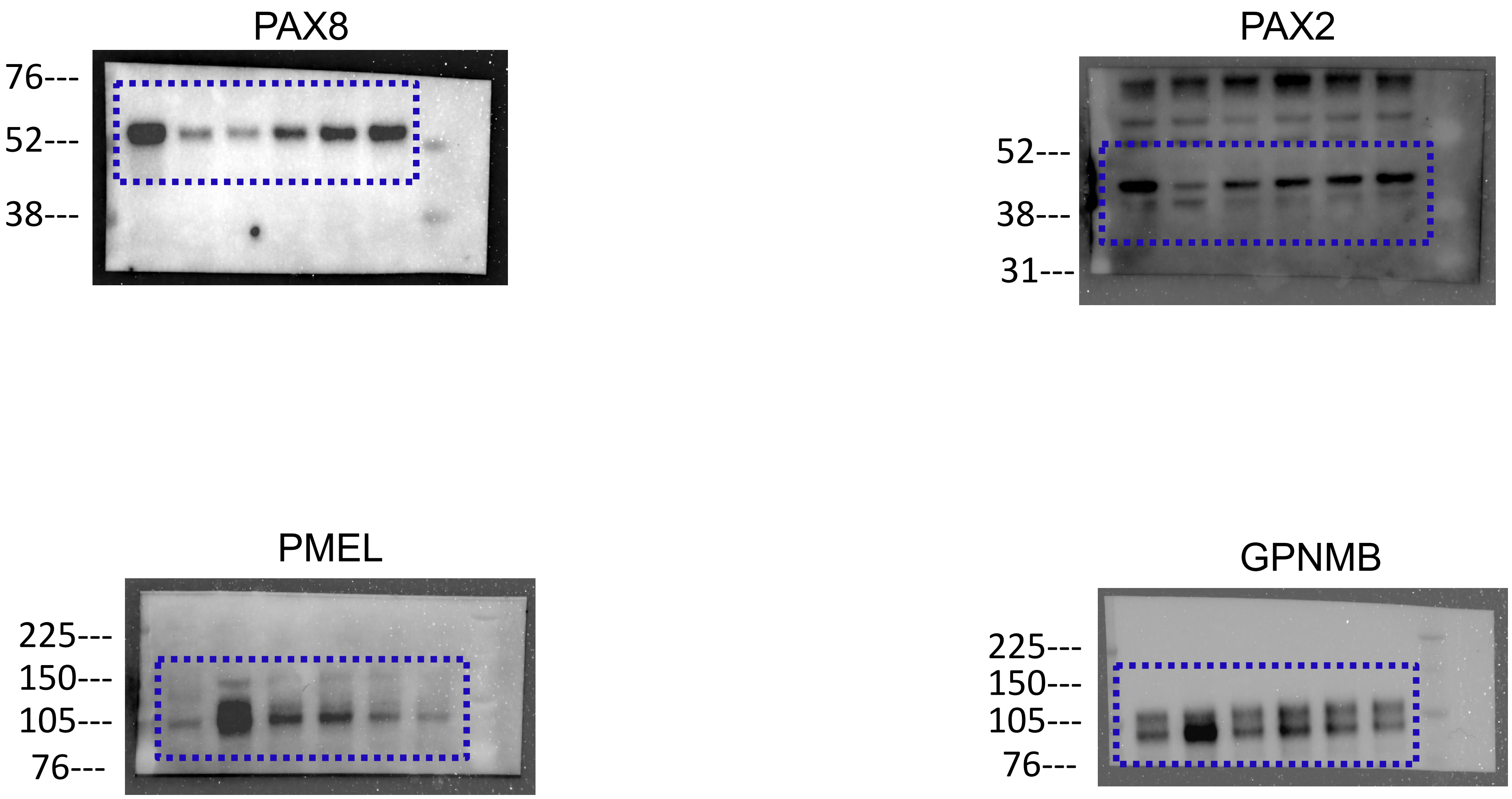
PAX8
76---
52---
38---
PAX2
52---
38---
31---
PMEL
225---
150---
105---
76---
GPNMB
225---
150---
105---
76---

FIGURE S9B

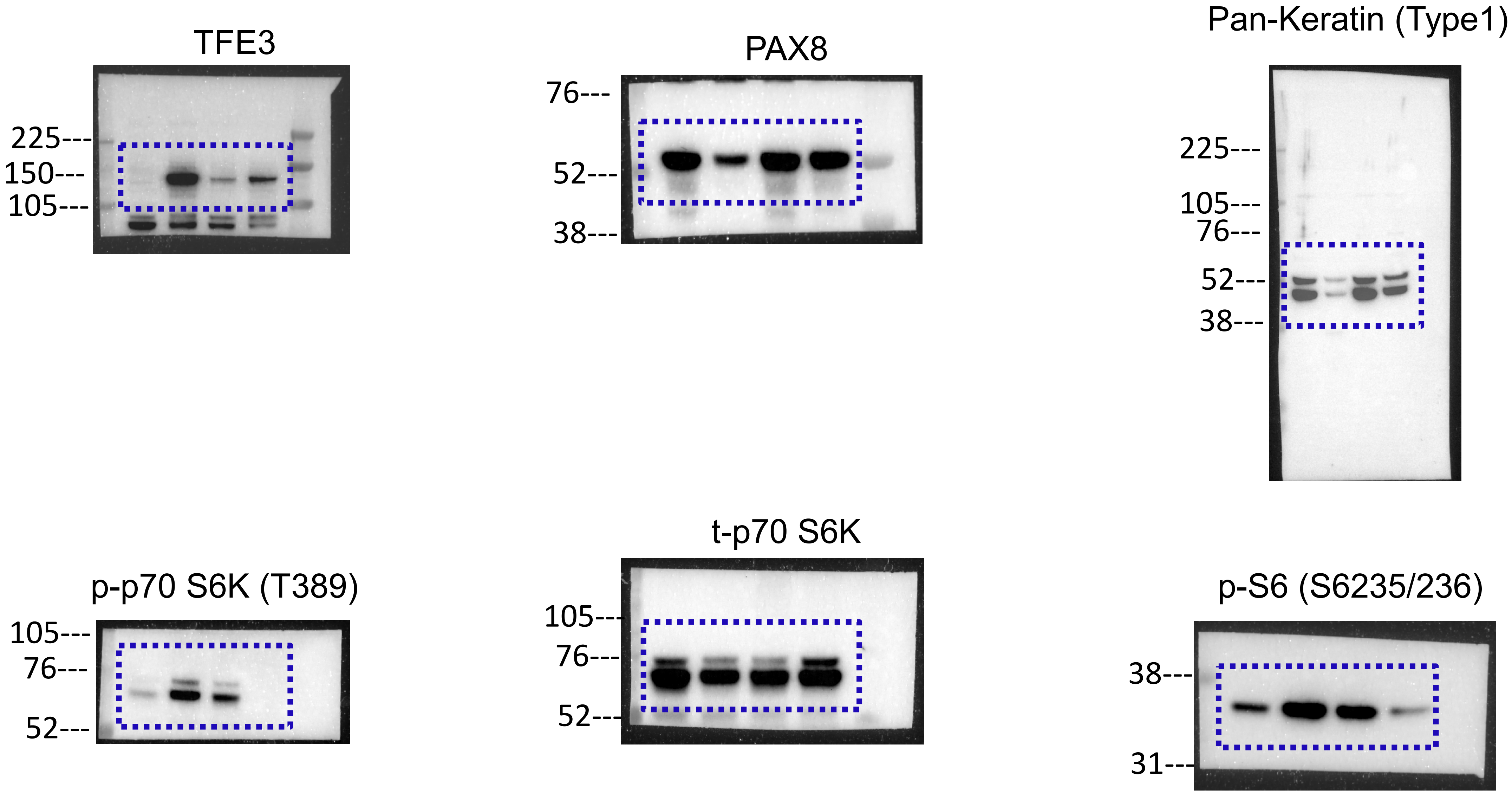
TFE3
225---
150---
105---
PAX8
76---
52---
38---
Pan-Keratin (Type1)
225---
105---
76---
52---
38---
p-p70 S6K (T389)
105---
76---
52---
t-p70 S6K
105---
76---
52---
p-S6 (S6235/236)
38---
31---

FIGURE S9B

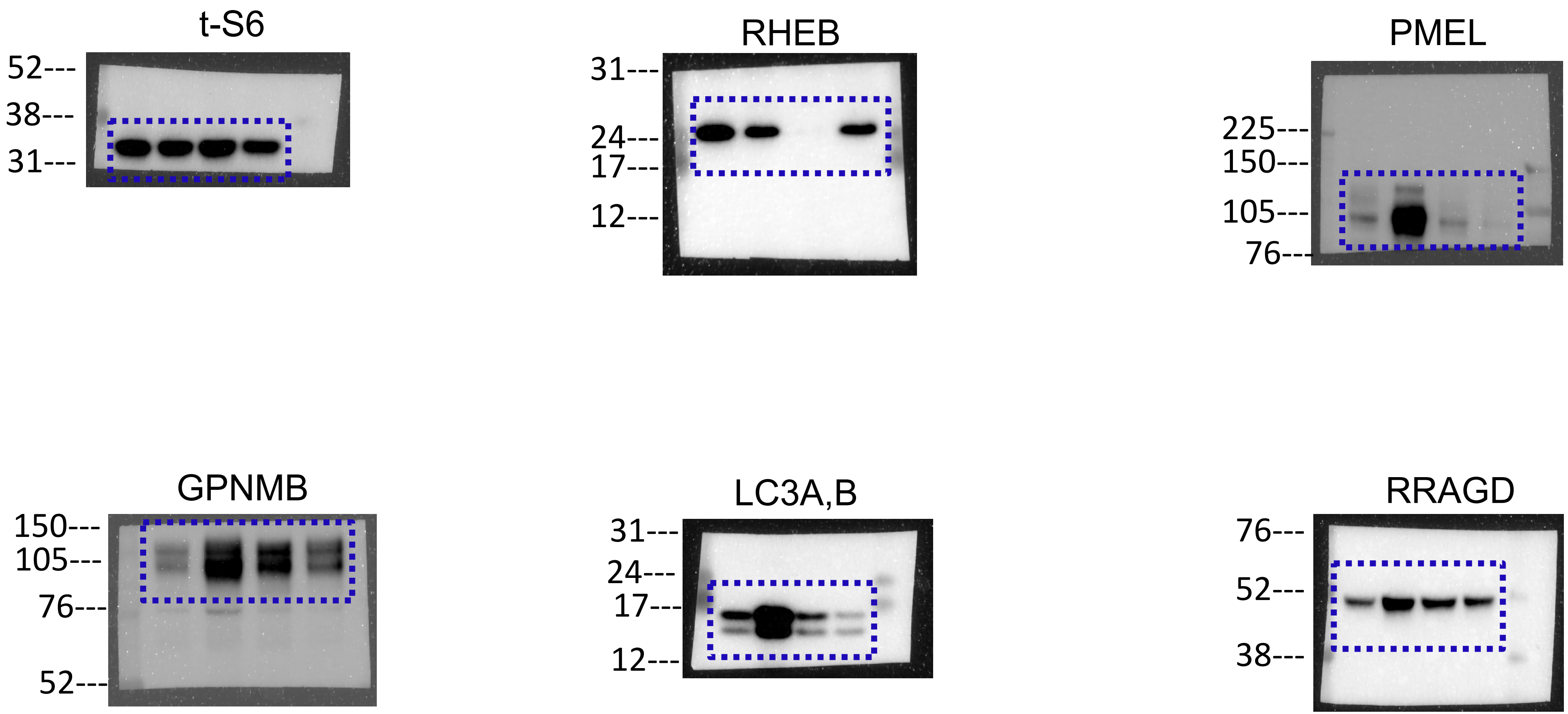
t-S6
52---
38---
31---
RHEB
31---
24---
17---
12---
PMEL
225---
150---
105---
76---
GPNMB
150---
105---
76---
52---
LC3A,B
31---
24---
17---
12---
RRAGD
76---
52---
38---

FIGURE S9B

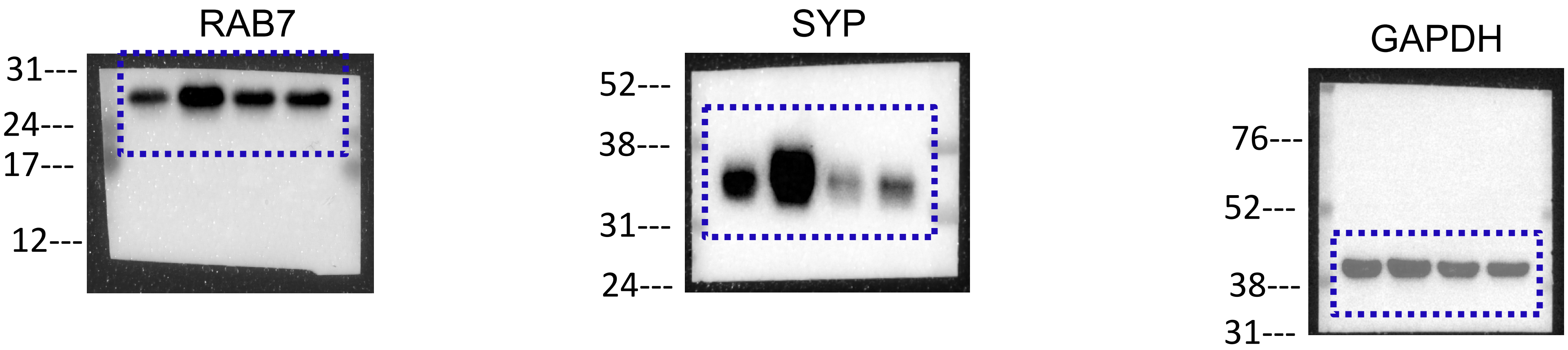

FIGURE S9D

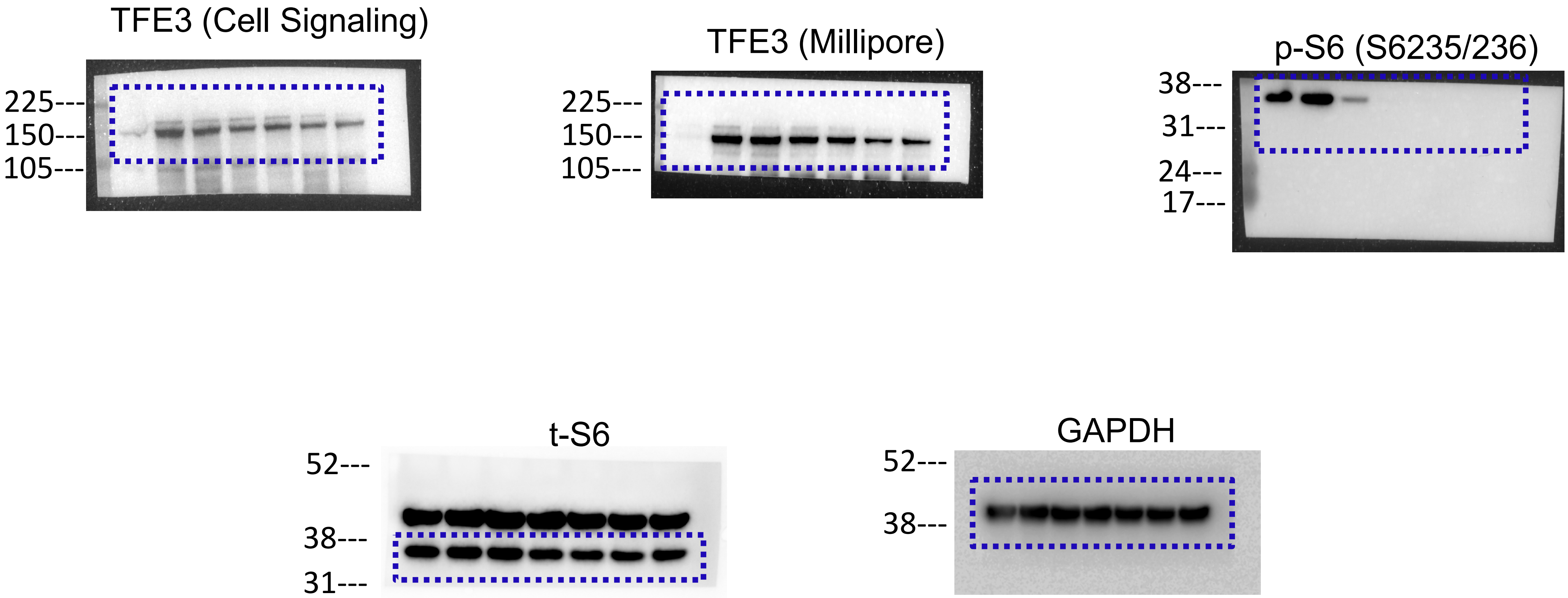
TFE3 (Cell Signaling)
225---
150---
105---
TFE3 (Millipore)
225---
150---
105---
p-S6 (S6235/236)
38---
31---
24---
17---
t-S6
52---
38---
31---
GAPDH
52---
38---

FIGURE S9E

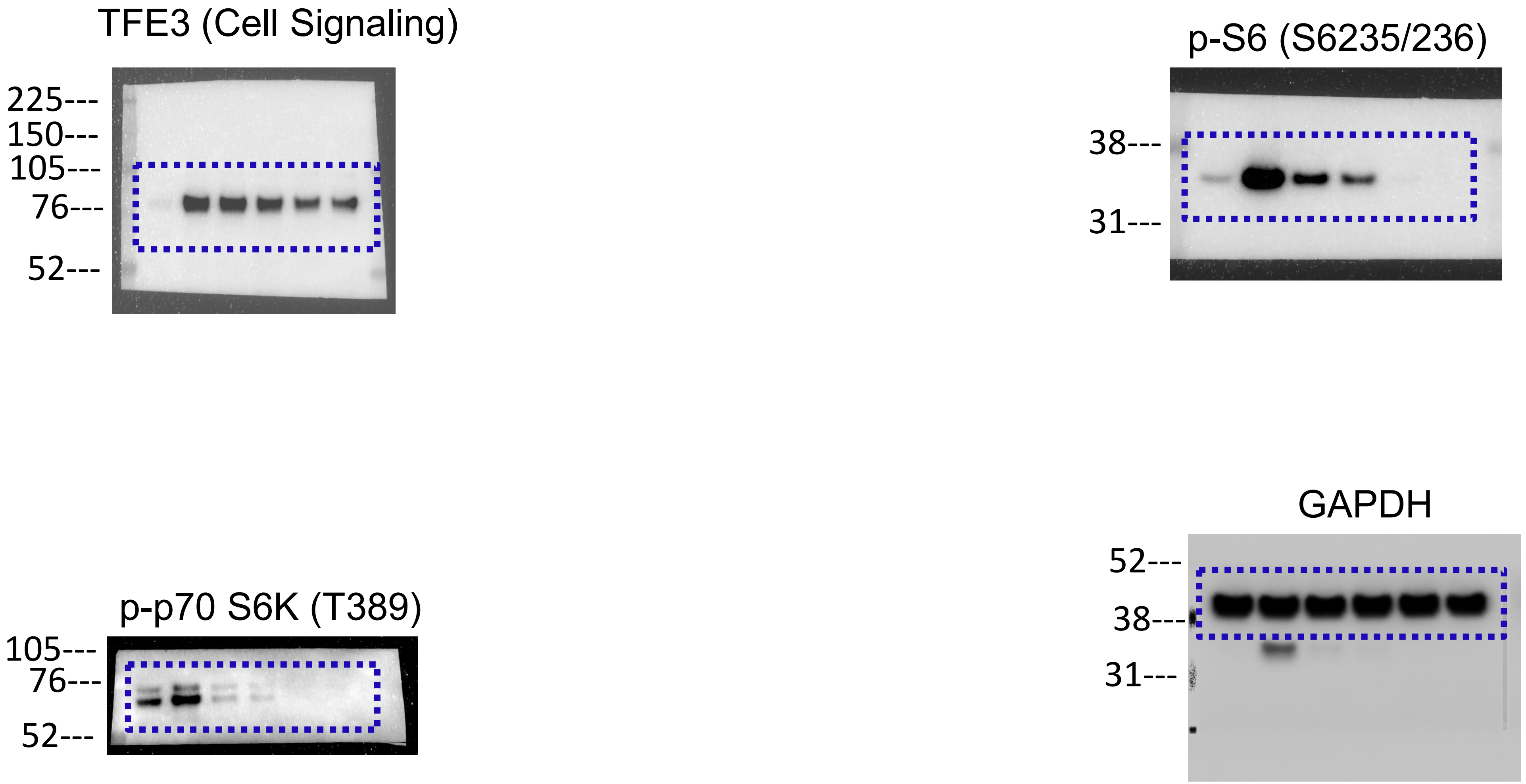
TFE3 (Cell Signaling)
225---
150---
105---
76---
52---
p-S6 (S6235/236)
38---
31---
p-p70 S6K (T389)
105---
76---
52---
GAPDH
52---
38---
31---

FIGURE S9F

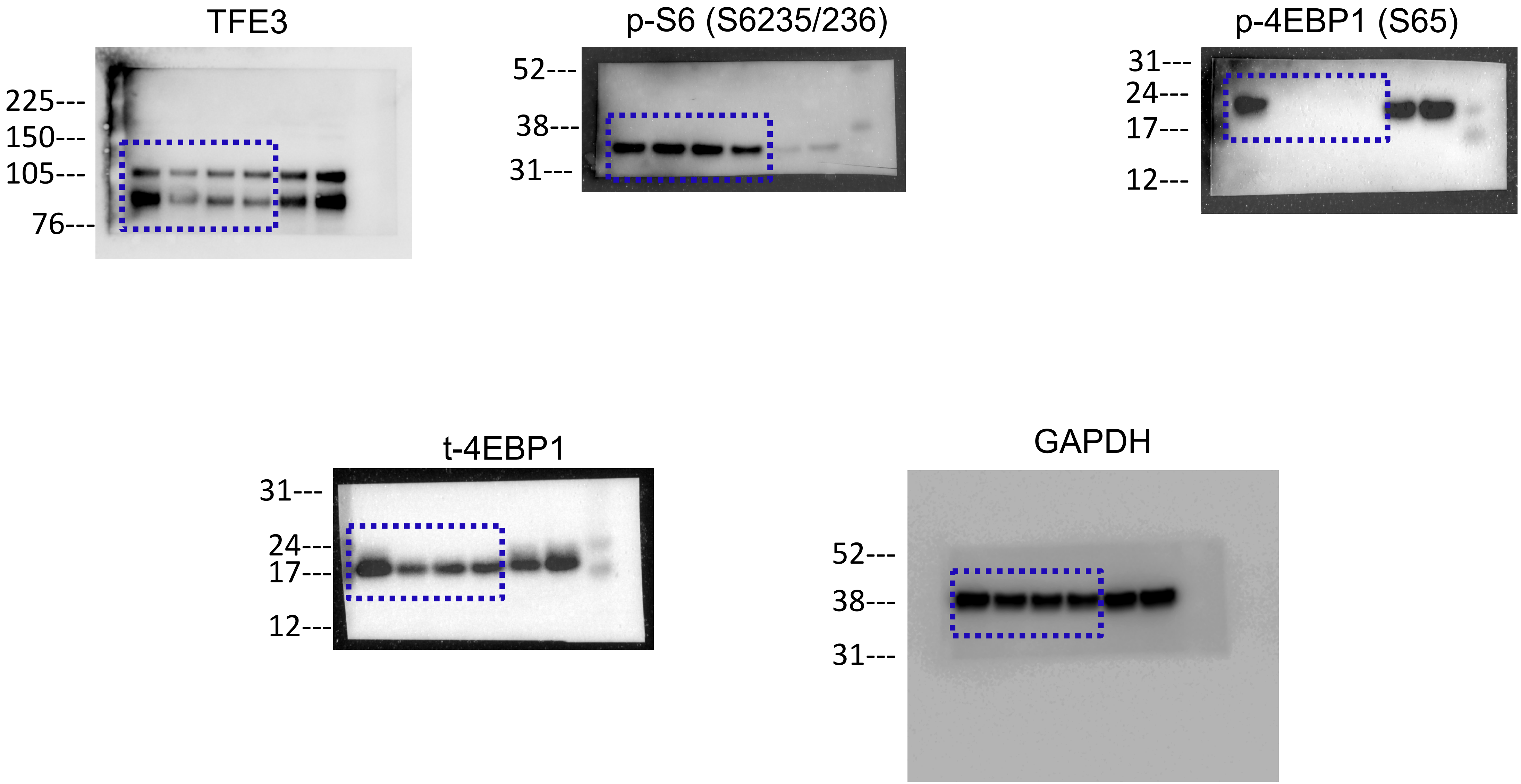
TFE3
225---
150---
105---
76---
p-S6 (S6235/236)
52---
38---
31---
p-4EBP1 (S65)
31---
24---
17---
12---
t-4EBP1
31---
24---
17---
12---
GAPDH
52---
38---
31---

FIGURE S9F

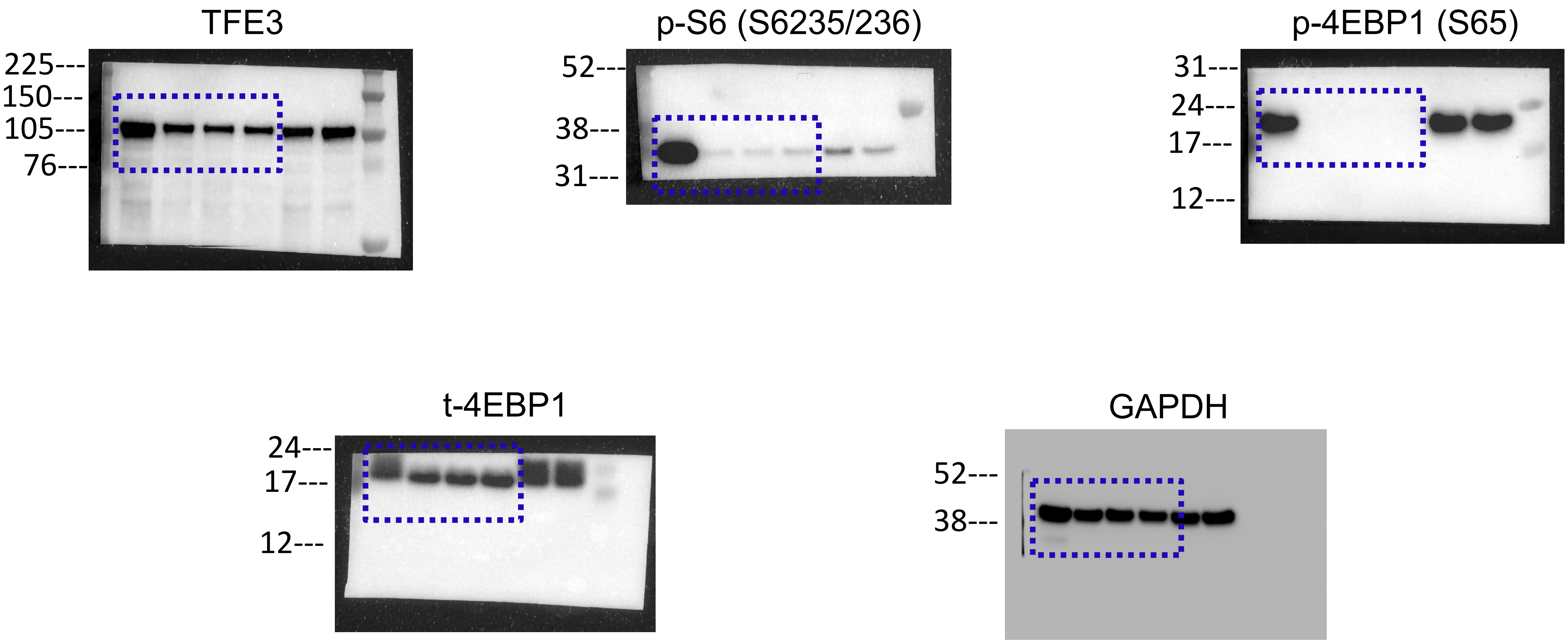
TFE3
225---
150---
105---
76---
p-S6 (S6235/236)
52---
38---
31---
p-4EBP1 (S65)
31---
24---
17---
12---
t-4EBP1
24---
17---
12---
GAPDH
52---
38---

FIGURE S9G

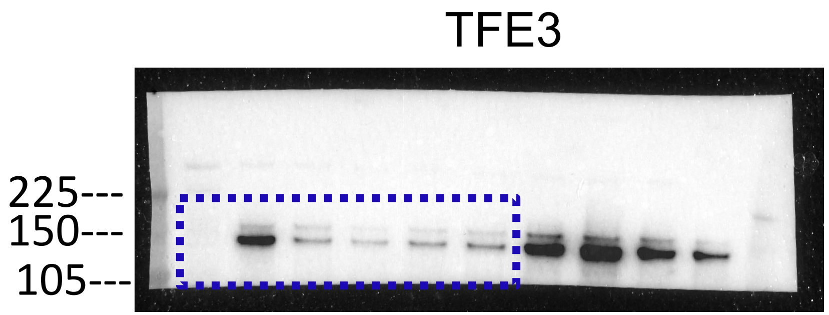

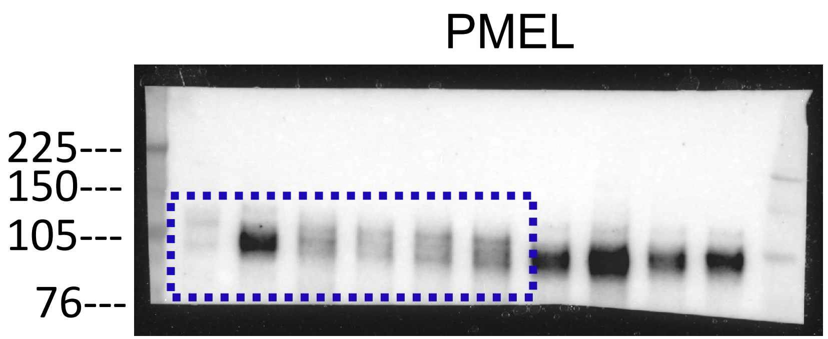

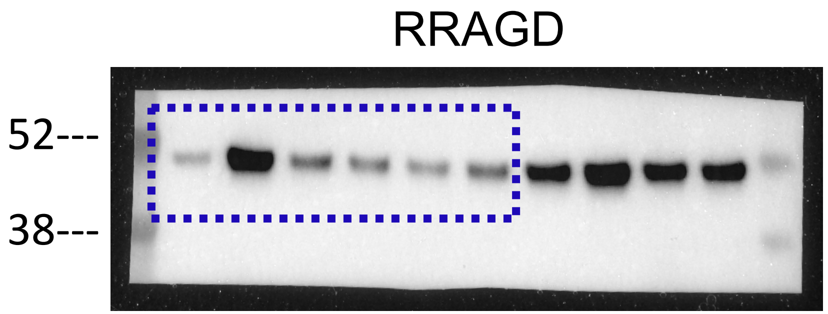

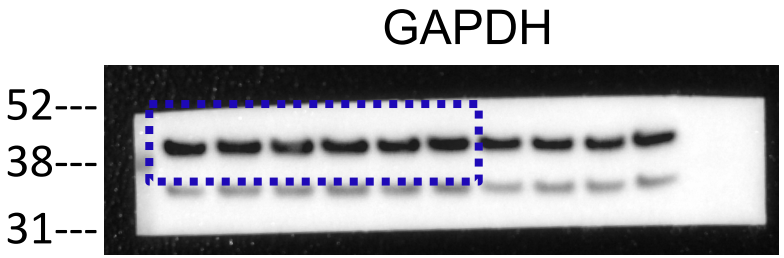

FIGURE S9H

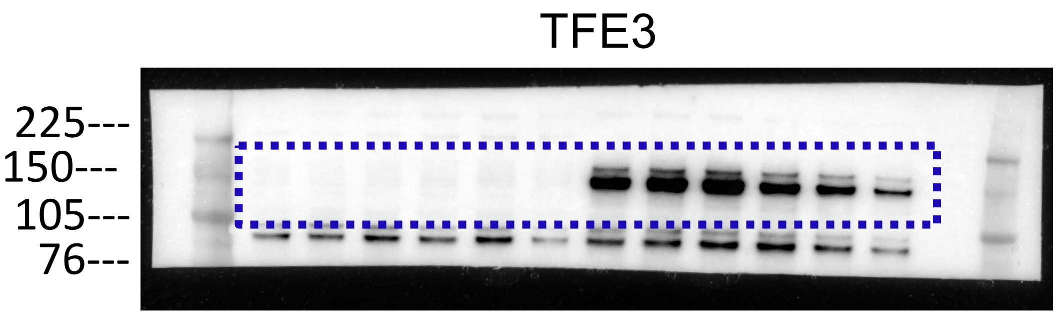

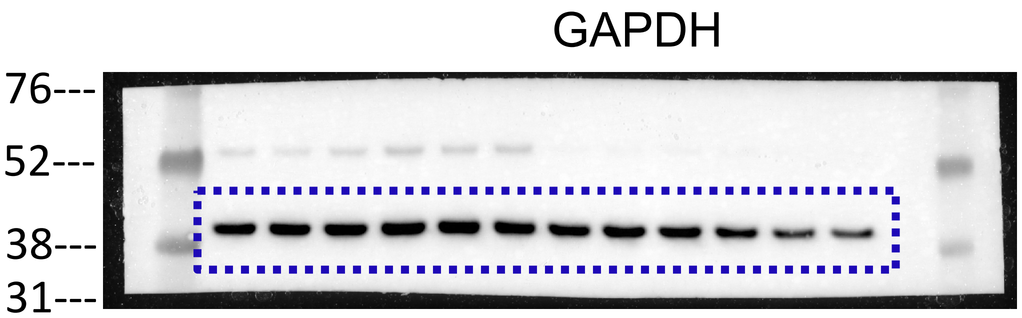

FIGURE S10A

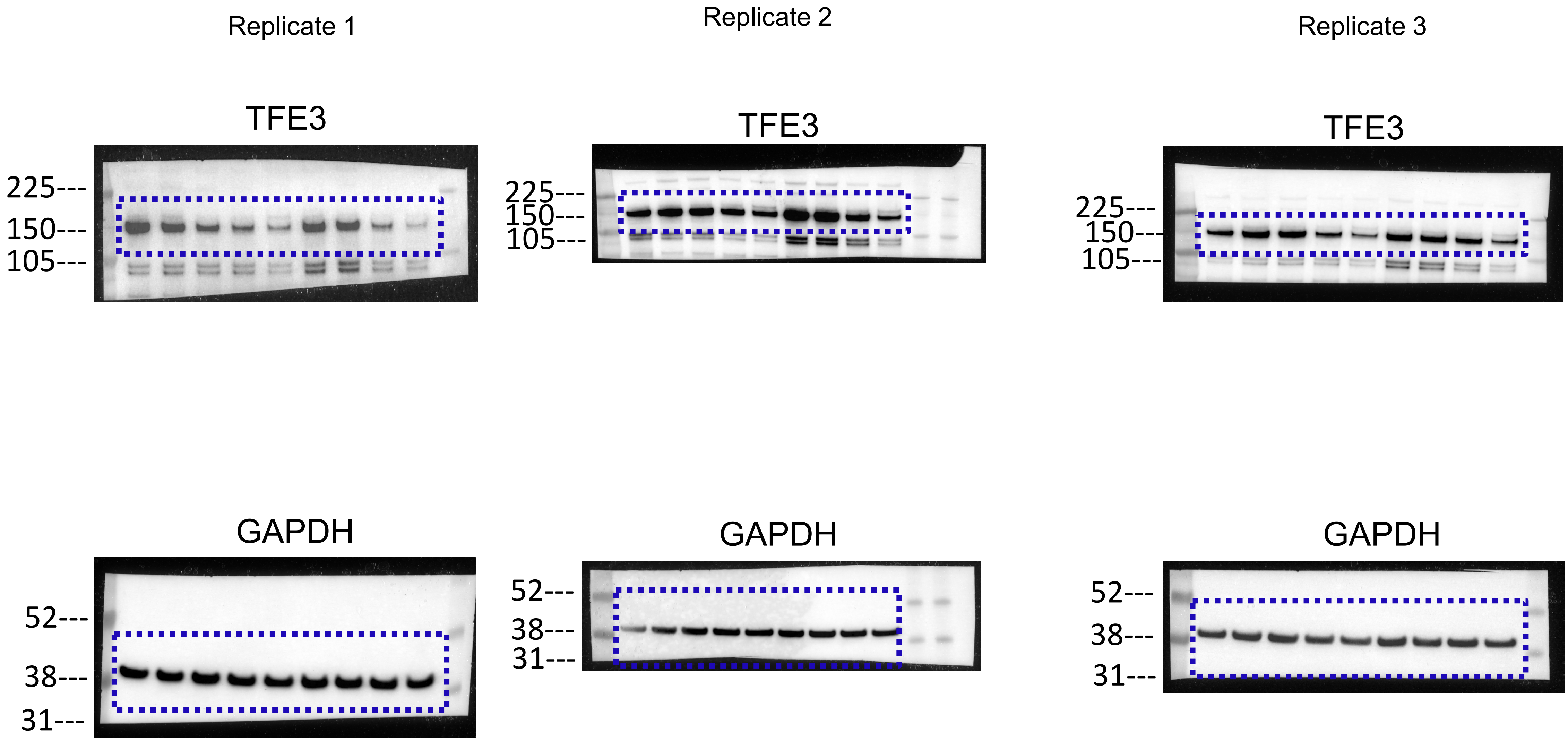
Replicate 1
Replicate 2
Replicate 3
TFE3
225---
150---
105---
TFE3
225---
150---
105---
TFE3
225---
150---
105---
GAPDH
52---
38---
31---
GAPDH
52---
38---
31---
GAPDH
52---
38---
31---

FIGURE S10E

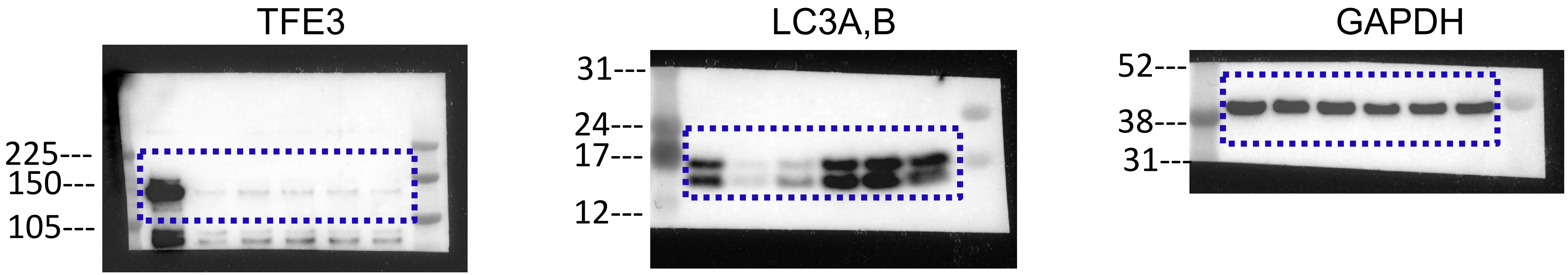

FIGURE S10F (left)

IP: anti-HA

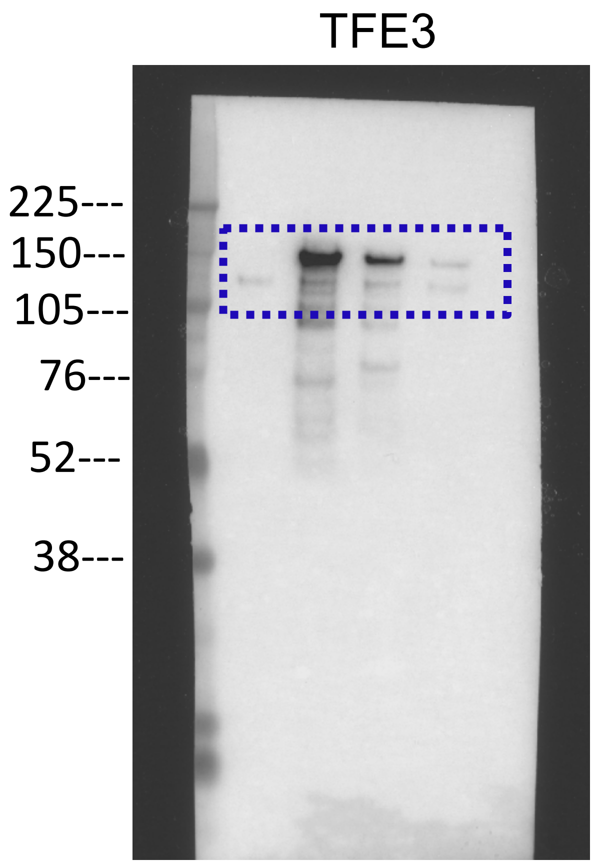

LYSATES

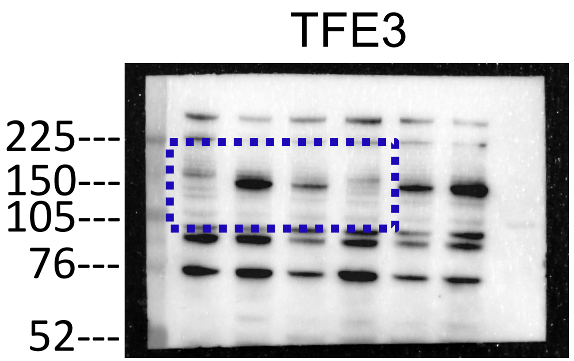

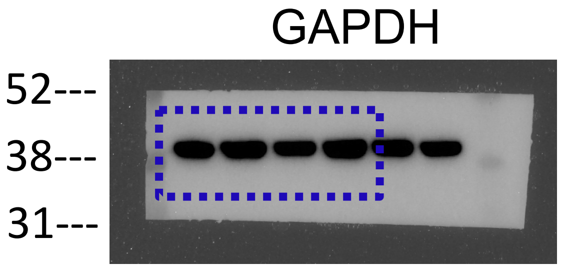

FIGURE S10F (right)

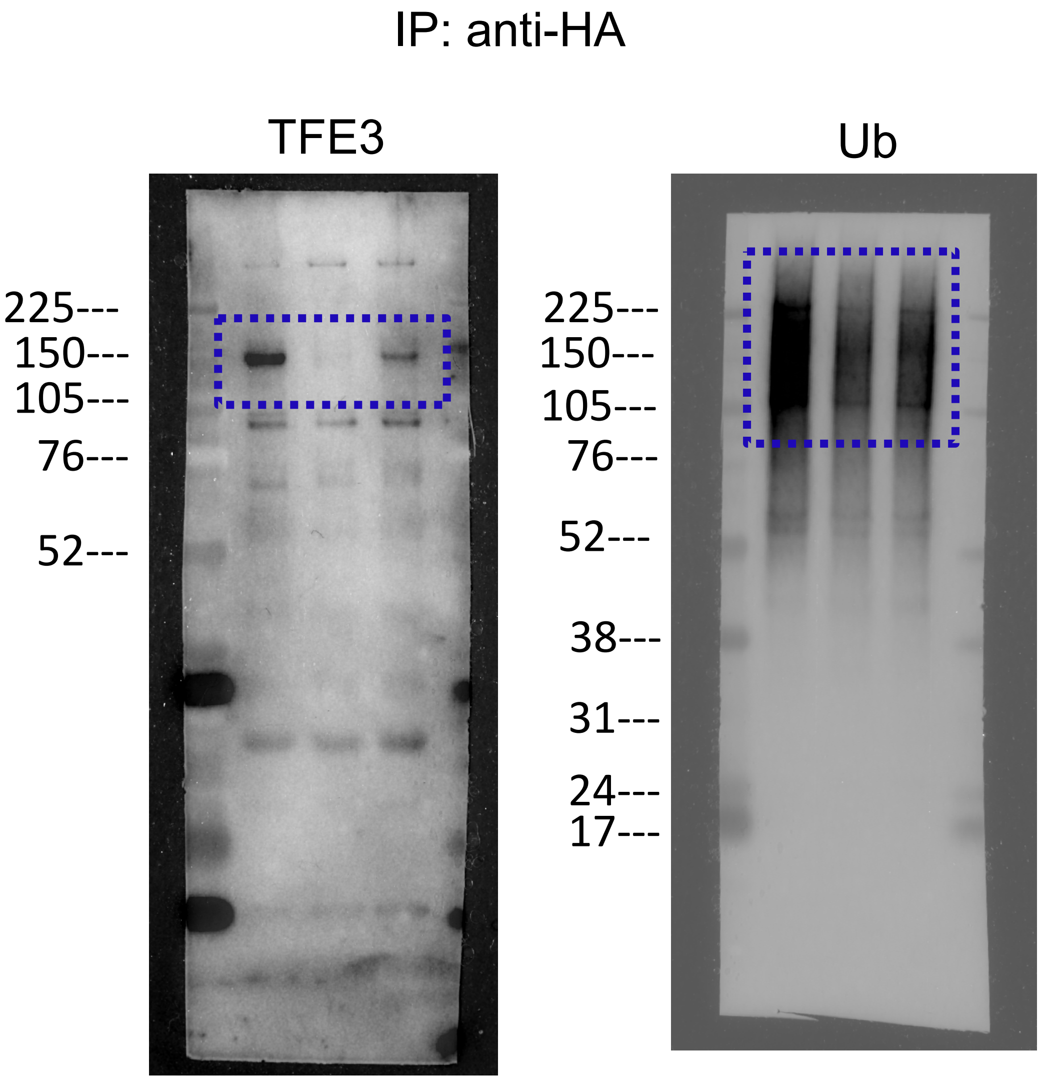

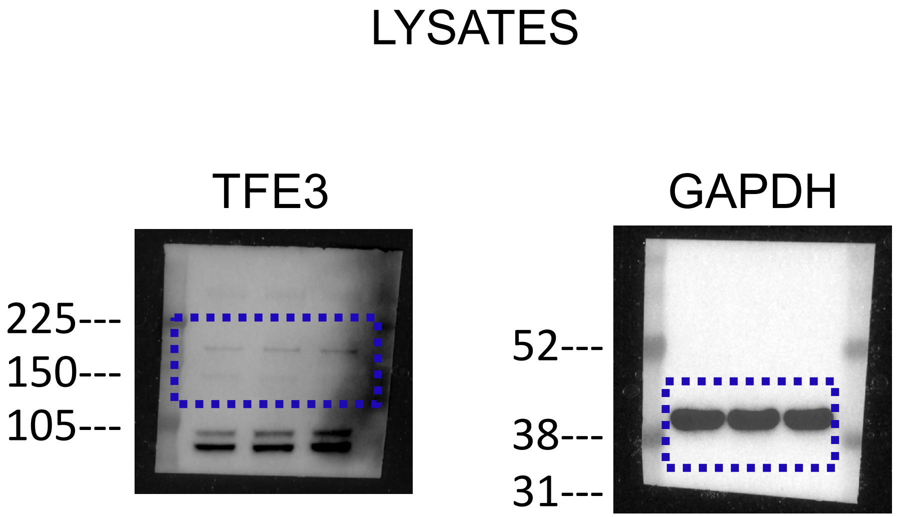

FIGURE S10G

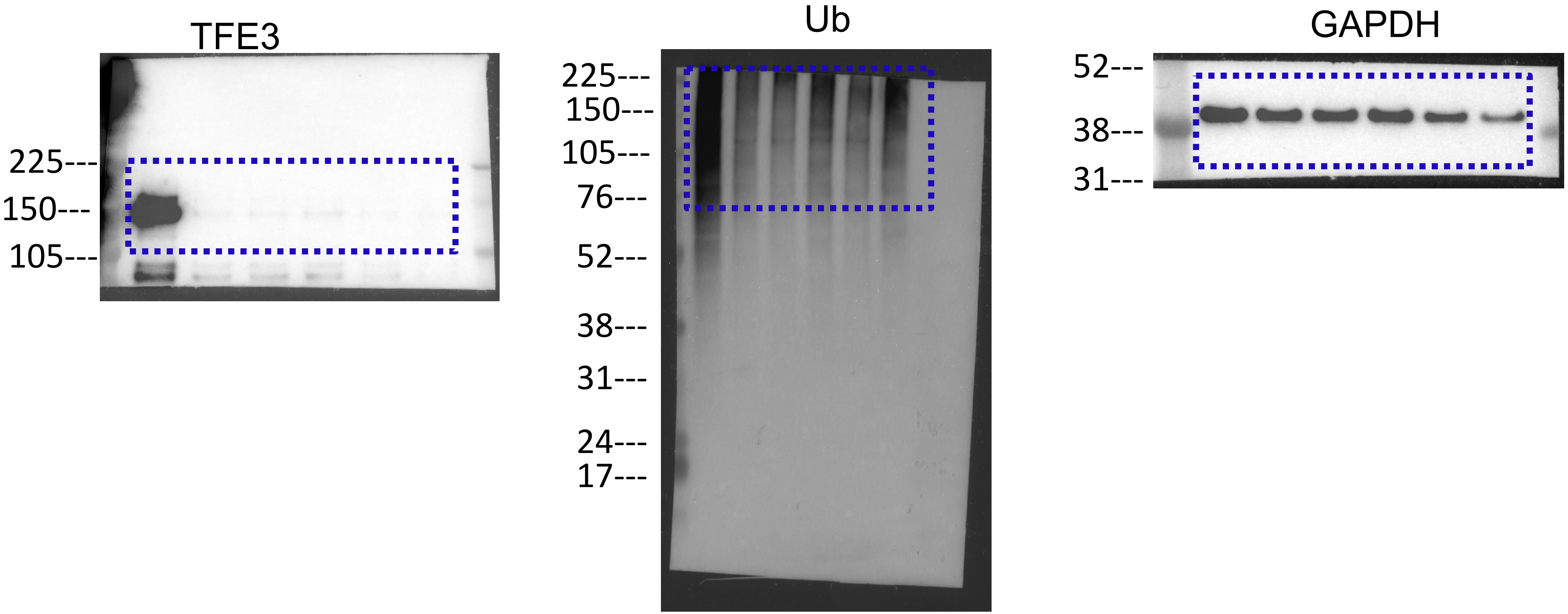

Supplement: Supplementary file 18 — Source Data [file 41467_2025_63885_MOESM18_ESM.zip › NCOMMS-24-79115B_Source Data-Full unedited gels.pdf]
